# ACME dissociation: a versatile cell fixation-dissociation method for single-cell transcriptomics

**DOI:** 10.1186/s13059-021-02302-5

**Published:** 2021-04-08

**Authors:** Helena García-Castro, Nathan J. Kenny, Marta Iglesias, Patricia Álvarez-Campos, Vincent Mason, Anamaria Elek, Anna Schönauer, Victoria A. Sleight, Jakke Neiro, Aziz Aboobaker, Jon Permanyer, Manuel Irimia, Arnau Sebé-Pedrós, Jordi Solana

**Affiliations:** 1 Department of Biological and Medical Sciences, Oxford Brookes University, Oxford, UK; 2 Centre for Genomic Regulation (CRG), Barcelona Institute of Science and Technology (BIST), Barcelona, Spain; 3 Universitat Pompeu Fabra (UPF), Barcelona, Spain; 4 Centro de Investigación en Biodiversidad y Cambio Global (CIBC-UAM) & Departamento de Biología (Zoología), Facultad de Ciencias, Universidad Autónoma de Madrid, Madrid, Spain; 5 School of Biological Sciences, University of Aberdeen, Aberdeen, UK; 6 Department of Zoology, University of Oxford, Oxford, UK; 7 ICREA, Barcelona, Spain

**Keywords:** Single-cell transcriptomics, Planarian, RNA-seq, Fixation, Dissociation, SPLiT-seq, Combinatorial indexing

## Abstract

Single-cell sequencing technologies are revolutionizing biology, but they are limited by the need to dissociate live samples. Here, we present ACME (ACetic-MEthanol), a dissociation approach for single-cell transcriptomics that simultaneously fixes cells. ACME-dissociated cells have high RNA integrity, can be cryopreserved multiple times, and are sortable and permeable. As a proof of principle, we provide single-cell transcriptomic data of different species, using both droplet-based and combinatorial barcoding single-cell methods. ACME uses affordable reagents, can be done in most laboratories and even in the field, and thus will accelerate our knowledge of cell types across the tree of life.

**Supplementary Information:**

The online version contains supplementary material available at 10.1186/s13059-021-02302-5.

## Background

Biology is undergoing a paradigm shift due to the introduction of single-cell sequencing methods [1–5]. The cell is the fundamental unit of biological systems, and studying thousands of them individually allows reconstruction of the cellular diversity and dynamics formerly blended into bulk tissue samples. For instance, single-cell transcriptomics (or scRNA-seq) allows the measurement of the expression of thousands of mRNAs from potentially hundreds of thousands of individual cells. The mRNAs of each cell are indicative of the cell type or state and allow biological questions to be addressed at a new level of integration and detail. From the sequencing of a single cell in 2009 [6], we have seen year-on-year exponential increases in the number of cells that can be sampled by scRNA-seq [7]. Using these methods, scientists have already profiled a broad taxonomic range of different animals, classified their cell types, profiled their gene expression patterns, and begun to reconstruct their cell differentiation lineages. Single-cell transcriptomics has been already used in very diverse animal groups, including sponges [8, 9], cnidarians [10, 11], placozoans [9], ctenophores [9], planarians [12–16], nematodes [17, 18], arthropods [19–22], ascidians [23], and extensively in vertebrates [24–31].

Currently, the most popular methods are based on droplet-based barcoding: encapsulating single cells with oligonucleotide barcodes in nanolitre droplets [32, 33]. One of the most promising recent developments involves employing combinatorial barcoding techniques [17, 34], which use the cells themselves as reaction chambers. These approaches label cellular mRNAs through successive rounds of mixing and pooling the cell population such that the probability of two cells receiving the same barcode combination is minimized. The implementation of combinatorial barcoding methods allows the generation of datasets containing millions of cells from different samples [25, 35].

One major technical hurdle of single-cell transcriptomic approaches is the lack of a cell dissociation method that simultaneously fixes the cells and preserves mRNAs. Typically, dissociation is done in live cells and relies on enzymatic (e.g., trypsin, papain, or similar) or mechanical (e.g., dounce homogenization) approaches [36], which introduce dissociation artifacts and cellular stress on the samples [37–39]. Dissociated cells are stripped from their extracellular context and washed, incubated, centrifuged, stained, and often sorted by FACS while still alive, which changes their gene expression patterns. Preservation can only take place hours after the beginning of the experiment, but this time suffices for the activation of stress responses [38]. The use of cold-active proteases obtained from psychrophilic organisms has been proposed as an alternative approach [40]. Another alternative is obtaining single-cell transcriptomic data from the nuclei [41, 42], as these can be extracted from frozen tissue [25]. However, this approach eliminates the majority of mature mRNAs, as these concentrate outside the nucleus. The introduction of a method that simultaneously fixes and dissociates cells, preserving their RNA, is a critical need of the single-cell transcriptomic field.

To overcome the limitations of live cell dissociations, we have developed ACME dissociation. Our protocol is based on a nineteenth-century dissociation protocol—often called “maceration”—with modifications to make it compatible with modern single-cell transcriptomics. The maceration procedure was first used by Schneider in 1890 [43]. It was then used throughout the twentieth century to dissociate cells of animals such as cnidarians [44] and planarians [45] and observe them under the microscope but is now rarely used [46, 47]. In its original form, the maceration solution simply consisted of acetic acid and glycerol dissolved in water. Baguñà and Romero added methanol as it preserved better morphology [45]. Our protocol uses acetic acid and methanol, together with glycerol, dissolved in water. This solution produces fixed single cells in suspension with high-integrity RNAs. Conveniently, we show that ACME-dissociated cells can be cryopreserved using DMSO [48] at different points throughout the process, with little detriment to their recovery and RNA integrity. We also show that ACME can be used as a fixative, rendering RNA with an integrity superior to that obtained by formaldehyde. As a proof of principle, we have obtained single-cell transcriptomic data from different species and with different single-cell transcriptomic platforms using ACME-dissociated cells. First, we obtained 3899 cells from the cnidarian *Nematostella vectensis*, using a droplet-based method. With this, we recover all major cell types described in a previous study [10]. Second, we combined ACME with a modified version of split pool ligation-based transcriptome sequencing (SPLiT-seq) [34], a combinatorial indexing method, and were able to profile 33,827 cells from two different planarian species, *Schmidtea mediterranea* and *Dugesia japonica*, in a single run. We recover all *S. mediterranea* cell types from a previous study [13], at comparable proportions, showing that ACME dissociation does not introduce biases in cell type composition. Furthermore, we describe for the first time the single-cell transcriptome of *D. japonica*, opening the study of cell type evolution in this clade. We integrate our analysis with previous *S. mediterranea* data obtained by trypsin dissociation, showing that the datasets are broadly compatible and can be integrated in a straightforward manner. Altogether, in combination with droplet-based or combinatorial barcoding platforms such as SPLiT-seq, ACME dissociation is a robust method to obtain high-quality single-cell transcriptomic data from fixed cells.

## Results

### ACME dissociation produces fixed cells with preserved morphology that can be visualized by flow cytometry

**Fig. 1:**
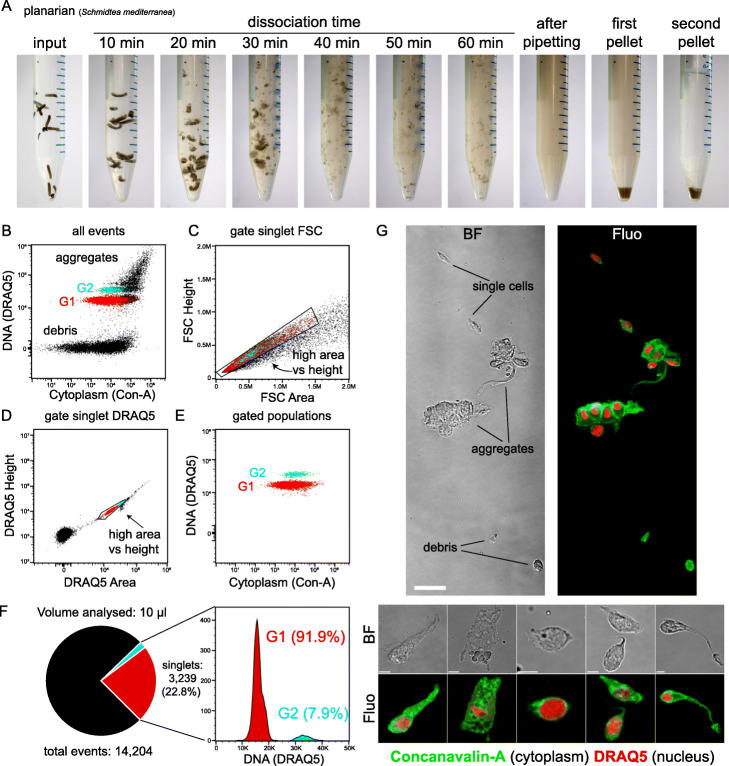
ACME cell dissociation and fixation. **a** Whole dissociation process for the planarian *Schmidtea mediterranea*. From left to right: live worms used as input in water, ACME dissociation reaction after 10–60 min, cell suspension after final pipetting, pellet after first centrifugation, and pellet after second centrifugation in PBS 1% BSA. **b**–**e** Flow cytometry profiles of *S. mediterranea* ACME-dissociated cells stained with DRAQ5 (nucleus) and Concanavalin-A (cytoplasm): ungated (**b**), after gating singlets by FSC (**c**), and DRAQ5 (**d**). Area vs height, and resulting clean G1 (red) and G2 (cyan) populations. Singlets are selected based on their well-correlated area vs height signal, while aggregates display high area vs height ratios. **f** Relative proportion of singlets in a typical *S. mediterranea* ACME cell dissociation, corresponding to 22.8% of the total events, and histogram of their DNA content (linear scale), showing the relative proportions of G1 and G2 cells. **g** Bright field (BF) and confocal fluorescence (Fluo) microscopy images of *S. mediterranea* ACME-dissociated cells stained with Concanavalin-A and DRAQ5, showing single cells, aggregates, and debris (top) and details of different cell types with well-preserved morphology (bottom). Scale bars are 50 μm (top) and 5 μm (bottom)

ACME dissociation takes place in ~ 1 h (Fig. 1a). We immerse ~ 10–15 adult *S. mediterranea* individuals or a similar amount of other tissue (representing ~ 100 μL of biological material) in 10 mL of ACME solution. An optional initial washing step in *N*-acetyl-l-cysteine (NAC) prior to ACME dissociation helps remove the mucus [49, 50] (see the “Methods” section). Once animals are in ACME solution, they are shaken for 1 h at room temperature, with occasional pipetting up and down of the solution to aid dissociation. We then collect the cells by centrifugation to remove the ACME solution (Fig. 1a) and wash the pellet in cold conditions in a PBS solution containing 1% BSA. We perform a second centrifugation as a subsequent cleaning step and, finally, resuspend the cells in the same buffer solution (Fig. 1a). After this step, cells must be kept in PBS/1% BSA solution in cold conditions (i.e., on ice).

In order to visualize cells by flow cytometry, we stain fixed cells with DRAQ5 (nuclei) and Concanavalin-A conjugated with Alexa Fluor 488 (cytoplasm). DRAQ5 is a far-red emitting anthraquinone compound that stains DNA. Concanavalin-A is a lectin that binds carbohydrates present in internal cell membranes. Since ACME cells are permeabilized, we find Concanavalin-A staining throughout the cytoplasm. These staining conditions reveal several cell populations (Fig. 1b). The lowest DNA-containing population corresponds to cells with 2C DNA content and includes G1 and G0 cells (hereafter “G1”). The cell population above contains G2/M cells with 4C DNA content (hereafter “G2”). S-phase cells are difficult to resolve with these staining conditions. G2 corresponds to what planarian FACS protocols typically refer to as the “X1” population [51] and is sensitive to irradiation (Additional file 1: Figure S1A-B). Like other dissociation protocols, ACME also produces a large quantity of cellular debris, with cytoplasm staining but without DNA (Fig. 1b). Undissociated cell aggregates are also visible, with higher levels of DNA and cytoplasm staining (Fig. 1b). When compared to the classic trypsin dissociation protocol [51], ACME-dissociated cells display more aggregates, but less cellular debris (Additional file 1: Figure S1C). Ultimately, all dissociation methods generate variable amounts of aggregates and debris, but these can be excluded due to their cytometric profiles. To distinguish singlets from doublets and other aggregates, we use a singlet filter: aggregates are gated out by their increased area signal compared to the height. This can be achieved using either the FSC (Fig. 1c) or the DRAQ5 (Fig. 1d) area vs height signal, or both, by selecting events with well-correlated signal area and height values. Then, gating DRAQ5-positive cells (DRAQ5 area vs FSC area) excludes cellular debris to obtain clear G1 and G2 populations (Fig. 1e).

Typically, we resuspend one dissociation reaction in 1 mL of buffer. The analysis of 10 μL of such reactions reveals thousands of singlet cells (Fig. 1f) that can be FACS-sorted. The relative proportions of G1 vs G2 cells are similar to those described in planarians by enzymatic approaches [51] (Fig. 1f, right). ACME-dissociated cells also exhibit well-preserved morphology under microscopic observation (Fig. 1g), as this was the original purpose of the maceration technique [43–45].

### ACME is a species-versatile method that can be used in a broad range of animals and developmental stages

**Fig. 2:**
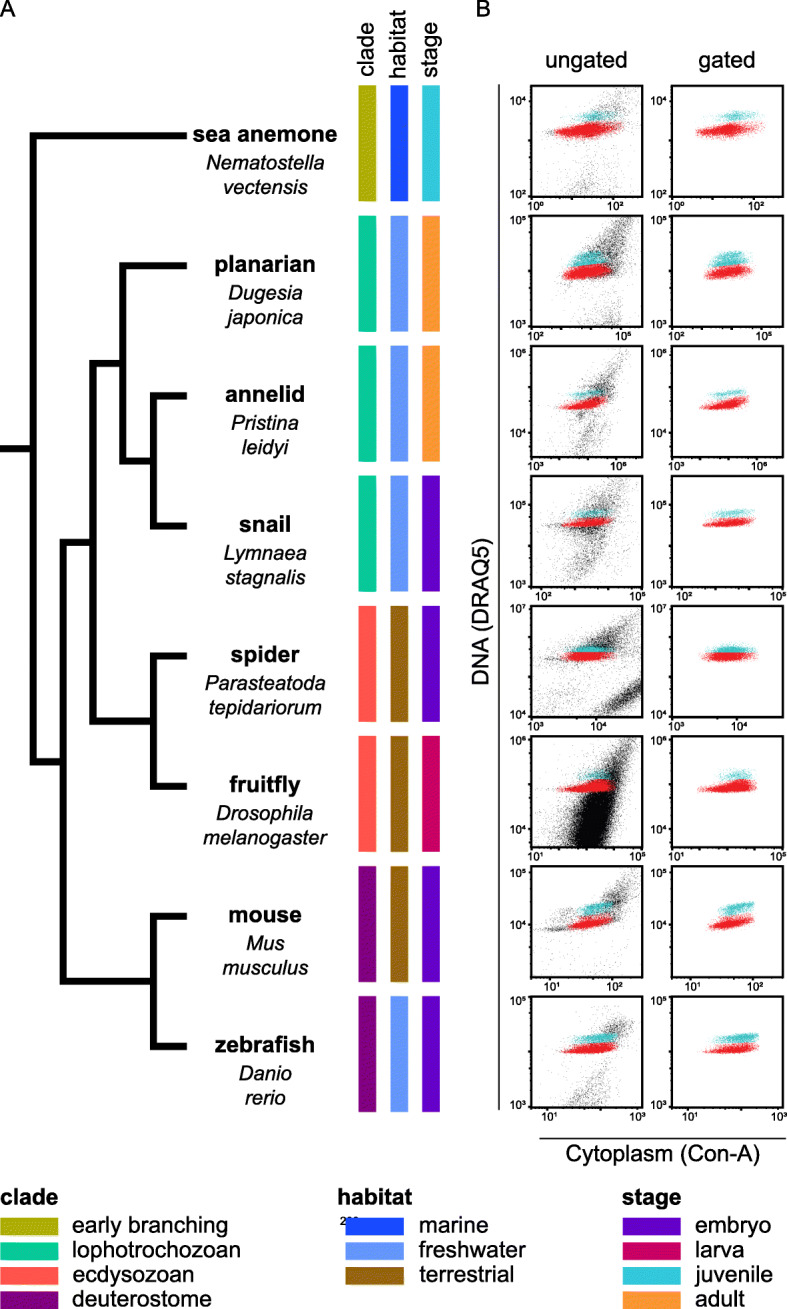
Species versatility of ACME cell dissociation. **a** Visual representation of the phylogenetic distribution, habitat, and stage of the animals where ACME dissociation was assayed: sea anemone juveniles (*Nematostella vectensis*), planarians (*Dugesia japonica*), annelid adults (*Pristina leidyi*), snail larvae (*Lymnaea stagnalis*), spider stage 7 embryos (*Parasteatoda tepidariorum*), fruitfly 3rd instar stage larvae (*Drosophila melanogaster*), mouse E11.5 embryos (*Mus musculus*), and zebrafish 1-day embryos (*Danio rerio*). **b** Flow cytometry ungated and gated profiles of ACME-dissociated cells from different organisms stained with DRAQ5 (nucleus) and Concanavalin-A (cytoplasm). Axes are shown in subset logarithmic scales

To test if ACME can be used in different species, we used it to dissociate several animals, including the sea anemone *N. vectensis*, the planarian *D. japonica*, the annelid *Pristina leidyi*, the snail *Lymnaea stagnalis*, the spider *Parasteatoda tepidariorum*, the fruitfly *Drosophila melanogaster*, the mouse *Mus musculus*, and the fish *Danio rerio* (Fig. 2a). This set of animals includes organisms belonging to diverse major metazoan lineages, including early-branching metazoans, lophotrochozoans, ecdysozoans, and deuterostome animals. Furthermore, it encompasses animals from a broad range of terrestrial, freshwater, and marine habitats. It also includes several life stages, such as embryos, larvae, juveniles, and adults. Therefore, ACME dissociation is a versatile method that can be used in markedly different animal models. We use the same protocol for all organisms with minimal alterations. ACME solution dissociates soft tissues and cannot penetrate hard shells, chorions, or vitelline membranes. We dechorionated zebrafish embryos using standard protocols and ruptured the cocoons and vitelline membranes that encapsulate spider and snail embryos. This can be done after embryos are placed in the ACME solution, using forceps under the scope or with short pulses of homogenization. Soft-bodied animals, like planarians, completely dissociate with minimal mechanical forces (shaking and pipetting up and down), but other animals such zebrafish or cnidarians benefit from stronger mechanical dissociation using a combination of homogenization and dissection. We successfully obtained clear singlet populations from these animals using DRAQ5 and Concanavalin-A as fluorescent stains with similar gating conditions (Fig. 2b). Adjustments to the dissociation protocol (acid concentration, time of incubation, mechanical dissociation), staining (dyes and concentrations), and cytometry detection (gating) may improve the purification of single cells from other organisms. Our protocol provides a solid starting point for the optimization of ACME in these. Altogether, these experiments show that ACME is a species-versatile cell dissociation approach that can be used in a wide range of species.

### ACME-dissociated cells can be cryopreserved multiple times

Currently, single-cell dissociation protocols typically rely on enzymatic (e.g., trypsin) dissociation. One disadvantage of enzymatic approaches is that cells can only be cryopreserved after dissociation, typically by FACS sorting them into methanol [52] or DMSO-containing solutions [46]. However, cells have already been out of their cellular context for several hours. Apart from biological effects, this also imposes logistical restrictions: cell dissociation needs to be done in close proximity to a single-cell transcriptomic facility or at least a FACS facility. This logistical restraint renders single-cell analysis of specimens collected in remote sampling areas, or that are difficult to culture in the laboratory, extremely challenging.

**Fig. 3:**
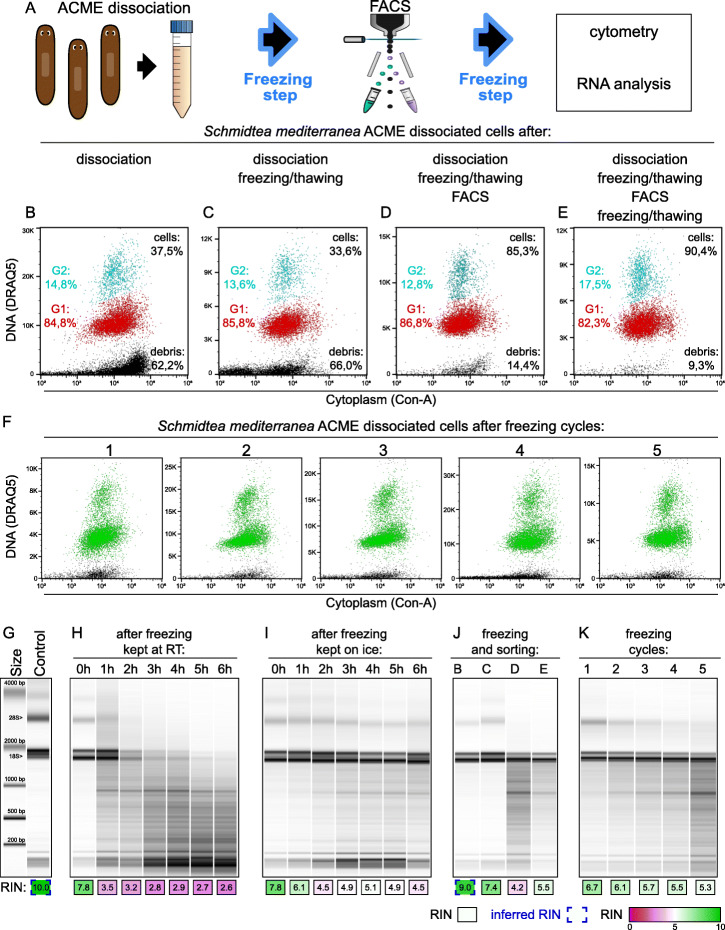
Cryopreservation and RNA integrity of ACME-dissociated cells. **a** Experimental workflow of ACME dissociation for cytometry and RNA analysis. **b**–**e** Flow cytometry profiles of singlet gated (FSC) *Schmidtea mediterranea* ACME-dissociated cells, stained with DRAQ5 (DNA) and Concanavalin-A (cytoplasm), directly after dissociation (**b**), after a first freezing step (**c**), after FACS (**d**), and after a second freezing step (**e**). DRAQ5 scales are shown in linear values to focus on G1 and G2 populations and differ due to the freezing steps. Aggregates are gated out by FSC. Percentages relative to the number of total singlets are shown in black for debris and cells. G1 (red) and G2 (blue) percentages refer to these population proportions. G1 and G2 proportions do not greatly vary, but FACS sorting effectively enriches these populations. **f** Flow cytometry profiles of singlet gated (FSC) *S. mediterranea* ACME-dissociated cells after 1 to 5 freezing cycles. DRAQ5 and Concanavalin-A-positive cells are shown in green and debris in black. Scale and gating conditions as in **b** and **c**. **g**–**k** Bioanalyzer profiles and RIN values of RNA samples. Inferred values (Inf RIN, blue open boxes) are calculated from a correlative analysis of the % area of the two ribosomal bands compared to the total as shown in Additional file 1: Figure S2. As a control sample, we used RNA from worms directly in TRIzol (**g**). A size ladder is displayed, and the two major RNAs (18S and 28S) are indicated. Time- and temperature-dependent RNA degradation were tested keeping samples at room temperature (**h**) or on ice (**i**) for 6 h. RNA integrity along the protocol was tested for the conditions described in **b**–**e** (**j**), showing partial degradation after FACS. RNA integrity was tested after 1 to 5 freeze/thaw cycles (**k**)

Our protocol avoids lengthy live incubations, as ACME fixes cells immediately. Furthermore, we found that ACME-dissociated cells can be easily cryopreserved multiple times by freezing them in a PBS solution containing BSA (1%) and DMSO (10%) [48]. To test this, we compared ACME-dissociated cell populations after several freezing steps (Fig. 3a). We analyzed cell populations by flow cytometry right after dissociation (Fig. 3b) and after freezing and thawing the cells (Fig. 3c). We also FACS-sorted ACME-dissociated cells resulting in an 85–90% enrichment of G1 and G2 cells. We compared FACS-sorted cells directly after sorting (Fig. 3d) with FACS-sorted cells cryopreserved again after sorting (Fig. 3e). This shows that ACME-dissociated cells can be subjected to several rounds of cryopreservation without altering their cytometry profiles. To test resistance to freezing, we subjected ACME-dissociated cells to 5 freeze/thaw cycles and analyzed the resulting populations (Fig. 3f). We found no differences in the cytometry profiles after multiple steps of cryopreservation, showing that ACME is a robust and convenient method to obtain and preserve dissociated cells.

### ACME-dissociated cells retain high-integrity RNAs

We next tested if RNAs are well preserved in ACME-dissociated cells, a critical requirement for single-cell transcriptomics. We added NAC to the ACME solution as it provides reducing conditions that protect RNA from degradation [49, 50] (see the “Methods” section). To test RNA integrity, we ran our samples in a Bioanalyzer and obtained RNA integrity number (RIN) values. One caveat of this measurement is that it is based on a Bayesian learning approach, and the samples used to train this algorithm were exclusively vertebrate [53]. As a result, the Bioanalyzer software sometimes fails to calculate RIN values of non-vertebrate samples as the peak shapes do not match training sets [53, 54]. Moreover, a phenomenon known as the “hidden break” in the 28S ribosomal RNA causes it to break in two pieces of approximately the same size as the 18S [55, 56], causing a sharp decrease in the RIN value of the sample that is not due to RNA degradation. This is triggered by denaturation, heating, or chemicals [55, 56] and has been described to be widely present in platyhelminthes, including *Schmidtea mediterranea*, and many other animal groups [54]. As a result, RIN numbers of ACME samples are never above 8. These facts make the evaluation of RNA integrity using RIN numbers challenging. We registered RIN values when possible, but also inferred them when we failed to obtain them, by calculating the signal area taken up by the two 28S and 18S ribosomal bands compared to the total RNA signal area. This measure robustly correlates with the RIN value (Figure S2A). Then, inferred values were calculated using a linear regression of this correlation (Figure S2B). This is a straightforward and robust approach to evaluate RNA integrity in invertebrate RNA samples.

We found that the major factors affecting RNA quality in ACME-dissociated cells are time and temperature. To test the degree of impact of this, we extracted RNA from ACME-dissociated cells in different conditions and compared it to RNA from undissociated planarians (Fig. 3g). RNA degradation at room temperature was tested by incubating ACME-dissociated cells in PBS containing 1% BSA for several hours (Fig. 3h). We observed that RNA integrity after ACME dissociation progressively declines over time at room temperature. Keeping cells on ice effectively prevents this effect (Fig. 3i). Therefore, cold conditions are sufficient to safeguard RNA for ongoing work. FACS-sorted cells also show some RNA degradation (Fig. 3j), as the total time required for our staining and FACS sorting is about ~ 3–6 h. Repeated freeze/thaw cycles also negatively impact RNA integrity (Fig. 3k) but do not result in complete degradation. These results show that ACME-dissociated cells can be cryopreserved multiple times with little detriment to recovery and RNA integrity. Therefore, ACME dissociation is a convenient method to obtain and cryopreserve large sample sets and to obtain single-cell suspensions from animals difficult to culture in the lab, or directly from the wild.

### ACME can be used as a fixative of enzymatic dissociated cells

**Fig. 4:**
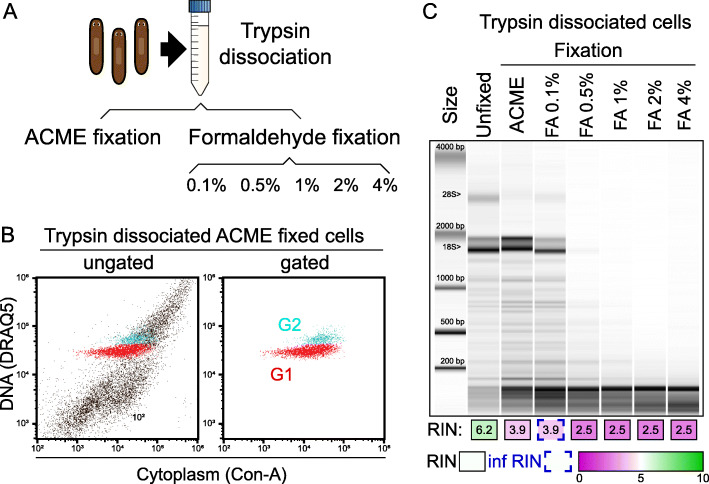
Comparison of ACME and formaldehyde as cell fixation reagents. **a** Experimental workflow of trypsin dissociation with ACME and formaldehyde fixation. **b** Flow cytometry ungated and gated profiles of trypsin-dissociated ACME-fixed cells stained with DRAQ5 (nucleus) and Concanavalin-A (cytoplasm). **c** Bioanalyzer profiles and RIN values of RNA samples dissociated with trypsin and fixed with ACME or increasing concentrations of formaldehyde (FA). Inferred values (Inf RIN, blue open boxes) are calculated from a correlative analysis of the % area of the two ribosomal bands compared to the total. As a control sample, we used RNA from worms dissociated with trypsin and introduced unfixed in TRIzol

We next tested if ACME can act as a fixative of trypsin-dissociated cells. We dissociated animals using trypsin as previously described [51] and fixed the resulting cells with ACME or with increasing concentrations of formaldehyde (Fig. 4a). After trypsin dissociation and ACME fixation with minor modifications (see the “Methods” section), we recovered cells that pelleted normally. We inspected these cells using cell cytometry and were able to recover G1 and G2 populations with our normal staining and gating conditions (Fig. 4b). This indicates that ACME can be used as a fixative of trypsin-dissociated cells. These cells can later be subjected to FACS enrichment. To test the RNA integrity of these cells, we compared trypsin-dissociated cells fixed with ACME to trypsin dissociated cells fixed with formaldehyde (Fig. 4c). The latter has been used to perform single-cell transcriptomic experiments, particularly by combinatorial methods [17, 25, 34], but it is known to result in poor RNA integrity, which can make sequencing approaches challenging [57]. Trypsin-dissociated cells fixed with ACME preserve RNA integrity better than formaldehyde fixation, except at the lowest formaldehyde concentration used, 0.1% (Fig. 4c). Arguably, at this concentration, cells are underfixed, as formaldehyde is typically used at 1–4% concentrations. These experiments show that ACME can be used as an alternative to formaldehyde fixation of enzymatic dissociations.

### Single-cell transcriptomic analysis of cnidarian ACME-dissociated cells using a droplet-based method

**Fig. 5:**
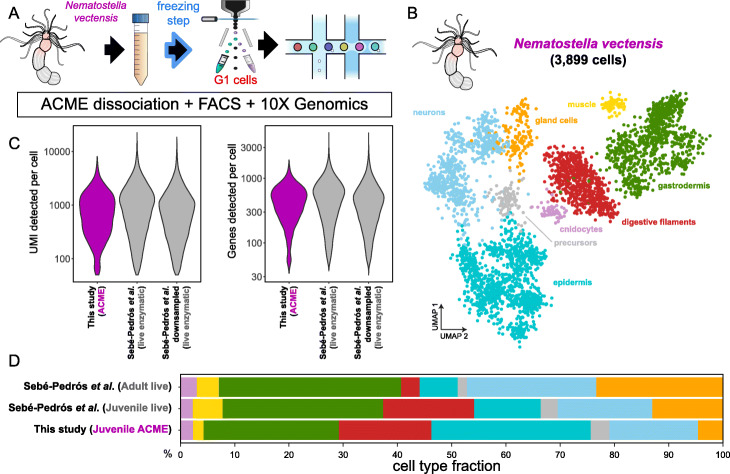
Droplet-based single-cell transcriptomic analysis of ACME-dissociated cells from the cnidarian *Nematostella vectensis*. **a** Experimental workflow. We used ACME-dissociated and FACS-sorted G1 cells from the cnidarian species *N. vectensis*, after one freezing step. For droplet-based single-cell transcriptomic analysis, we used the 10X Genomics Chromium platform. **b** UMAP visualization of 3899 *N. vectensis* cells, colored by cluster identity and annotated on the basis of marker genes. **c** Violin plots showing the distribution of UMI counts and genes detected per cell. **d** Comparison of cell proportions for *N. vectensis* with a previous cell type atlas (Sebé-Pedrós et al.). Cell clusters are grouped by cell type

Droplet-based methods of single-cell transcriptomics [32, 33] dominate the current literature [7]. To test if ACME-dissociated cells can be profiled by droplet-based methods, we used the 10X Genomics Chromium technology. We used juvenile individuals of the cnidarian *Nematostella vectensis*, as its cell type atlas has been profiled previously [10]. We performed ACME dissociation with one freezing step and used FACS to enrich for differentiated cell types (Fig. 5a, Additional file 1: Figure S3A). We obtained 3899 cells which recapitulated the major known cell types of *N. vectensis* [10] (Fig. 5b). These broad cell type identities were supported by the expression of dozens of transcription factors, for example, Jun bZIP in cnidocytes, SCX/TCF15 in retractor muscle cells, Rfx4/6/8 in digestive filaments, and ASC and Pou5 in neurons (Additional file 1: Figure S3B). Our ACME-dissociated dataset recovered a median of 671 UMI per cell and 418 genes per cell (Fig. 5c, Additional file 1: Figure S3C), resembling the statistics obtained in the previous study (833 UMI per cell and 464 genes per cell, and 696 UMI per cell and 381 genes per cell when downsampled to a comparable number of reads) generated using enzymatic (Liberase) dissociations [10] and MARS-seq [58]. A recent single-cell transcriptomic study of *N. vectensis* cells dissociated enzymatically and barcoded using 10X Genomics technology had comparable statistics [59]. We then compared the cell type abundances that we obtained with the previous dataset, showing similar relative proportions (Fig. 5d). The main difference observed was a higher fraction of epidermal cells in the ACME-based single-cell experiment.

### Single-cell transcriptomic analysis of planarian ACME-dissociated cells using combinatorial barcoding

**Fig. 6:**
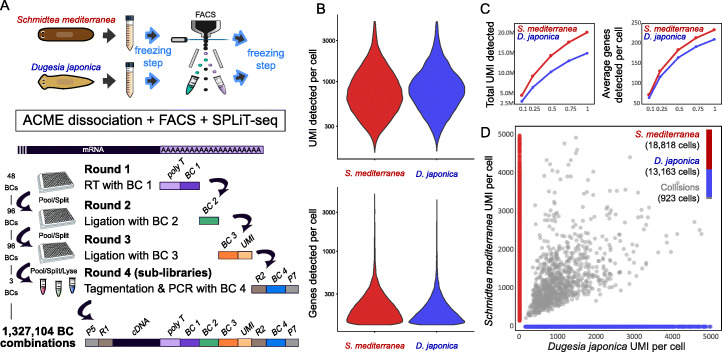
SPLiT-seq single-cell transcriptomic analysis of ACME-dissociated cells, overview, and metrics. **a** Experimental workflow. We used ACME-dissociated and FACS-sorted cells from the planarian species *S. mediterranea* and *D. japonica*, after two freezing steps. For SPLiT-seq, combinatorial barcoding consisted of 4 rounds of barcoding with 48 × 96 × 96 × 3 barcodes. cDNA molecules coming from each cell are uniquely labeled by one of the 1,327,104 possible barcode combinations. **b** Violin plots showing the distribution of UMI counts and genes detected per cell. **c** Saturation plots for UMIs per cell (left) and genes per cell (right) at given fractions of the complete sequencing depth, for the 19,741 and 14,086 single-cell transcriptomes sequenced above the threshold for *S. mediterranea* and *D. japonica*, respectively. **d** Scatter plot of *S. mediterranea* (red) vs *D. japonica* (blue) UMI counts per cell. Collisions are shown in gray

ACME-dissociated cells are fixed and permeabilized and, therefore, could be used coupled to combinatorial barcoding single-cell transcriptomic approaches. These methods allow profiling a higher number of cells and have lower costs per cell, and furthermore do not require specialized microfluidic devices. In combinatorial barcoding methods, cells themselves are used as compartments where barcoding reactions take place. To test the potential of ACME-dissociated cells in combinatorial barcoding protocols, we performed a species mixing experiment (Fig. 6a) using SPLiT-seq [34] to profile two different planarian species: *S. mediterranea* and *D. japonica*. Briefly, pools of cells are split equally into wells, labeled with different reactions, and then pooled again (Fig. 6a, Additional file 1: Figure S4A). After four barcoding rounds, the probability of any two cells receiving the same barcode combination is minimized. The SPLiT-seq protocol consists of an in-cell retrotranscription (RT) and two rounds of oligo ligations, with the fourth barcode introduced in the sub-library amplification step (Additional file 2: Table S1). We modified the original SPLiT-seq protocol to make it compatible with ACME-dissociated cells. First, we eliminated the formaldehyde fixation included in the original protocol as our cells are already fixed by ACME. Furthermore, we eliminated random hexamer RT oligos, as these could result in the excessive recovery of highly abundant cytoplasmic rRNA. In our configuration, we used 48 RT poly-dT barcodes and 96 ligation barcodes in each of 2 rounds of ligation, and 3 sub-libraries (Fig. 6a), which together generate 1.3 million possible barcode combinations. The barcodes are concatenated in the terminal part of the resulting cDNA sub-library, whereas the other end contains the mRNA sequence (Fig. 6a, Additional file 1: Figure S4B). To minimize collisions (cells receiving the same combination of barcodes), it is recommended to use less than 5% of the number of possible combinations. We started the experiment with ~ 480K cells (~ 10K per well). Since cells are lost throughout the barcoding process, only ~ 8% of the cells were detected in the last barcoding step, before cell lysis and sub-library generation. This level of cell loss is comparable to that reported in other combinatorial barcoding experiments [25]. We generated 3 sub-libraries from a total of ~ 40K ACME-dissociated cells from the two planarian species.

Our cDNA sub-libraries ranged between 400 and 1000 bp in length (Additional file 1: Figure S4C). We subjected these libraries to NovaSeq Illumina sequencing (paired 150 bp read length), with 425, 502, and 436 million (M) read pairs provided by the sequencing center. After removal of low-quality, truncated, and adapter chimera sequences, read depth was 173, 192, and 196 M read pairs. To analyze this experiment, we generated novel annotations (see the “Methods” section) of the most recent *S. mediterranea* genome assembly [60] and the recently sequenced *D. japonica* genome [61]. We mapped 96% and 85% of *S. mediterranea* and *D. japonica* reads, respectively, to gene models in their respective gene annotations. Despite obtaining a similar amount of reads mapping to the *D. japonica* genome, the genome annotation of this species is less complete, due to the fragmented assembly. We selected cells with reads mapping to ≥125 genes (19,975 and 14,263 cell barcodes in *S. mediterranea* and *D. japonica*, respectively). We also discarded very high UMI containing cell barcodes (> 5000 counts) to prevent the inclusion of aggregates that may have remained after FACS purification, as well as cells sharing the same cell barcodes through “collisions” (234 and 177 cell barcodes excluded in *S. mediterranea* and *D. japonica*, respectively). This rendered 19,741 cells for *S. mediterranea* and 14,086 cells for *D. japonica*. The latter species has a comparatively less complete genome annotation, and consequently, fewer cells are above the high gene coverage threshold at the same sequencing depth. Sub-library 3 contained fewer cells of both species, but those cells above thresholds in sub-library 3 were comparable to other libraries in UMI content (Additional file 1: Figure S4D) and had cells distributed throughout all clusters (Additional file 1: Figure S4E). Within these sets, after excluding high UMI cells, we obtain an average of 897.5 UMI per cell for *S. mediterranea* and 949.8 UMI for *D. japonica* (Fig. 6b). At this depth, we observe that libraries are not yet saturated, as shown by evaluating the number of UMIs and genes detected after performing subsampling at different fractions of the total depth (Fig. 6c). To estimate the presence of collisions, we took advantage of the species-mixing approach and we mapped reads to a combination of both genomes. We detected only 923 (2.8%) cell barcodes with mapping to both species above a stringent cutoff of 10% (Fig. 6d), showing the quality of our SPLiT-seq data.

### Cell type composition of two planarian species

**Fig. 7:**
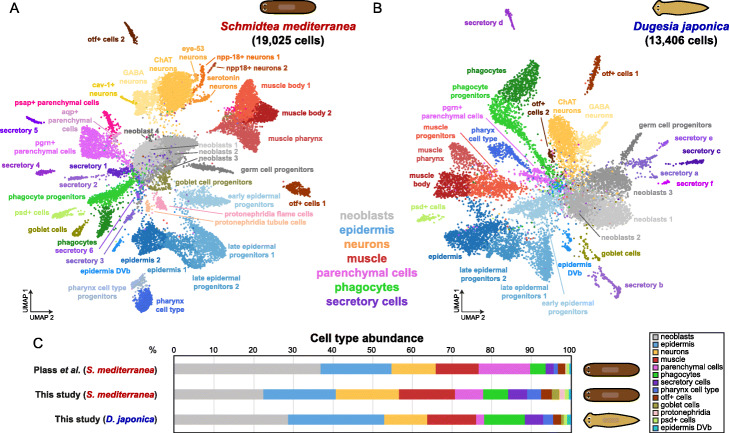
SPLiT-seq single-cell transcriptomic analysis of ACME-dissociated cells from two planarian species. **a**, **b** UMAP visualization of 19,025 *S. mediterranea* cells (**a**) and 13,406 *D. japonica* cells (**b**), colored by cluster identity and annotated on the basis of marker genes and homologous marker genes respectively. **c** Comparison of cell proportions for *S. mediterranea*, in comparison with a previous cell type atlas (Plass et al.) and *D. japonica*. Cell clusters are grouped by cell type group

To establish cell types and abundances in both species, we further analyzed our sets of 19,741 and 14,086 cells. To minimize the possibility of inclusion of *Schmidtea-Schmidtea* and *Dugesia-Dugesia* doublets, we used DoubletDecon [62], which resulted in further refined datasets of 19,025 and 13,406 cells, respectively. We found 41 cell clusters in *S. mediterranea* (Fig. 7a, Additional file 3: Table S2). Four central clusters highly expressed neoblast makers such as *smedwi-1* (Additional file 1: Figure S5). Within the remaining clusters, we found cells expressing markers (Additional file 4: Table S3) from all progenitor and differentiated cell types that we described previously using Drop-seq [13] (Additional file 1: Figure S5). Some groups were clustered together in fewer clusters (Additional file 1: Figure S5). For instance, we identified only 3 clusters containing parenchymal cell types, also termed cathepsin+ cells [12], but within them, we identify markers of all 7 clusters of parenchymal cells described in Plass et al. In other cases, such as the secretory cell types, we achieved better clustering (Additional file 1: Figure S5), finding 6 well-resolved cell clusters, compared to 4 in the trypsin-based dataset. Remarkably, we found a neoblast cluster containing *nanos-*positive germ cell progenitor cells that have not previously been observed in single-cell atlases (Additional file 1: Figure S6A). These cells are well described in the literature [63, 64], but none of the previous planarian single-cell transcriptomic studies [12, 13, 16] was able to distinguish them from the other neoblast populations (Additional file 1: Figure S6A). Though these germ cell progenitor cells are rare (1.6% of our total cell number), single-cell methods can detect far rarer cell types. Furthermore, previous studies included more cells and/or higher UMI contents. To rule out that this cluster arises as an artifact of aggregates, we monitored high UMI-containing cells in our clusters both before and after applying the > 5000 UMI cutoff (Additional file 1: Figure S7). In neither of these cases, the *nanos*-positive cluster is associated with high UMI-containing cells. Therefore, these facts cannot explain their clustering together with other neoblast populations in other studies. This strongly suggests that the detection of these germ cell progenitors in our study relies on the early fixation provided by ACME. We detected low abundance cell clusters described in Plass et al., including two protonephridia cell types (tubule and flame cells, 0.4 and 1.0%, respectively), psd+ cells (1.0%), epidermis of the dorsoventral boundary (DVb) (0.5%), and even lower abundance neuron subtypes including eye-53 expressing neurons (0.2%).

We then aimed to analyze for the first time the *D. japonica* cell type atlas. This planarian species belongs to another planarian clade, and its last common ancestor with *S. mediterranea* lived ~ 85 million years ago [65]. Due to its comparatively lower cell numbers, we were able to confidently detect fewer cell clusters. We annotated cell types by comparing their markers to their *S. mediterranea* homologs (Fig. 7b, Additional file 3: Table S2), finding similar cell types in comparable relative abundances. While it is difficult to establish one-to-one homology of cell types based on top markers [66], we confidently detected the major cell type groups with this approach (Additional file 4: Table S3): neoblasts, epidermis, neurons, muscle, parenchymal cells, phagocytes, and secretory cells, to a total of 28 cell clusters. At this resolution, we are only able to cluster two types of neurons and muscle respectively. We also identify low abundance cell types, such as psd+ cells (0.8%) and epidermis DVb cells (1.0%). Encouragingly, our analysis recovers the germ cell progenitors (1.5%) in this species as well (Additional file 1: Figure S3B).

We then compared the cell type compositions of both species. We grouped cell types in groups according to previous data [13]. We first compared the *S. mediterranea* dataset to our previously described dataset generated from trypsin dissociated cells analyzed by Drop-seq (Fig. 7c). Our *S. mediterranea* dataset contains ~ 22% neoblasts, in line with microscopy-based estimates [45], obtained by the classic maceration technique. The most abundant cell type groups in *S. mediterranea* are epidermal, neural, muscular, and parenchymal cells, present at comparable proportions as those described. This shows that ACME dissociation robustly retrieves all cell types at comparable proportions, not introducing biases in cell type composition, and can retrieve even lowly abundant cell types. We then compared the two species. The most abundant cell types in both species are stem cells, representing 20–35% of the total cell number. In both species, the most abundant differentiation cell type groups are epidermal, neurons, and muscle cells. Our *D. japonica* dataset contains considerably less parenchymal cells (1 cluster, 2.1% compared to 3 clusters with 7.1% of the total cells in *S. mediterranea*). These cell types were shown to vary with animal size [45] by microscopic observation. Future analyses, enabled by the characteristics of ACME dissociation and the multiplexing capacity of SPLiT-seq, will characterize the cell type composition of each species and the factors that underlie it. Furthermore, comparing the gene expression patterns of each cell cluster will provide insights into the cell type evolution. With regard to our methodology, this proof of principle highlights the flexibility and efficiency of ACME by allowing the robust simultaneous processing of two (and potentially more) species in a single SPLiT-seq and sequencing run.

### Integration of trypsin-dissociated and ACME-dissociated planarian single-cell datasets

**Fig. 8:**
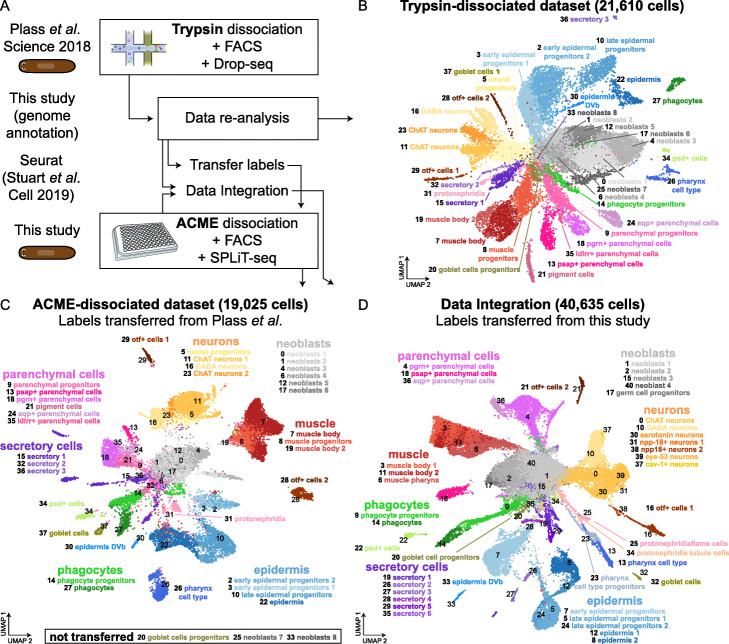
Integrative analysis of planarian trypsin- and ACME-dissociated datasets. **a** Analysis workflow. **b** UMAP visualization of 21,610 trypsin-dissociated *S. mediterranea* cells reanalyzed from Plass et al., colored by cluster identity and annotated on the basis of marker genes. **c** UMAP visualization of 19,025 ACME-dissociated *S. mediterranea* cells colored by transfer label analysis from trypsin-dissociated cell analysis (**a**). The numbers in the plot denote the center of the cells transferred with each of the trypsin-dissociated cell clusters. **d** UMAP visualization of 40,635 cells integrating trypsin-dissociated and ACME-dissociated cells. Clusters are colored by transfer label analysis from ACME-dissociated cell analysis

We then compared trypsin- and ACME-dissociated datasets to show that ACME-dissociated cells can be integrated with other datasets and to study the differences between the results of these types of dissociation. To do this, we selected the dataset generated by Plass et al. [13], as it is similar in cell numbers and depth, but was generated with trypsin-dissociated cells and Drop-seq [33]. We re-analyzed the data using our novel gene annotation (Fig. 8a). To compare UMI and gene per cell statistics in the two datasets, we performed downsampling experiments (Additional file 1: Figure S8). When both datasets are downsampled to 10K reads per cell, our ACME-dissociated dataset still recovers a median of 723 UMI per cell (compared to 594 in the Plass et al. dataset) and 176 genes per cell (compared to 394). This relatively lower gene recovery can be attributed to several causes, potentially including a comparatively higher recovery of intronic regions in the SPLiT-seq dataset (37.83% compared to 23.7% in the Plass et al. dataset, 55.7% if only *S. mediterranea* reads are considered) or differences in the presence of ambient RNA.

We then clustered the cells to obtain a similar number of clusters (38 in Plass et al. compared to 41 in our study). We annotated these clusters based on markers published in the original study (Fig. 8b, Additional file 3: Table S2). This resolution recovered almost all clusters obtained in the original publication. We then transferred the labels of cell clusters in this dataset to our ACME-generated dataset (Fig. 8b), resulting in the identification of equivalent cell types for the vast majority of Plass et al. cell types. To determine if the genes that are differentially expressed in these clusters are comparable in the two datasets, we performed a series of differential gene expression comparisons between neoblasts and a differentiated cell type in the two datasets (Additional file 1: Figure S9, Additional file 5: Table S4). These analyses showed a high overlap (74.3–84.3% depending on the cell type). We observed that clusters obtained in the ACME-dissociated dataset are more homogeneous in terms of UMI content (Additional file 1: Figure S10A) and number of genes detected (Additional file 1: Figure S10B), albeit this parameter is lower than in trypsin. Our dataset did not cluster out types such as the pigment cells, but we found these clustered together with a cluster from a related cell lineage (the pgrn+ parenchymal cells), as suggested by the presence of markers within those clusters (Additional file 1: Figure S5). Only 3 clusters from the Plass et al. dataset did not possess equivalents in our dataset: the two smallest neoblast clusters and the goblet cell progenitors. These clusters contain low cell numbers (0.4–1.6%). We therefore found equivalents for clusters representing ~ 97% of cells of the trypsin-generated dataset. When we performed the reverse comparison (Additional file 1: Figure S11A), a larger number of clusters from our dataset were not identified in the trypsin-generated dataset (Additional file 1: Figure S11B). These were also minor clusters (0.2–1.8%) and included neuron types such as the serotonin and eye-53+ neurons. This suggests that most differences in cell type clustering can be explained by cluster size and resolution parameters, and both datasets contain broadly comparable cell types. However, the germ cell progenitor cluster of our ACME-generated dataset was transferred to the neoblast clusters of Plass et al. (Additional file 1: Figure S11C). Similarly, markers of the germ cell progenitors were also found interspersed among the neoblast clusters in our analysis of the Plass et al. dataset (Additional file 1: Figure S11D). This supports the claim that the clustering of germ cell progenitors in our dataset depends on ACME dissociation, as none of the trypsin-based analyses shown here or previous papers retrieves them. While differences in clustering resolution parameters, cell numbers, and UMI content can explain these differences, altogether, our analysis shows that cell types obtained with trypsin and ACME dissociations are broadly comparable and that both methods robustly retrieve the major cell types of the planarian *S. mediterranea*.

We then wanted to test if the datasets could be integrated. Recently, algorithms to integrate single-cell datasets have been developed [67]. These rely on the identification of correspondent cells across the datasets (anchors). We successfully integrated both of these datasets (Fig. 8c), further demonstrating the compatibility of both dataset types. We transferred labels from this study (Fig. 8c) and also from the trypsin-generated dataset of Plass et al. (Additional file 1: Figure S11E), resulting in both cases in complete transferring of labels to the integrated set. This dataset contains 40,635 cells and will serve as a comprehensive resource for future studies of *Schmidtea mediterranea*. Altogether, both transfer labels and integration comparisons show that trypsin-dissociated and ACME-dissociated single-cell transcriptomic datasets are broadly compatible and can be integrated in a straightforward manner. This further shows that ACME dissociation of cells is a robust method for studying the biology of cell types and retrieving cell type atlases by single-cell transcriptomics.

## Discussion

Here, we present ACME dissociation, a new cell dissociation protocol for single-cell transcriptomics. Our protocol relies on the principle of acetic acid-methanol dissociation, an approach used to dissociate cells for microscopy in past centuries but not yet applied to modern single-cell transcriptomics. The original maceration protocol was applied to relatively soft-bodied animals such as planarians and cnidarians. However, we have shown that the approach works, with slight modifications, in a broad range of animals with hard body parts such as chorions, vitelline membranes, cuticles, and shells. ACME cannot dissolve or penetrate these hard parts, but straightforward mechanical disruption is sufficient to extract cells from their acellular surroundings. We have successfully obtained dissociated cells from species belonging to all major animal groups from a wide range of habitats. It is possible that further modifications of the ACME protocol will specifically optimize the quality of cell suspensions in different organisms. We highlight the main criteria for species-specific optimization (time of dissociation, mechanical disruption, filtering steps), but other changes might help provide the ideal dissociation conditions for each organism. Our protocol provides a robust and broadly applicable starting point for such optimization. We also note that while ACME provides simultaneous dissociation and fixation, it is also worthwhile to consider ACME as a fixative of cells dissociated with other methods, as it preserves RNAs with high integrity, is compatible with cell staining and FACS, and provides an excellent platform for both droplet-based and combinatorial methods of single-cell transcriptomics.

ACME is a cell dissociation approach that fixes cells while they are being dissociated. This has enormous advantages over enzymatic and mechanical approaches, as disrupting the cellular environment in live cells has effects on the cellular transcriptomic profiles that are only beginning to be realized [38, 39, 68]. Unlike nucleus extraction approaches, ACME preserves the cell cytoplasm, where most cellular mRNAs reside. Furthermore, the dissociation-fixation approach streamlines the preparation of cell suspensions for single-cell transcriptomics, with the possibility of cryopreserving cells before and/or after the FACS sorting. This will allow a range of experiments presently beyond the range of current approaches, including the collection of large sample sets, consisting of different treatments, time points, and/or replicates. These can then be subject to simultaneous or sequential cryopreservation, and single-cell transcriptomics can be performed on all samples, multiplexed together in a single SPLiT-seq run. This represents a marked improvement on current workflows, helping prevent a variety of batch effects from accumulating. For instance, this could lead to clinicians preserving dissociated patient material to be later subjected to single-cell transcriptomics at a different research institution.

Similarly, enzymatic methods are hard to apply to organisms that are difficult to culture in the laboratory, as single-cell transcriptomics, or at least FACS and cryopreservation, needs to take place immediately when using previous protocols. ACME dissociation consists of simple reagents and only requires widely available instruments: a shaker, a low-speed centrifuge, and a freezer. Cells are fixed from the beginning of the process and can be immediately cryopreserved. Therefore, we envision that ACME dissociation will facilitate the single-cell analysis of organisms from locations where single-cell facilities are not available, such as on field sampling trips, and allow exchange between collaborating institutions. This will accelerate our knowledge of cell types across the tree of life.

We have demonstrated that ACME-dissociated cells can be subjected to single-cell sequencing using several platforms. In particular, we have used the commercial droplet-based method 10X Genomics and a combinatorial barcoding approach, SPLiT-seq. Even at low sequencing depths, and with relatively low UMI counts per cell, we identify cells of all cell types that were previously described by enzymatic dissociations analyzed by MARS-seq in the cnidarian *N. vectensis* [10] and Drop-seq [13] in the planarian *S. mediterranea* and found a similar number of cell clusters in a previously uncharacterized planarian species, *D. japonica*. This shows that the “many cells, few UMIs per cell” approach is highly effective to profile cell types, as previously suggested [25], even in uncharacterized species.

As ACME-dissociated cells are fixed and can easily be cryopreserved, we also envision that ACME could provide a framework for developing complex labeling procedures for single-cell analysis. Our current procedure involves one step of RT and two rounds of splint oligo ligation, after DNA and cytoplasm labeling. We foresee that more complex staining procedures (such as immunohistochemistry or mRNA in situ hybridization, or several metabolic labeling procedures) could be used to subset cells that would be later sorted by FACS to profile lowly abundant cell populations.

## Conclusions

We show that ACME is a versatile and powerful cell dissociation method for single-cell transcriptomics. ACME dissociation provides a solution to various shortcomings of the canonical single-cell transcriptomic workflow, providing early fixation of material. This fixation process provides opportunities for the further development of this technique. Therefore, we believe that ACME will be a valuable tool for single-cell transcriptomics that will greatly enable the investigation of cell type diversity and dynamics in multiple different organisms presently beyond the scope of current techniques in this revolutionary approach.

## Methods

### ACME dissociation

ACME solution was prepared fresh using a 13:3:2:2 ratio of commercially sourced DNase/RNase-free distilled water, methanol, glacial acetic acid, and glycerol. For each sample, between 10 and 30 mixed-size adult planarians (cultured as previously described [13]) were added to a 15-mL Falcon tube, for a final biomass volume of ~ 100–300 μL. We removed planarian water using a Pasteur pipette and added ~ 100–500 μL of 7.5% *N*-acetyl cysteine in 1× PBS, sufficient to cover the planarians. *N*-acetyl cysteine helps clean planarian mucus and protects RNA. The ACME solution was immediately added to samples to a final volume of 10 mL per tube. Alternatively, *N*-acetyl cysteine can be added to the ACME solution at this stage, in the same quantity as noted above.

Samples were left to dissociate at room temperature for 1 h on a see-saw motion shaker at 35–45 rpm, with tubes oriented parallel to the direction of movement. We then pipetted the reactions up and down several times to complete dissociation using 1-mL pipette tips. From this point, samples were kept on ice to prevent RNA degradation. We centrifuged samples at 1000*g* for 5 min (4 °C) to remove the ACME solution. The resulting pellet may not be completely compact, so the supernatant must be discarded carefully. To clean the cells, 7 mL of buffer (1× PBS 1% BSA) was added and the pellet was mixed by flicking. Samples were centrifuged again at 1000*g* for 5 min (4 °C), and the supernatant was removed. If the pellet was still not compact, an additional cleaning step was performed to remove the remaining ACME solution. Pellets were resuspended in 900 μL of buffer (1× PBS 1% BSA) and transferred to 1.5-mL Eppendorf tubes.

To cryopreserve cells, we added 100 μL of DMSO per tube [48] and stored the samples directly at − 80 °C. Afterwards, we thawed the samples on ice and centrifuged them at 1000*g* for 5 min (4 °C) to remove DMSO. The supernatant was discarded and pellets resuspended in 1 mL of washing buffer (1× PBS 1% BSA). Samples were centrifuged again, and pellets were resuspended in 1 mL of fresh washing buffer.

ACME dissociation in other animals was performed with modifications to the above protocol. Zebrafish embryos were dechorionated before the ACME solution was added, and mechanically disrupted in the ACME solution by applying short pulses of Polytron homogenization. *Lymnaea stagnalis* embryos were decapsulated by passing them through a syringe and then mechanically disrupted in the ACME solution. To remove broken eggshells, the cell dissociation mixes for both these species were passed through a 100-μm CellTrics filter (Sysmex). *Parasteatoda tepidariorum* egg capsules were either manually dissected under the scope within the ACME solution, or mechanically disrupted in the ACME solution using short pulses of Polytron homogenization, and filtered through a 40-μm cell strainer (Corning). *Pristina leidyi* adults were manually shaken every 10 min during dissociation.

### Protecting RNA from degradation

In the course of ACME dissociation, RNAs are exposed to hydrolysis or degradation by RNAses. Therefore, RNAse-free conditions are essential. We have found that *N*-acetyl-l-cysteine (NAC, Sigma A7250) results in better RNA integrity after maceration. NAC was initially added as a mucolytic agent to planarian ACME dissociations. NAC is a reducing agent that breaks up disulfide bonds in the mucus, solubilizing it. NAC is also widely used due to its antioxidant properties [49]. NAC is acidic in solution and can be mixed with the acidic ACME solution, resulting in reducing, RNA protective, conditions. We first used NAC as an initial wash step. Due to its acidity, this step has to be performed quickly as it results in cell dissociation and lysis. We also have had good results mixing the NAC directly with the ACME solution. The concentration of NAC in the ACME solution and the possibility of performing it as an initial wash step should be evaluated in each case.

We have also found that a significant source of RNAse contamination can come from the use of BSA to avoid cell clumps. While RNAse-free BSA can be obtained commercially, it is often too expensive to be used in large amounts. However, we have had good results with non-RNAse-free BSA Microbiological Grade Powder (Thermo Fisher, cat. BP9700100). We recommend testing the RNAse activity in advance and making aliquots to prevent cross-contamination and bacterial growth.

### Trypsin dissociation

For these experiments, between 50 and 70 mixed-size adult planarians (*S. mediterranea*) were chopped into small pieces (< 1 mm) in a petri dish with a sterile, new razor blade. With a Pasteur pipette, chopped worms were transferred to a 15-mL Falcon tube containing 10 mL of 1% trypsin, diluted in 1× PBS. The sample was incubated for 30 min at room temperature in a see-saw shaker at 35–45 rpm, with tubes oriented parallel to the direction of movement. The reaction was pipetted up and down several times every 10 min to help dissociation. After incubation, we filled the Falcon tube to 14 mL with buffer (1× PBS 1% BSA) and centrifuged at 1000*g* for 5 min (4°). We discarded the supernatant and resuspended the pellet in 5–10 mL of buffer (1× PBS 1% BSA). Cells were then passed through a 50-μm CellTrics filter (Sysmex) and, subsequently, passed through a 20-μm Nylon net filter (Millipore) into a new 15-mL Falcon tube. We filled the tube to 14 mL with buffer and centrifuged at 1000*g* for 5 min (4 °C). The resulting pellet was resuspended in 1–2 mL of buffer (1× PBS 1% BSA) and transferred to 1.5-mL Eppendorf tubes. Trypsinized cells were kept on ice to avoid cell death. These were then fixed, using either ACME or formaldehyde, or directly stained for flow cytometry visualization.

### ACME fixation after trypsin dissociation

As an alternative to ACME dissociation, tissues can be dissociated using trypsin (as described above), and cells are then fixed and permeabilized with ACME. For this procedure, we began with 300–600 μL of trypsinized cells per sample in a 15-mL Falcon tube. We added 8.5 mL of ACME solution without methanol: 6.5 mL of buffer (1× PBS 1% BSA), 1 mL glycerol, 1 mL acetic acid, and 100 μL of 7.5% NAC. Note that in this case, we used PBS buffer for the ACME solution, instead of distilled water, to avoid an osmotic shock to dissociated cells. We incubated this mix for 15–20 min at room temperature in a see-saw shaker at 35–45 rpm. Subsequently, we added 1.5 mL of methanol and incubated for another 15–20 min. Methanol was added at a later stage to allow partial fixation in acetic acid prior to permeabilization. In general, the variations in this protocol, compared to ACME dissociation, are focused on protecting previously dissociated cells, as they are more fragile than whole tissues. Afterwards, we centrifuged the samples at 1000*g* for 5 min (4 °C) to remove the ACME solution. We discarded the supernatant carefully, as the pellet may not be compact, and resuspended it in 7 mL of buffer (1× PBS 1% BSA). Cells should resuspend without additional mixing. Samples were centrifuged again at 1000*g* for 5 min (4 °C), resuspended in 1 mL of buffer (1× PBS 1% BSA), and transferred to 1.5-mL Eppendorf tubes. From this point, cells were treated as regular ACME-dissociated cells and can be cryopreserved or visualized by flow cytometry in the same way.

### Formaldehyde fixation

For each fixation, we took 100–200 μL of previously trypsinized cells and resuspended them in 8 mL of buffer (1× PBS 1% BSA) in a 15-mL Falcon tube. We added 2 mL of previously prepared formaldehyde (FA) stock diluted in 1× PBS (at 0.5%, 2.5%, 5%, 10%, or 20% FA in PBS), to result in a final concentration of 0.1%, 0.5%, 1%, 2%, or 4% in 10 mL total volume. The Falcon tubes were immediately gently shaken to homogenize the FA concentration. Samples were then incubated for 10 min at 4 °C in a see-saw shaker at 35–45 rpm. We then centrifuged twice at 1000*g* for 5 min (4 °C) to remove FA. After each centrifugation step, the supernatant was discarded. Fixed cells were resuspended in 5–7 mL of buffer (1× PBS 1% BSA), after the first centrifugation, and in 1 mL of TRIzol after the second, for RNA extraction as described below.

### RNA quality assessment and extraction

For the assessment of time and temperature-dependent RNA degradation (Fig. 3h, i), for each experiment, we prepared planarian samples (*S. mediterranea*) as described in the ACME dissociation section above.

For assessment of temperature-dependent RNA degradation after ACME dissociation (Fig. 3i), after one freezing cycle, two samples were pooled together to avoid sample-specific effects, and cells were resuspended in 4 mL of buffer (1× PBS 1% BSA). Then, cells were split again into two different 2-mL Eppendorf tubes. One of the tubes was left at room temperature, and the other was kept on ice for 6 h. We took the samples from each tube at sequential time points (0 h, 1 h, 2 h, 3 h, 4 h, 5 h, and 6 h) for RNA extraction (see the method below).

To evaluate the RNA integrity along our pipeline (Fig. 3j), we extracted RNA from different ACME-dissociated samples right after dissociation (b), after freezing (c), after FACS sorting (d), and after sorting and freezing (e). To evaluate RNA resistance to freezing (Fig. 3k), we froze and thawed the same ACME dissociation for 5 cycles, taking a sample for RNA extraction after each cycle. Untreated whole-tissue RNA extractions were used as positive controls.

For comparison of RNA integrity between ACME-fixed vs formaldehyde-fixed cells (Fig. 4), we made a single preparation of trypsin-dissociated cells (as described above), and divided it into seven different subsamples: a trypsin-dissociated sample which was directly extracted in TRIzol, a sample that was ACME-fixed (see ACME fixation protocol) and 5 formaldehyde-fixed samples, using different FA concentrations (0.1%, 0.5%, 1%, 2%, and 4%), generated according to the formaldehyde fixation protocol above. This experiment was performed in 3 replicates.

All RNA extractions were performed using TRIzol or TRIzol LS, following the manufacturer’s protocol. RNA quality was assessed using an Agilent 2100 Bioanalyzer, according to the Agilent RNA 6000 Nano Kit Guide.

### Flow cytometry and FACS

ACME-dissociated and ACME-fixed cells were filtered through 50-μm CellTrics strainers (Sysmex), collected in 1.5-mL Eppendorf tubes and stained with the nuclear dye DRAQ5 (eBioscience) adding 1–8 μL/mL of 5 mM stock solution (we normally use 1 μL/mL for planarian samples), and the cytoplasmic dye Concanavalin-A conjugated with AlexaFluor 488 (Invitrogen), adding 2 μL/mL of 1 mg/mL stock solution. Trypsin-dissociated cells were filtered through a 50-μm CellTrics filter (Sysmex) and a 20-μm Nylon net filter (Millipore) as previously described. For flow cytometry visualization, trypsin-dissociated cells were stained with DRAQ5 as a nuclear dye, adding 10 μL/mL of 5 mM stock solution, and Calcein (eBioscience) as a live cell labeling dye, adding 1 μL/mL of a 0.5 mg/mL stock solution.

Staining concentrations require optimization and ultimately depend on FACS/Cytometer adjustments and cell concentration. Cells were stained in the dark, on ice, for 30–45 min, and visualized using a CytoFlex S Flow Cytometer (Beckman Coulter) or sorted using a BD FACS Aria III (BD Biosciences) Cell Sorter. To avoid RNAse contamination during cell sorting, the FACS was thoroughly decontaminated with bleach and pre-cooled before sorting, keeping injection and collection chambers at 4 °C during the process. Sorting was performed using the BD FACSDiva Software, setup in 4-Way Purity mode, with an 85-μm nozzle and moderate-pressure separation (45 Psi). We set DRAQ5-positive, Concanavalin-A-positive single-cells to be sorted and collected in 1.5-mL Eppendorf tubes with 100 μL of collection buffer (1× PBS, 1% BSA), obtaining up to 500,000 cells per tube. Completing a sorting run normally takes 3 to 5 h. After sorting, samples were centrifuged at 1000*g* for 5 min (4 °C). The supernatant was removed, and the pellet resuspended in 900 μL of fresh buffer (1× PBS 1% BSA). We cryopreserved the cells at this point, by adding 100 μL of DMSO and storing them at − 80 °C.

### Irradiation

We irradiated three petri dishes with 20 planarians each at 60 Gy in a Gamma (Cs-137) Cabinet Irradiator. As a negative control, we used three equivalent non-irradiated samples. Irradiated planarians and controls were dissociated 72 h post-treatment according to the ACME dissociation protocol described above. After dissociation, samples were resuspended in 1 mL of buffer (PBS 1× BSA 1%) and passed through a 50-μm CellTrics filter (Sysmex). Samples were then stained with 2 μL of DRAQ5 (5 mM) and 2 μL of Concanavalin-A (1 mg/mL) and profiled by flow cytometry.

#### scRNA-seq of *Nematostella vectensis* ACME-dissociated cells using 10X Genomics platform

For *Nematostella vectensis* ACME dissociations, 10 juveniles were quickly washed in cold 7.5% NAC in PBS before the addition of 4 mL of ACME solution. The tissue in ACME solution was transferred into a gentleMACS C-tube (Miltenyi Biotech, 130-093-237) and incubated in a rocking table for 10 min before running the program “B” on the gentleMACS octo dissociator (Miltenyi Biotech). The process was repeated 3 times using the program “Multi_A_1” for a total time of 1 h in the ACME solution at RT. Cells were then transferred to a conical tube, centrifuged at 1500*g* at 4 C, resuspended in PBS 0.5% BSA in the presence of RNAse inhibitor (40 U/mL), and frozen after the addition of 10% DMSO. After this, cells were thawed, washed in PBS 0.5% BSA in the presence of RNAse inhibitor, stained with 0.33 μL/mL of DRAQ5 (stock 5 mM), filtered through a 40-μm strainer, and sorted using a BD Influx cell sorter. A total of 6250 singlets with 2n DNA content (G1/G0) were directly sorted into Master Mix without RT enzyme C (10x Chromium single cell 3′ reagents kit v3.1), using 1-Drop purity mode, a 100-μm nozzle and 20Psi (Additional file 1: Figure S3A). After adding the RT enzyme C, cells were immediately loaded onto a 10x Genomics Chromium chip and single cell 3′ GE libraries (v3.1) constructed according to the manufacturer’s protocol. cDNA was amplified 12 cycles, and the sample was PCR indexed 16 cycles. The library was sequenced on a HiSeq 2500 with 50 PE.

### Cnidarian single-cell transcriptomic analysis

We used Metacell [69] to select gene features, to construct cell clusters (termed metacells) used in the downstream analyses, and to generate projected data visualizations. We first filtered out cells with too low (< 100) or too high (> 10,000) a number of UMIs. We selected feature genes using a normalized size correlation threshold of − 0.05 and normalized niche score threshold of 0.05, with > 1 UMI in at least three cells and a total UMI count > 30 molecules. For kNN graph building, we used *K* = 100 as the target number of edges per cell, and for metacell construction, we used *K* = 30, minimum module size of 20, and 1000 iterations of bootstrapping with resampling of 75% of the cells. This way, we obtained a robust estimate of co-clustering between all pairs of single cells and identified clusters of single or grouped metacells. Lastly, we filtered out low-quality metacells expressing less than 30 marker genes and with total UMI count falling below UMI distribution peak, unless they specifically expressed more than two TF genes (log fold change > 2). To perform label transfer, for every single cell in the ACME dataset (query), we calculated the top Pearson correlation to any cell in the fresh dataset [10] using functions implemented in Metacell. Briefly, the average top *k* correlations were used to transfer labels to single cells and metacells in the query dataset, where *k* is the maximum number of edges in the cell-to-cell similarity graph in the reference dataset. We assigned labels to query metacells for which averaged top correlations to the reference dataset were higher than the average correlations to cells in the query dataset (absolute difference > 1). We generated single-cell gene expression heatmaps using the R package ComplexHeatmap [70], showing the top 20 genes with log fold change > 2 per metacell and highlighting TFs.

### Split pool ligation-based transcriptome sequencing

The SPLiT-seq protocol was performed as previously described [34] with some modifications. Different samples of sorted ACME-dissociated planarian cells, from *S. mediterranea* and *D. japonica*, were thawed, centrifuged twice at 1000*g* for 5 min (4 °C) to remove the DMSO, and resuspended in 100–200 μL of buffer (1× PBS 1% BSA). For each sample, we stained 100 μL of a 1:10 dilution for 15–20 min and counted these subsamples by flow cytometry. The remaining portion of cells was then diluted and pooled together (mixing both species cells) to a final concentration of 1.25 M cells/mL (10,000 cells per well for the reverse transcription round: 5000 cells per each planarian species).

#### Plate preparation

Barcodes were provided lyophilized by integrated DNA technologies on three 96-well stock plates: Stock-1 (well-specific anchored poly (dT) *Round 1 barcodes), Stock-2 (well-specific *Round 2 barcodes), and Stock-3 (well-specific *Round 3 barcodes). Lyophilized barcodes were resuspended in DNAse/RNAse-free water to a final concentration of 100 μM/well. From the stock plates, we prepared another three plates at specific working dilutions (WD-1, WD-2, and WD-3 from stock plates 1–3, respectively). Using a multichannel pipette, we prepared WD-1 with 12 μL of round 1 barcodes from Stock-1 and 88 μL of DNAse/RNAse-free water per well. For WD-2, we mixed 12 μL of round 2 barcodes from Stock-2, 11 μL of *Linker_1 (100 μM) and 77 μL of DNAse/RNAse-free water per well. Finally, WD-3 was made of 14 μL of round 3 barcodes from Stock-3, 13 μL of *Linker_2 (100 μM) and 73 μL of DNAse/RNAse-free water per well.

With a total volume of 100 μL per well, WD-1 will last for up to 25 experiments (4 μL/well used per experiment), while WD-2 and WD-3 plates will last for up to 10 experiments (10 μL/well used per experiment). Before following the SPLiT-seq protocol, WD-2 and WD-3 were heated to 95 °C, for 2 min, and ramped down to 20 °C at a rate of − 0.1 °C/s, to anneal the 5′ end of each barcode oligo to the universal linker oligos.

#### Round 1 of barcoding: reverse transcription

The first round of barcoding was carried out by in-cell reverse transcription (RT). The original SPLiT-seq protocol uses a combination of random hexamers and anchored poly (dT) oligos [34]. We only used the latter (Additional file 2: Table S1). A 96-well plate (round 1) was prepared on ice by transferring 4 μL/well of round 1 barcodes from WD-1. We then added 8 μL/well of RT mix “plate round 1”: 4 μL of 5× RT Buffer (Thermo Scientific), 0.35 μL of SUPERase-In RNAse inhibitor (20 U/μL, Invitrogen), 1 μL of 10 mM/each dNTPs (NEB), 1.65 μL of nuclease-free water, and 1 μL of Maxima H Minus RT (Thermo Scientific). Finally, 8 μL of previously counted cells (1.25 M cells/mL) was also added to each well, giving a total volume of 20 μL/well. Round 1 was incubated in a thermocycler for 30 min at 50 °C and immediately placed on ice. Individual reactions were then pooled together in a 15-mL Falcon tube, on ice, and round 1 plate was discarded. We added 9.6 μL of 10% Triton X-100 to the pooled cells (0.1% final concentration) and centrifuged them at 1000*g* for 5 min (4 °C). We discarded the supernatant and resuspended the pellet in 2 mL of 1× NEB buffer 3.1 (NEB).

#### Round 2 of barcoding: ligation 1

The second round of barcoding was carried out by a ligation reaction. A new 96-well plate (round 2) was prepared on ice with 10 μL/well of round 2 barcodes from WD-2. Then, 2 mL of ligation mix (500 μL of T4 Ligase Buffer 10× (NEB), 100 μL of T4 DNA ligase (400 U/μL, NEB), and 1500 μL of nuclease-free water) were added to the cells resuspended in 1× NEB buffer 3.1 and mixed thoroughly into a disposable basin. We added 40 μL/well of this ligation mix (including cells) to the round 2 plate and covered it with an adhesive PCR plate seal. The plate was incubated in a thermocycler for 30 min at 37 °C. To block Linker_1 after incubation, a blocking solution was prepared with 264 μL of *Blocker_1 (26.4 μM final concentration), 250 μL of T4 Ligase Buffer 10× (NEB), and 486 μL of nuclease-free water. After incubation, the seal was removed from the round 2 plate and 10 μL of blocking solution was added to each well, giving a final volume of 60 μL/well. The plate was sealed again and incubated for another 30 min at 37 °C.

#### Round 3 of barcoding: ligation 2

The third round of barcoding was carried out by a second ligation reaction. We filled a 96-well plate (round 3) with 10 μL/well of round 3 barcodes from WD-3. Then, we took round 2 from the incubator, pooled cells together into a new disposable basin, and discarded the plate. We added 100 μL of T4 DNA ligase (400 U/μL, NEB) to the basin and mixed thoroughly with the cells. The round 3 plate was then filled with 50 μL/well of this cell-ligase solution, sealed with a PCR adhesive and incubated for 30 min at 37 °C. Termination solution was prepared with 288 μL of *Blocker_2 (11.5 μM final concentration), 625 μL of 0.5 M EDTA (to stop ligase activity), and 1587 μL of nuclease-free water. We added 20 μL/well of the termination solution (for a final volume of 70 μL/well) without further incubation. Afterwards, cells were pooled into a 15-mL Falcon tube and placed on ice.

#### Cell lysis

Following the addition of 70 μL of 10% Triton-X 100 (0.1% final concentration), the pooled cells were centrifuged at 1000*g* for 5 min (4 °C). We carefully removed the supernatant (leaving about 100 μL) and resuspended the cells in 4.04 mL of washing buffer (4 mL of 1× PBS and 40 μL of 10% Triton X-100). The cells were centrifuged again at 1000*g* for 5 min (4 °C). After removing the supernatant, we resuspended the cells in 50 μL of 1× PBS buffer. We diluted 5 μL of this resuspension in 195 μL of 1× PBS and counted the number of cells by flow cytometry to decide the number of sub-libraries. Then, we aliquoted the remaining 45 μL of cells in 1.5-mL Eppendorf tubes according to the cell concentration obtained by flow cytometry. For this experiment, we generated three different sub-libraries of ~ 15.000 cells/each. The volume of each sub-library was adjusted to 50 μL with 1× PBS.

Lysis buffer was prepared with the following reagents (final concentrations in brackets): Tris pH 8.0 (20 mM), NaCl (400 mM), EDTA pH 8.0 (100 mM), and SDS (4.4%). We added 50 μL of lysis buffer and 10 μL of Proteinase K (20 mg/mL) to each sub-library and incubated the lysates at 55 °C for 2 h, shaking the tube manually every 15 min. After incubations, lysates were frozen at − 80 °C.

#### cDNA purification with magnetic beads

We used 44 μL of Dynabeads™ MyOne™ Streptavidin C1 (Invitrogen) per lysate to purify the cDNA by linking the beads to the biotin molecule at the 3′ end of the third barcode. We followed the manufacturer’s protocol for Dynabeads nucleic acid purification, with modifications taken from Rosenberg et al.’s protocol: Manufacturer’s Washing Buffer (1× B&W) was prepared with the addition of 0.05% final concentration of Tween-20. Lysates were incubated for 10 min at room temperature with 5 μL of 100 μM PMSF (diluted in isopropanol) to inhibit Proteinase K activity. Afterwards, lysates were incubated with the magnetic beads for 60 min, at room temperature, with rotation. During washing steps, samples were agitated in 1× B&W/Tween-20 buffer for 5 min at room temperature. Finally, beads were resuspended in 250 μL of 10 mM Tris-T buffer (10 mM Tris-HCl pH 8.0, 0.1% Tween-20 and 0.2% SUPERase-In RNAse Inhibitor) and kept at 4 °C.

#### Template switch

Template switch mix was prepared with 44 μL of 5× RT Buffer (Thermo Scientific), 44 μL of 20% Ficoll PM 400 (Sigma Aldrich), 22 μL of 10 mM/each of four dNTPs (NEB), 5.5 μL of *TSO primer (100 μM), 11 μL of Maxima H Minus RT, and 93.5 μL of nuclease-free water per sample. Dynabeads linked to cDNA were washed, using a magnetic rack, with 250 μL of nuclease-free water (no resuspension) and resuspended in 200 μL of the template switch mix. Samples were incubated in the mix for 30 min at room temperature and then for 90 min at 42 °C, with agitation. After incubation, the template switch mix was removed using a magnetic rack; beads were resuspended in 250 μL of Tris-T buffer and kept at 4 °C.

#### PCR amplification

The PCR mix was prepared with 110 μL of 2× Kapa HiFi HotStart ReadyMix (Roche), 8.8 μL of *PCR_PF (10 μM), 8.8 μL of *PCR_PR (10 μM), and 92.4 μL of nuclease-free water. Dynabeads in Tris-T buffer were placed in a magnetic rack and washed with 250 μL of water (no resuspension). Each sub-library was then resuspended in 220 μL of PCR mix and split into 4 PCR tubes. The following program was run in the thermocycler: 95 °C (3 min) and five cycles at 98 °C (20 s), 65 °C (45 s), and 72 °C (3 min). The 4 PCR reactions were combined again in a 1.5-mL Eppendorf, and Dynabeads were separated using a magnetic rack. Two hundred microliters of supernatant, containing cDNA in suspension, was split into 4 wells in a qPCR plate (50 μL/well). We added 2.5 μL of 20× EvaGreen (Biotium) to each well and run the following program in a qPCR thermocycler: 95 °C (3 min), cycling until plateau phase, normally 8–10 cycles, at 98 °C (20 s), 65 °C (20 s) and 72 °C (3 min), and a final elongation at 72 °C (5 min).

#### Size selection

We purified qPCR reactions by SPRI size selection to remove fragments smaller than 300 bp. We used Kapa Pure Beads (Roche) at a ratio of 0.8× and followed the manufacturer’s protocol for “Cleanup of Fragmented DNA in NGS Workflows,” with two modifications taken from the original SPLiT-seq protocol: washing steps were performed with 750 μL of 85% ethanol, and cDNA was eluted in 20 μL of nuclease-free water at 37 °C for 10 min.

#### Tagmentation

The sub-libraries were tagmented using the Nextera DNA Library Preparation Kit (Illumina). After the SPRI 0.8x size selection, we quantified the sub-libraries by Qubit (Thermo Fisher) and diluted 50 ng of cDNA in a total volume of 20 μL of nuclease-free water. The tagmentation reaction mix was prepared with 20 μL of cDNA (50 ng), 25 μL of Tagmentation Buffer, and 5 μL of enzyme 1. Samples were incubated in a pre-heated thermocycler for 5 min at 55 °C and placed on ice at the end of this time period. We neutralized the tagmentation activity of enzyme 1 by immediately cleaning the reaction with the Monarch PCR & DNA Cleanup Kit (NEB). Samples were eluted in a final volume of 20 μL of UltraPure water.

#### Round 4 of barcoding: PCR

The fourth barcode was introduced by PCR. We prepared a separate reaction mix for each sub-library, containing 20 μL of tagmented cDNA, 25 μL of 2× Kapa HiFi HotStart ReadyMix (Roche), 1.5 μL of *P5_oligo (10 μM), 1.5 μL of *Round 4 barcode (10 μM), and 2.5 μL of 20× EvaGreen (Biotium). For the three sub-libraries made in the present study, we used the *Round4_1, *Round4_2, and *Round4_3 oligos as round 4 Barcodes. The qPCR program ran as follows: 95 °C (30 s); cycling until plateau phase (8–10 cycles) at 95 °C (10 s), 55 °C (30 s), and 72 °C (30 s); and final elongation at 72 °C (5 min). The resulting qPCR reactions were size selected (SPRI 0.7x) by mixing 40 μL of the sample with 28 μL of Kappa Pure Beads (Roche), following the protocol described above. Each sub-library was resuspended in a final volume of 20 μL in nuclease-free water and fragment distribution was checked in an Agilent 2100 bioanalyzer following the Agilent High Sensitivity DNA Kit Guide.

*Oligo sequences provided in Additional file 2: Table S1.

### Planarian single-cell transcriptomic analysis

#### Sequencing and quality control

The three sub-libraries were pooled together and sequenced on a NovaSeq 6000 platform (Illumina) by Novogene, with 150 bp length, paired-end reads. These reads were provided without any quality verification except a basic chastity check. They were therefore subject to initial quality checks with FastQC. Read Phred quality was generally good, but adaptor and N content required curation and removal. CutAdapt v2.8 [71] was used to trim residual adaptor sequence, low-quality, and short reads. Differing strategies for clean-up were used for read 1 (transcript sequence) and read 2 (UMI and barcode sequences). For read 1, cutadapt -j 4 -m 60 -q 10 -b AGATCGGAAGAG was run, removing residual Illumina universal adapter and a read length shorter than 60 bp. For read 2, cutadapt -j 4 -m 94 --trim-n -q 10 -b CTGTCTCTTATA was run, removing reads shorter than 94 bp (the minimum to span all barcodes), terminal Ns, and residual Nextera adapter sequence. Read 2 sequences were checked for “phase” (i.e., whether barcodes were in their correct position, due to possible indels) using grep to compare adapter-derived flanking sequence was correctly positioned with that of each read. Reads were conservatively retained, with only reads with UMI and UBC barcodes in the correct location carried forward to further analysis. Dephasing, while advisable, did not prove a major issue, and very few reads were discarded. Makepairs (https://github.com/sestaton/Pairfq/wiki/makepairs) was used to retain only paired reads, and a further round of FastQC analysis was used to confirm all detectable adaptor and low-quality sequence had been removed.

#### Read mapping, barcode extraction, and matrix production

The *S. mediterranea* S2F2 genome [60] was downloaded from Planmine, and the *D. japonica* v 1.0 genome [61] was downloaded from http://www.planarian.jp. De novo gene models were created for both *S. mediterranea* and *D. japonica*. A total of 183 published *S. mediterranea* and 43 *D. japonica* RNA-seq datasets were downloaded from the NCBI SRA and the DNA Data Bank of Japan, comprising all those listed at the time of analysis. These collected reads were aligned to the respective reference genomes using HiSat 2.1.0 [72]. StringTie and StringTie—merge [73] were then used to merge mapping outputs with the existing SMESG-high confidence gene models from Planmine (*S. mediterranea*) and the full v1 AUGUSTUS-derived gene models from http://www.planarian.jp (*D. japonica*). Isoformal variants whose length was greater than 100 kb were removed from the gene set as likely artifacts (588 in *D. japonica*, 617 in *S. mediterranea*). *D. japonica* and *S. mediterranea* fasta and gtf files were then concatenated to create a combined database for mapping. Drop-seq_tools-2.3.0 (https://github.com/broadinstitute/Drop-seq) was then used to create sequence dictionary, refFlat, reduced GTF, and interval files. A STAR-2.7.3a [74] index was generated using the --sjdbOverhang 99 --genomeSAindexNbases 13 --genomeChrBinNbits 14 settings for both genomes concatenated together (to allow measurement of collisions, see below).

Each of the three sub-libraries sequenced in the present manuscript was then processed separately. SPLiTseq toolbox (https://github.com/RebekkaWegmann/splitseq_toolbox), which incorporates many of the components of Drop-seq_tools-2.3.0 (https://github.com/broadinstitute/Drop-seq) was used to extract, check, and correct barcodes (corrections with hamming distance ≤1). Mapping was performed using STAR-2.7.3a [74], with --quantMode GeneCounts and all other default settings. Picard v2.21.1-SNAPSHOT (Broad Institute, http://broadinstitute.github.io/picard/) SortSam and MergeBamAlignment were used to re-order and merge aligned and tagged reads. Drop-seq_tools-2.3.0 TagReadWithInterval and TagReadWithGeneFunction were then run sequentially to note mapping location, using the custom refFlat and genes.intervals files created above. An additional character (A, T, or C) was then added to the cellular barcode of each sub-library, to allow identical cell barcodes from different sub-libraries to be differentiated. Reads mapping to *D. japonica* and *S. mediterranea* were then separated. These mapping files were then used to create expression matrices using Drop-seq_tools-2.3.0 DigitalExpression for each library individually, with the following settings: READ_MQ=0, EDIT_DISTANCE=1, MIN_NUM_GENES_PER_CELL=100, and LOCUS_FUNCTION_LIST=INTRONIC. These matrices, along with the novel gene models and raw reads, have been uploaded to the NCBI GEO, accession GSE150259. Using the matrices produced from the full data for *S. mediterranea* and *D. japonica*, a 2-species “barnyard” plot was built, showing the UMIs per cell barcode from each of the species. Collisions were defined as cell barcodes sharing over 10% of their UMIs with the minority species in this plot.

#### Seurat feature identification, doublet removal, and clustering

The digital expression matrices for each sub-library were loaded into Seurat v 3.1.0 [67] within R v 3.6.2 and a Seurat object created for each, with min.cells = 1 and min.features = 125 (although note the matrix creation cutoff described above). Cells with a UMI count greater than 5000 were excluded with subset = nCount_RNA < 5000. Data was normalized, and variable features selected (selection.method = “vst”, nfeatures = 10,000) and data scaled. The three libraries were then merged (merge (*x* = L1, *y* = list(L2, L3), add.cell.ids = add.cell.ids, merge.data = FALSE)). Data was again normalized (normalization.method = “LogNormalize”, scale.factor = 10,000) and variable features selected (split.by = “library”, nfeatures = 10,000, verbose = TRUE, fvf.nfeatures = 10,000, selection.method = “vst”). Principal component analysis (PCA) was run for 50 principal components. JackStrawPlots and ElbowPlots were generated to understand the dimensionality of the data (plots not shown). Clustering was performed with resolution = 1 (*D. japonica*) or 1.4 (*S. mediterranea*) and the original Louvain algorithm. UMAP projections were displayed both by cluster and by library to assay for batch effects. Further batch effect correction was not necessary. VlnPlot was used to generate violin plots of gene and UMI content in each library, using a log scale. A number of resolutions were trialed and compared manually with marker lists (see “cluster identity assignment” below) to determine the most appropriate cutoff for initial display and further analysis. The resulting objects were then saved in R and used as the basis for doublet identification and removal using DoubletDecon v 1.15 [62]. The Improved Seurat Pre-Process pipeline was run at a range of *ρ*′ values, a min_uniq genes setting of 4 and num_genes = 50. The high-end number of likely within-species doublets was ground-truthed using the number of *S mediterranea*: *D. japonica* collisions seen in our barnyard plot, and the final *ρ*′ value (*D. japonica* 0.3, *S. mediterranea* 0.4) used to identify potential doublets, erring on the side of over-estimation of doublets. Cells identified as potential doublets were removed from our final list of cells carried forward for analysis, along with cells with more than 5000 UMIs. The resulting matrices were then re-analyzed as detailed above. For both species, clustering was performed with resolution = 1.6. UMAP representations were created for both species with the following settings: dims = 1:50, reduction = “pca”, spread = 1, metric = “euclidean”, seed.use = 1, and n.neighbors = 45, min.dist = 0.4*.* Clusters were recolored natively within Seurat as plots were generated. Markers were extracted from Seurat using the FindAllMarkers function. Cell numbers per cluster were extracted using the Idents function. FeaturePlot was used to highlight the expression of individual markers in cells in our dataset, to aid with cell cluster identification.

#### Cluster identity assignment

To establish the identity of *S. mediterranea* cell clusters we cross-referenced the markers found in them with the markers from our previous publication [13]. Representative examples are shown in Additional file 1: Figure S2. Novel cell clusters eye-53 neurons, serotonin neurons, protonephridia tubule cells, and protonephridia flame cells were named respectively by the expression of markers *eye-53-1* (SmMSTRG.4014, dd_Smed_v6_889_0_1) [75], *sert* (SmMSTRG.6717, dd_Smed_v6_12700_0_1) [76, 77], *CAVII-like* (SmMSTRG.5392, dd_Smed_v6_4841_0_1) [78], and *egfr-5* (SmMSTRG.13890, dd_Smed_v6_11310_0_1) [79]. To establish the identity of *D. japonica* cell clusters, we found known *S. mediterranea* homologs of the top *D. japonica* cluster markers as noted below and examined the expression of these in our *S. mediterranea* and *D. japonica* feature maps.

To establish marker homology between our novel gene models and known gene sequences from previous publications, we used a different approach for each species. For *S. mediterranea* comparisons to previously cataloged genes*:* blastn megablast [80] of known nucleotide sequences to our gene models (*E* value cutoff = *E* < 10^−99^, although best hit normally = 0) and Standalone BLAT v. 36 × 5 (-out= blast8) [81] were used to find clearly homologous sequences*.* For *D. japonica*, the same approach was used to find homologs between our novel gene models and known *D. japonica* genes when appropriate. However, to annotate based on known *S. mediterranea* sequences, the protein sequences of previously identified *S. mediterranea* genes were used to search our novel *D. japonica* gene set using tblastn (*E* value cutoff = *E* < 10^−99^), alongside nucleotide vs nucleotide blat searches as described previously. Secretory clusters were arbitrarily named 1–7 and a–h according to their abundance, as the exact correspondence of these with previously published experiments and the homology between planarian species requires further research.

#### Re-mapping of Plass et al. datasets

We re-analyzed the datasets of Plass et al. [13] using the improved gene annotation put forward in this manuscript. These were downloaded from the NCBI GEO at accession GSE103633 and processed using Drop-seq tools 2.3.0 (https://github.com/broadinstitute/Drop-seq) using the Drop­seq Core Computational Protocol v2.0.0. For final matrix generation, the cell barcode list used for the original study was downloaded from https://shiny.mdc-berlin.de/psca/ and used to construct matrices and cells with more than 2500 genes mapped to them, as in that resource, are excluded from our analysis. Note that due to improvements in the Drop-seq DetectBeadSubstitutionErrors and DetectBeadSynthesisErrors pipelines, we recover 21,610 cells, rather than the 21,612 originally used, as the pipeline was able to recognize and correct errors in the last position of the UMI for several cell barcodes, merging several cells. Seurat v 3.1.0 [67] within R v 3.6.2 was then run using the settings described above, with resolution = 1.6, n.neighbors = 35, min.dist = 0.5.

#### Label transfer and integration

Label transfer and integration analyses were performed within R v 3.6.2 and with Seurat v 3.1.0 [57]. Objects were pre-processed (data normalized, variable features selected, data scaled, and PCA run) as described above. For label transfer, FindTransferAnchors was used to identify anchors within the reference set for each comparison, using cca reduction. TransferData was then used to find predicted labels, with pca used for weight reduction, dims = 2:30, and all other default settings.

For integration, FindIntegrationAnchors was run using dims = 1:20 and all other default settings, before IntegrateData was run using the same settings on the output. The resulting integrated object was then processed as described for individual libraries above (using ScaleData, RunPCA, FindNeighbors, FindClusters, and RunUMAP) for display (n.neighbors = 45, min.dist = 0.4, spread = 1, metric = “euclidean”, seed.use = 1). Markers and cell numbers were extracted using FindAllMarkers and from Idents within Seurat.

#### Other analyses performed for single-cell RNA-seq data

To assay whether our library sequencing was saturated at the given read depth, reads were downsampled randomly using seqtk (10%, 25%, 50%, and 75% of total read depth). These read results were extracted from our final bam files and used to generate UMI and gene numbers per cell for the cell barcode set identified from our full results (NB: as bam files were ordered in the process of data generation, sampling from the bam file directly would likely have returned non-random results). seqtk downsampling was also used to generate subsamples seen in Additional file 1: Figure S8. UMI and gene numbers per cluster were displayed using VlnPlot in Seurat v 3.1.0 [57], and the FeaturePlot function was used to color cells within our plots expressing particular gene markers.

## Supplementary Information

**Additional file 1: Supplementary Figures S1-11**.

**Additional file 2: Table S1.** List of barcodes.

**Additional file 3: Table S2.** Cell numbers by cluster and cell type groups.

**Additional file 4: Table S3.** Top markers by cluster.

**Additional file 5: Table S4.** Cluster differential gene expression analysis.

**Additional file 6:.** Review history.

## Data Availability

The datasets supporting the conclusions of this article are available in the GEO Repository, Accession GSE166977 [82] and GSE150259 [83]. This includes all sequencing reads (SRA Accession SRP261093), reannotated gene models for both species analyzed, and the final gene expression matrices used for analysis. These data, as well as further resources, are also available from https://jakke-neiro.github.io/Oxplatys/.
