# Supplementary material for: ACME dissociation: a versatile cell fixation-dissociation method for single-cell transcriptomics
Source: Genome Biol. 2021 Apr 8;22:89. doi: 10.1186/s13059-021-02302-5 (PMC8028764; doi:10.1186/s13059-021-02302-5)
Supplement: Supplementary file 1 — Additional file 1: Supplementary Figures S1-11. [file 13059_2021_2302_MOESM1_ESM.pdf]

Supplementary Figure Legends:

Figure S1: ACME-dissociated cell cytometry profiles in irradiated animals and comparison to trypsin.

A: Flow cytometry ungated and gated profiles of ACME-dissociated cells from non irradiated (left panels) and irradiated (right panels) *S. mediterranea* planarians, stained with DRAQ5 (nucleus) and Concanavalin-A (cytoplasm). The experiment was performed in 3 replicates. Axes are shown in subset logarithmic scale. **B:** Quantification of %G2 cells compared to all gated singlets. The values in the plot correspond to the average of the 3 replicates, and the error bars correspond to the standard deviation. **C:** Flow cytometry ungated and gated profiles of ACME-dissociated cells (top panels) and trypsin-dissociated cells (bottom panels) from *S.mediterranea* planarians stained with DRAQ5 (nucleus). The cytoplasm of ACME-dissociated and trypsin-dissociated cells was stained with Concanavalin-A and Calcein, respectively.

Figure S2: Inference of RIN values.

A: Correlative analysis of RIN values and the % area of the two ribosomal bands compared to the total. The linear correlation is indicated in the chart. **B:** Tables containing the individual values extracted from the RNA samples displayed in Figures 3-4. The individual values not calculated by the RIN algorithm are show in blue as inferred from the linear correlation.

Figure S3: *Nematostella vectensis* metacell analysis.

A: Flow cytometry profiles of ACME-dissociated cells from *Nematostella vectensis,* showing all events (left) and G1/G0 singlets (right). **B:** Expression of 514 variable genes (rows) across 3,899 juvenile *N. vectensis* single cells sorted by metacell and cell type association. Top 30 genes (FC>2) per metacell are included, showing gene functional annotation when available. Transcription factors are highlighted in red. **C:** Total number of RNA molecules (left) and cells (right) per metacell.

Figure S4: SPLiT-seq workflow and sub-library description

A: SPLiT-seq workflow: cells are randomly split in 96-well plates containing unique barcodes. The first RT round captures mRNAs using an anchored-poly dT barcode (BC 1). The second (BC 2) and third barcodes (BC 3, which includes the UMI) are ligated to the cDNA in two subsequent reactions. The last barcode (BC 4) is added in a final PCR reaction after tagmentation. **B:** Structure of the SPLiT-seq library. Paired-end reads (Read 1 and Read 2) and index were sequenced as indicated. **C:** Bioanalyzer profile of the 3 individual sub-libraries described here. **D:** Violin plots showing the distribution of UMI counts per cell in each sub-library for *S. mediterranea* (left) and *D. japonica* (right). **E:** UMAP visualization of *S. mediterranea* cells (top) and *D. japonica* cells (bottom), coloured by sub-library.

Figure S5: Expression of previously published cell type markers in our dataset.

Feature plots of cell type markers from Plass *et al.* in our UMAP visualization of *S. mediterranea* cells.

Figure S6: Expression of germ line progenitor markers.

A: Feature plots of germ line progenitor markers *Smed-eIF3c* and *Smed-nanos* in our *S. mediterranea* dataset and previously published studies: Plass *et al*. (downloaded from https://shiny.mdc-berlin.de/psca/), Fincher *et al*. (downloaded from https://digiworm.wi.mit.edu/) and Zeng *et al*. (downloaded from https://planosphere.stowers.org). **B:** Feature plots of germ line progenitor markers in the *D. japonica* dataset.

Figure S7: Analysis of high UMI-containing cell clusters

A) UMAP visualization of 19,975 ACME-dissociated *S. mediterranea* cells (before >5000 UMI cut-off), and 14,263 ACME-dissociated *D. japonica* cells coloured by UMI content. B) Feature plots of germ line precursor progenitor marker *nanos* in the UMAP visualization of 19,975 *S. mediterranea* cells and 14,263 ACME-dissociated *D. japonica* cells. C) UMAP visualization of the 19,025 ACME-dissociated *S. mediterranea* cells (after >5000 UMI cut-off), and 13,406 ACME-dissociated *D. japonica* cells shown in Fig 7, coloured by UMI content.

Figure S8: Results of downsampling experiments, showing UMI, gene and cluster recovery at a variety of equivalent cutoff points.

Downsampling statistics shown are: Statistics after downsampling García-Castro raw reads to 1,200,007,715 (Plass *et al*. total). Statistics after downsampling all Plass *et al*. reads to 560,518,316 (García-Castro, usable total). Statistics after downsampling both Plass *et al.* and García-Castro results to 10k mapped reads per cell. Also shown are the resulting UMAP representations from these downsampled sets, in the same order as the raw statistics. Note that we observe 40 rather than 41 clusters in our sample when downsampled to approx 88% of original raw read count to match the raw Plass *et al.* number. We see 24 rather than 38 clusters in the Plass *et al*. sample when we downsample these reads to match the number of reads from García-Castro used in analysis (46.7% of the original Plass count). When 10k mapped reads are used per cell, there is no loss of cluster representation in the García-Castro dataset compared to the overall analysis, but 33 (c.f. 38) clusters are seen in the Plass *et al*. data.

Figure S9: **Comparison of results of differential expression analysis, Plass *et al.* vs García-Castro *et al*.**

Comparisons made between neoblast clusters and mature cell types for each dataset as noted. Excellent overlap was seen in recovered genes (74.3 - 84.4% of the DE genes found in ACME-dissociated García-Castro DE analysis were also found in the Trypsin-dissociated Plass *et al.* comparisons). These also show a clear correlation in log fold change values when García-Castro and Plass *et al*. results are compared, in all cell types examined.

Figure S10: Comparison of distribution of UMI counts and genes detected per cluster in this study and Plass *et al*.

A: Violin plots showing the distribution of UMI counts per cluster in ACME-dissociated (left) and trypsin-dissociated (right) *S. mediterranea* cells reanalysed from Plass *et al*. **B:** Violin plots showing the distribution of genes detected per cluster in ACME-dissociated (left) and trypsin-dissociated (right) *S. mediterranea* cells reanalysed from Plass *et al*. Colours of plots match those of the clusters shown in Figs 7A and 8B (ACME and Plass cells respectively) and are ordered according to cluster identity.

Figure S11: Integrative analysis of trypsin- and ACME-dissociated datasets

A: Analysis workflow. **B:** UMAP visualization of 21,610 trypsin-dissociated *S. mediterranea* cells reanalysed from Plass *et al*., coloured by cluster identity and annotated on the basis of marker genes. **C:** UMAP visualisation of 21,610 trypsin-dissociated *S. mediterranea* cells. Cells annotated as germ cells are coloured by transfer labels analysis from ACME-dissociated cells. **D:** Feature plots of germ line progenitor markers *Smed-eIF3c* and *Smed-nanos* in the UMAP visualization of 21,610 trypsin-dissociated *S. mediterranea* cells reanalysed from Plass *et al*. **E:** UMAP visualization of 40,635 cells integrating trypsin-dissociated and ACME-dissociated clusters coloured by transfer labels analysis from trypsin-dissociated cells.

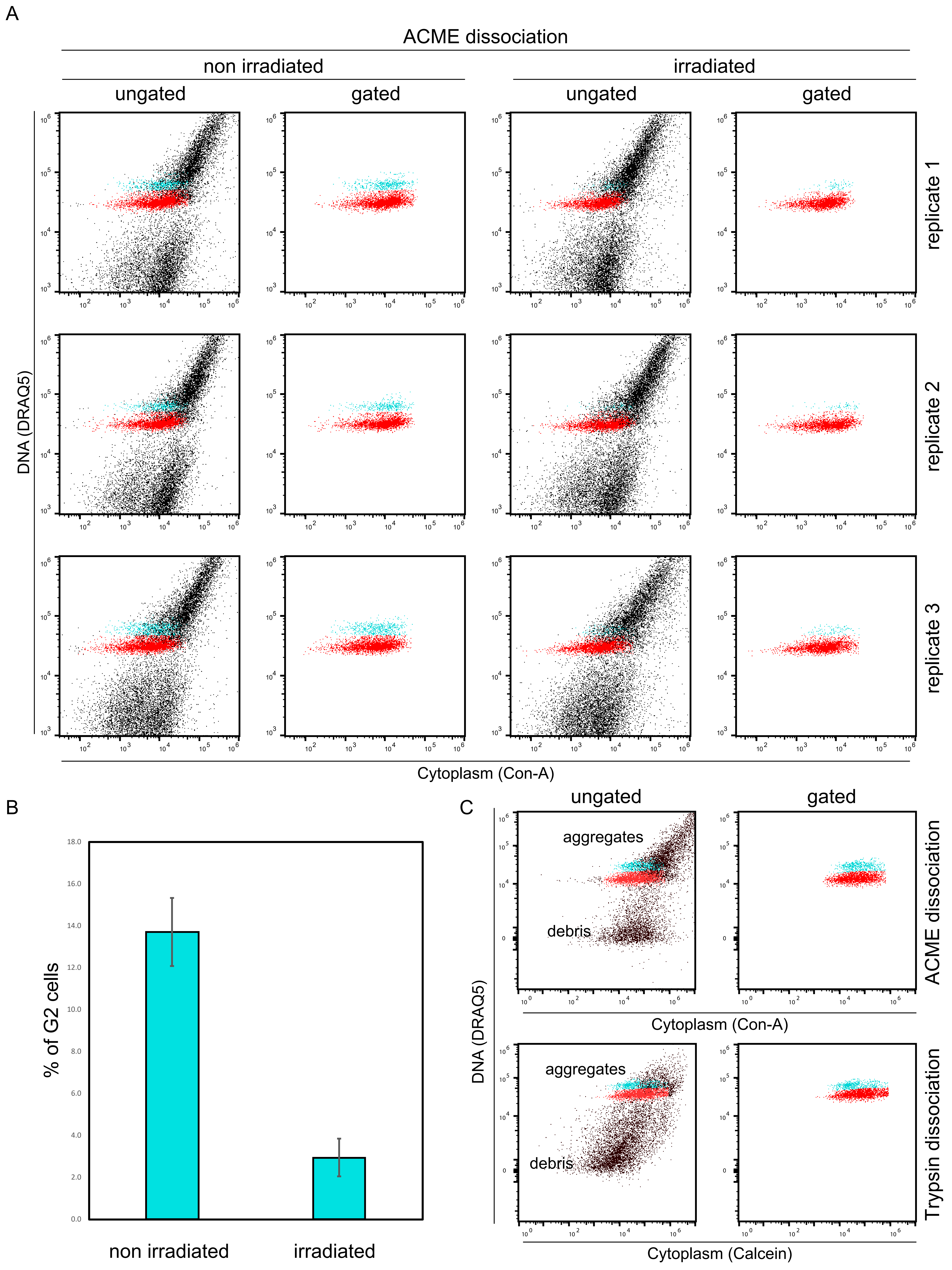

A
ACME dissociation
non irradiated
irradiated
ungated
gated
ungated
gated
replicate 1
replicate 2
replicate 3
DNA (DRAQ5)
Cytoplasm (Con-A)
B
% of G2 cells
non irradiated
irradiated
C
ungated
gated
aggregates
debris
ACME dissociation
Trypsin dissociation
DNA (DRAQ5)
Cytoplasm (Con-A)
Cytoplasm (Calcein)

A

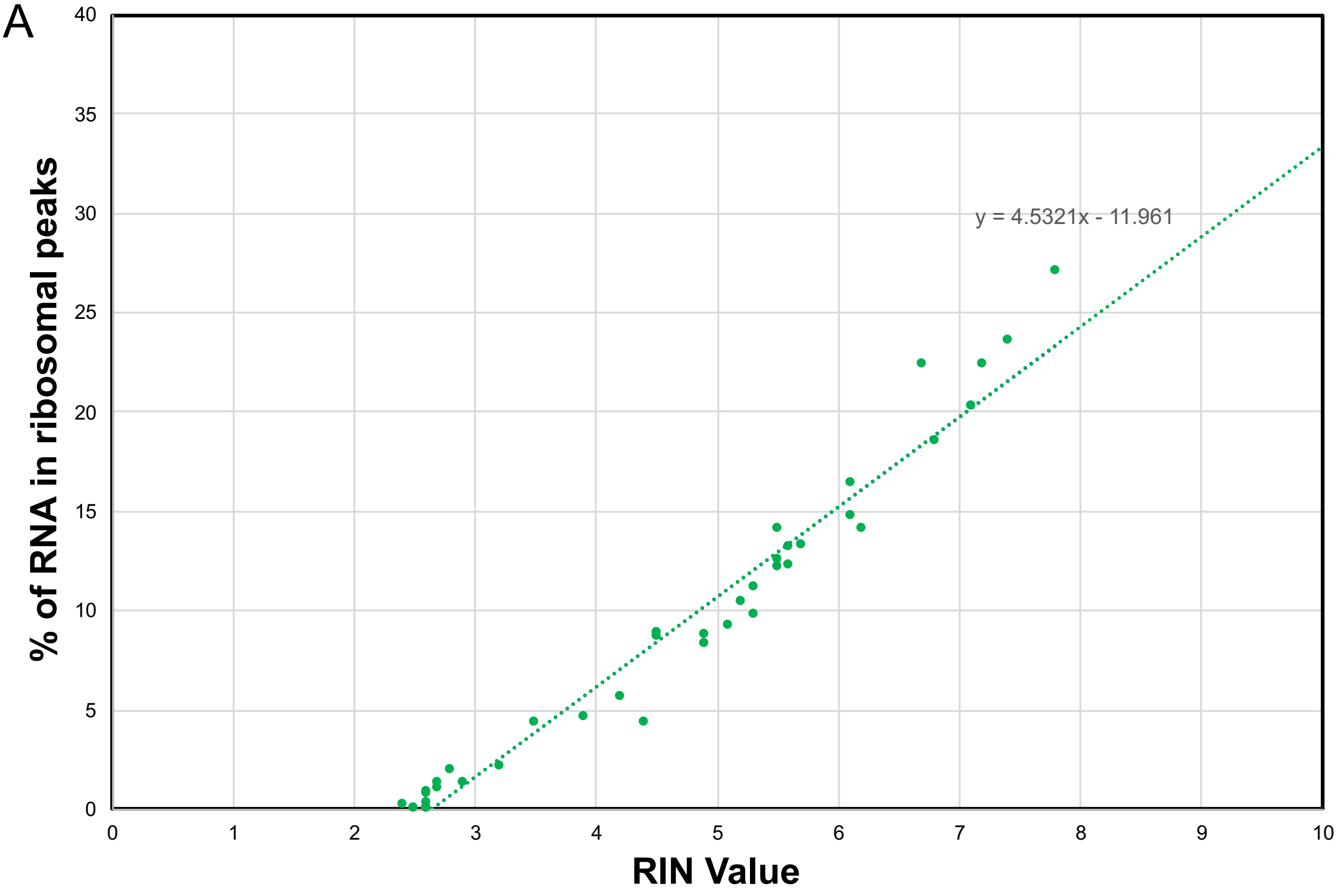

B

Figure 3H	RIN	RNA conc (ng/uL)	% in peaks
t= 0	7.8	143	27
1 hour	3.5	148	4.3
2 hours	3.2	114	2.1
3 hours	2.8	170	1.9
4 hours	2.9	187	1.3
5 hours	2.7	222	1.3
6 hours	2.6	218	0.8

Figure 3I	RIN	RNA conc (ng/uL)	% in peaks
t= 0	7.2	147	22.3
1 hour	6.1	192	14.7
2 hours	4.5	192	8.8
3 hours	4.9	141	8.7
4 hours	5.1	206	9.2
5 hours	4.9	233	8.3
6 hours	4.5	275	8.6

Figure 3K	RIN	RNA conc (ng/uL)	% in peaks
1 cycle	6.7	151	22.3
2 cycles	6.1	205	16.4
3 cycles	5.7	192	13.2
4 cycles	5.5	150	12.5
5 cycles	5.3	162	9.7

Figure 3G	RIN	RNA conc (ng/uL)	% in peaks	Inferred RIN
Control	N/C	105	46.4	10

Figure 3J	RIN	RNA conc (ng/uL)	% in peaks	Inferred RIN
B	N/C	161	28.7	9.0
C	7.4	243	23.5	-
D	4.2	211	5.6	-
E	5.5	292	14.1	-

Figure 4C	RIN	RNA conc (ng/uL)	% in peaks	Inferred RIN
Trypsin	6.2	216	14.1	-
Try + Mace	3.9	272	4.6	-
Try + 0.5 FA	N/C	192	5.5	3.9
Try + 1 FA	2.5	145	0	-
Try + 2 FA	2.5	212	0	-
Try + 3 FA	2.6	210	0	-
Try + 4 FA	2.6	143	0	-

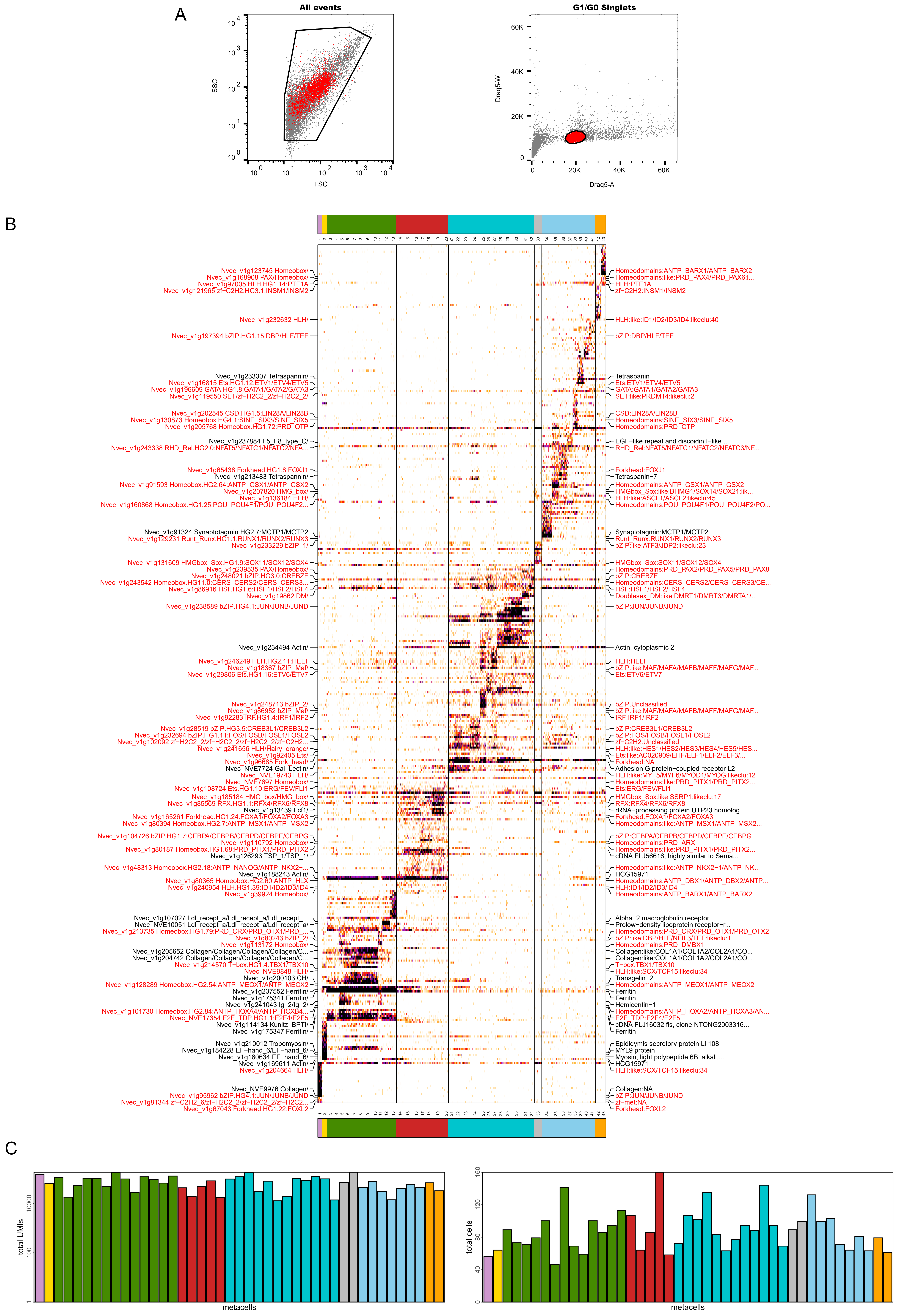

A
All events
SSC
FSC
G1/G0 Singlets
Draq5-W
Draq5-A
60K
40K
20K
0
B
Nvec_v1g123745 Homeobox/
Nvec_v1g168908 PAX/Homeobox/
Nvec_v1g97005 HLH.HG1.14:PTF1A
Nvec_v1g121965 zf-C2H2.HG3.1:INSM1/INSM2
Nvec_v1g232632 HLH/
Nvec_v1g197394 bZIP.HG1.15:DBP/HLF/TEF
Nvec_v1g233307 Tetraspannin/
Nvec_v1g16815 Ets.HG1.12:ETV1/ETV4/ETV5
Nvec_v1g196609 GATA.HG1.8:GATA1/GATA2/GATA3
Nvec_v1g119550 SET/zf-H2C2_2/zf-H2C2_2/
Nvec_v1g202545 CSD.HG1.5:LIN28A/LIN28B
Nvec_v1g130873 Homeobox.HG4.1:SINE_SIX3/SINE_SIX5
Nvec_v1g205768 Homeobox.HG1.72:PRD_OTP
Nvec_v1g237884 F5_F8_type_C/
Nvec_v1g243338 RHD_Rel.HG2.0:NFAT5/NFATC1/NFATC2/NFA...
Nvec_v1g65438 Forkhead.HG1.8:FOXJ1
Nvec_v1g213483 Tetraspannin/
Nvec_v1g91593 Homeobox.HG2.64:ANTP_GSX1/ANTP_GSX2
Nvec_v1g207820 HMG_box/
Nvec_v1g136184 HLH/
Nvec_v1g160868 Homeobox.HG1.25:POU_POU4F1/POU_POU4F2...
Nvec_v1g91324 Synaptotagmin.HG2.7:MCTP1/MCTP2
Nvec_v1g129231 Runt_Runx.HG1.1:RUNX1/RUNX2/RUNX3
Nvec_v1g233229 bZIP_1/
Nvec_v1g131609 HMGbox_Sox.HG1.9:SOX11/SOX12/SOX4
Nvec_v1g239535 PAX/Homeobox/
Nvec_v1g248021 bZIP.HG3.0:CREBZF
Nvec_v1g243542 Homeobox.HG11.0:CERS_CERS2/CERS_CERS3...
Nvec_v1g86916 HSF.HG1.6:HSF1/HSF2/HSF4
Nvec_v1g19862 DM/
Nvec_v1g238589 bZIP.HG4.1:JUN/JUNB/JUND
Nvec_v1g234494 Actin/
Nvec_v1g246249 HLH.HG2.11:HELT
Nvec_v1g18367 bZIP_Maf/
Nvec_v1g29806 Ets.HG1.16:ETV6/ETV7
Nvec_v1g248713 bZIP_2/
Nvec_v1g86952 bZIP_Maf/
Nvec_v1g92283 IRF.HG1.4:IRF1/IRF2
Nvec_v1g28519 bZIP.HG3.5:CREB3L1/CREB3L2
Nvec_v1g232694 bZIP.HG1.11:FOS/FOSB/FOSL1/FOSL2
Nvec_v1g102092 zf-H2C2_2/zf-H2C2_2/zf-C2H2...
Nvec_v1g241656 HLH/Hairy_orange/
Nvec_v1g92405 Ets/
Nvec_v1g96685 Fork_head/
Nvec_NVE7724 Gal_Lectin/
Nvec_NVE19743 HLH/
Nvec_NVE7697 Homeobox/
Nvec_v1g108724 Ets.HG1.10:ERG/FEV/FLI1
Nvec_v1g185184 HMG_box/HMG_box/
Nvec_v1g85569 RFX.HG1.1:RFX4/RFX6/RFX8
Nvec_v1g13439 Fcf1/
Nvec_v1g165261 Forkhead.HG1.24:FOXA1/FOXA2/FOXA3
Nvec_v1g80394 Homeobox.HG2.7:ANTP_MSX1/ANTP_MSX2
Nvec_v1g104726 bZIP.HG1.7:CEBPA/CEBPB/CEBPD/CEBPE/CEBPG
Nvec_v1g110792 Homeobox/
Nvec_v1g80187 Homeobox.HG1.68:PRD_PITX1/PRD_PITX2
Nvec_v1g126293 TSP_1/TSP_1/
Nvec_v1g48313 Homeobox.HG2.18:ANTP_NANOG/ANTP_NKX2-...
Nvec_v1g188243 Actin/
Nvec_v1g80365 Homeobox.HG2.60:ANTP_HLX
Nvec_v1g240954 HLH.HG1.39:ID1/ID2/ID3/ID4
Nvec_v1g39924 Homeobox/
Nvec_v1g107027 Ldl_recept_a/Ldl_recept_a/Ldl_recept_...
Nvec_NVE10051 Ldl_recept_a/Ldl_recept_a/
Nvec_v1g213735 Homeobox.HG1.79:PRD_CRX/PRD_OTX1/PRD_...
Nvec_v1g80243 bZIP_2/
Nvec_v1g113172 Homeobox/
Nvec_v1g205652 Collagen/Collagen/Collagen/Collagen/C...
Nvec_v1g204742 Collagen/Collagen/Collagen/Collagen/C...
Nvec_v1g214570 T-box.HG1.4:TBX1/TBX10
Nvec_NVE9848 HLH/
Nvec_v1g200103 CH/
Nvec_v1g128289 Homeobox.HG2.54:ANTP_MEOX1/ANTP_MEOX2
Nvec_v1g237552 Ferritin/
Nvec_v1g175341 Ferritin/
Nvec_v1g241043 Ig_2/Ig_2/
Nvec_v1g101730 Homeobox.HG2.84:ANTP_HOXA4/ANTP_HOXB4...
Nvec_NVE17354 E2F_TDP.HG1.1:E2F4/E2F5
Nvec_v1g114134 Kunitz_BPTI/
Nvec_v1g175347 Ferritin/
Nvec_v1g210012 Tropomyosin/
Nvec_v1g184228 EF-hand_6/EF-hand_6/
Nvec_v1g160634 EF-hand_6/
Nvec_v1g169611 Actin/
Nvec_v1g204664 HLH/
Nvec_NVE9976 Collagen/
Nvec_v1g95962 bZIP.HG4.1:JUN/JUNB/JUND
Nvec_v1g81344 zf-C2H2_6/zf-H2C2_2/zf-H2C2_2/zf-H2C2...
Nvec_v1g67043 Forkhead.HG1.22:FOXL2
Homeodomains:ANTP_BARX1/ANTP_BARX2
Homeodomains:like:PRD_PAX4/PRD_PAX6:l...
HLH:PTF1A
zf-C2H2:INSM1/INSM2
HLH:like:ID1/ID2/ID3/ID4:likeclu:40
bZIP:DBP/HLF/TEF
Tetraspanin
Ets:ETV1/ETV4/ETV5
GATA:GATA1/GATA2/GATA3
SET:like:PRDM14:likeclu:2
CSD:LIN28A/LIN28B
Homeodomains:SINE_SIX3/SINE_SIX5
Homeodomains:PRD_OTP
EGF-like repeat and discoidin I-like ...
RHD_Rel:NFAT5/NFATC1/NFATC2/NFATC3/NF...
Forkhead:FOXJ1
Tetraspanin-7
Homeodomains:ANTP_GSX1/ANTP_GSX2
HMGbox_Sox:like:BHMG1/SOX14/SOX21:lik...
HLH:like:ASCL1/ASCL2:likeclu:45
Homeodomains:POU_POU4F1/POU_POU4F2/PO...
Synaptotagmin:MCTP1/MCTP2
Runt_Runx:RUNX1/RUNX2/RUNX3
bZIP:like:ATF3/JDP2:likeclu:23
HMGbox_Sox:SOX11/SOX12/SOX4
Homeodomains:PRD_PAX2/PRD_PAX5/PRD_PAX8
bZIP:CREBZF
Homeodomains:CERS_CERS2/CERS_CERS3/CE...
HSF:HSF1/HSF2/HSF4
Doublesex_DM:like:DMRT1/DMRT3/DMRTA1/...
bZIP:JUN/JUNB/JUND
Actin, cytoplasmic 2
HLH:HELT
bZIP:like:MAF/MAFA/MAFB/MAFF/MAFG/MAF...
Ets:ETV6/ETV7
bZIP.Unclassified
bZIP:like:MAF/MAFA/MAFB/MAFF/MAFG/MAF...
IRF:IRF1/IRF2
bZIP:CREB3L1/CREB3L2
bZIP:FOS/FOSB/FOSL1/FOSL2
zf-C2H2.Unclassified
HLH:like:HES1/HES2/HES3/HES4/HES5/HES...
Ets:like:AC020909/EHF/ELF1/ELF2/ELF3/...
Forkhead:NA
Adhesion G protein-coupled receptor L2
HLH:like:MYF5/MYF6/MYOD1/MYOG:likeclu:12
Homeodomains:like:PRD_PITX1/PRD_PITX2...
Ets:ERG/FEV/FLI1
HMGbox_Sox:like:SSRP1:likeclu:17
RFX:RFX4/RFX6/RFX8
rRNA-processing protein UTP23 homolog
Forkhead:FOXA1/FOXA2/FOXA3
Homeodomains:like:ANTP_MSX1/ANTP_MSX2...
bZIP:CEBPA/CEBPB/CEBPD/CEBPE/CEBPG
Homeodomains:PRD_ARX
Homeodomains:like:PRD_PITX1/PRD_PITX2...
cDNA FLJ56616, highly similar to Sema...
Homeodomains:like:ANTP_NKX2-1/ANTP_NK...
HCG15971
Homeodomains:ANTP_DBX1/ANTP_DBX2/ANTP...
HLH:ID1/ID2/ID3/ID4
Homeodomains:ANTP_BARX1/ANTP_BARX2
Alpha-2 macroglobulin receptor
Prolow-density lipoprotein receptor-r...
Homeodomains:PRD_CRX/PRD_OTX1/PRD_OTX2
bZIP:like:DBP/HLF/NFIL3/TEF:likeclu:1...
Homeodomains:PRD_DMBX1
Collagen:like:COL1A1/COL1A2/COL2A1/CO...
Collagen:like:COL1A1/COL1A2/COL2A1/CO...
T-box:TBX1/TBX10
HLH:like:SCX/TCF15:likeclu:34
Transgelin-2
Homeodomains:ANTP_MEOX1/ANTP_MEOX2
Ferritin
Ferritin
Hemicentin-1
Homeodomains:ANTP_HOXA2/ANTP_HOXA3/AN...
E2F_TDP:E2F4/E2F5
cDNA FLJ16032 fis, clone NTONG2003316...
Ferritin
Epididymis secretory protein Li 108
MYL9 protein
Myosin, light polypeptide 6B, alkali,...
HCG15971
HLH:like:SCX/TCF15:likeclu:34
Collagen:NA
bZIP:JUN/JUNB/JUND
zf-met:NA
Forkhead:FOXL2
C
total UMIs
metacells
total cells
metacells

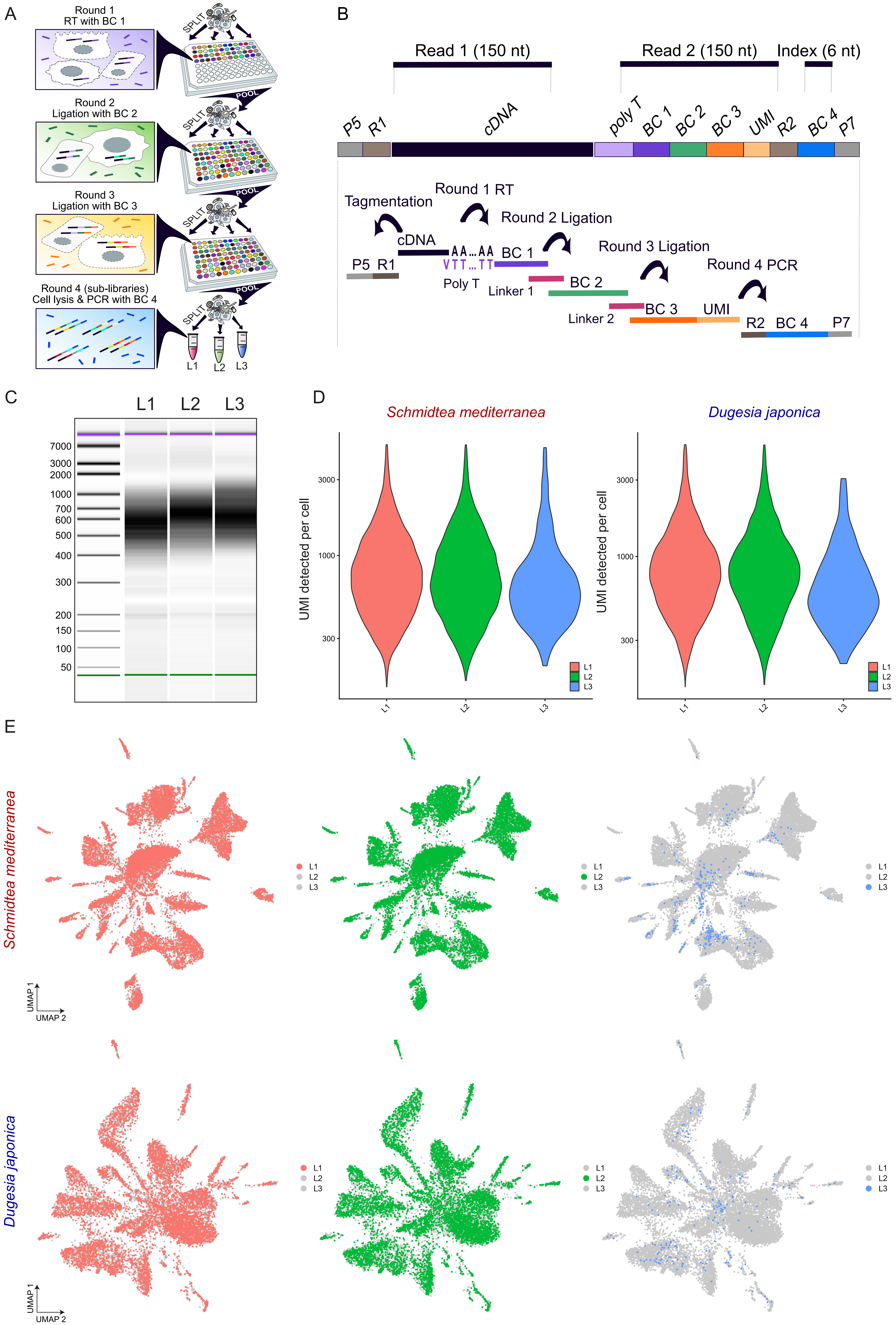
A
Round 1
RT with BC 1
Round 2
Ligation with BC 2
Round 3
Ligation with BC 3
Round 4 (sub-libraries)
Cell lysis & PCR with BC 4
SPLIT
POOL
L1
L2
L3
B
Read 1 (150 nt)
Read 2 (150 nt)
Index (6 nt)
P5
R1
cDNA
poly T
BC 1
BC 2
BC 3
UMI
R2
BC 4
P7
Tagmentation
Round 1 RT
Round 2 Ligation
Round 3 Ligation
Round 4 PCR
AA…AA
VTT…TT
Poly T
Linker 1
Linker 2
C
L1
L2
L3
7000
3000
2000
1000
700
600
500
400
300
200
150
100
50
D
Schmidtea mediterranea
Dugesia japonica
UMI detected per cell
3000
1000
300
E
UMAP 1
UMAP 2

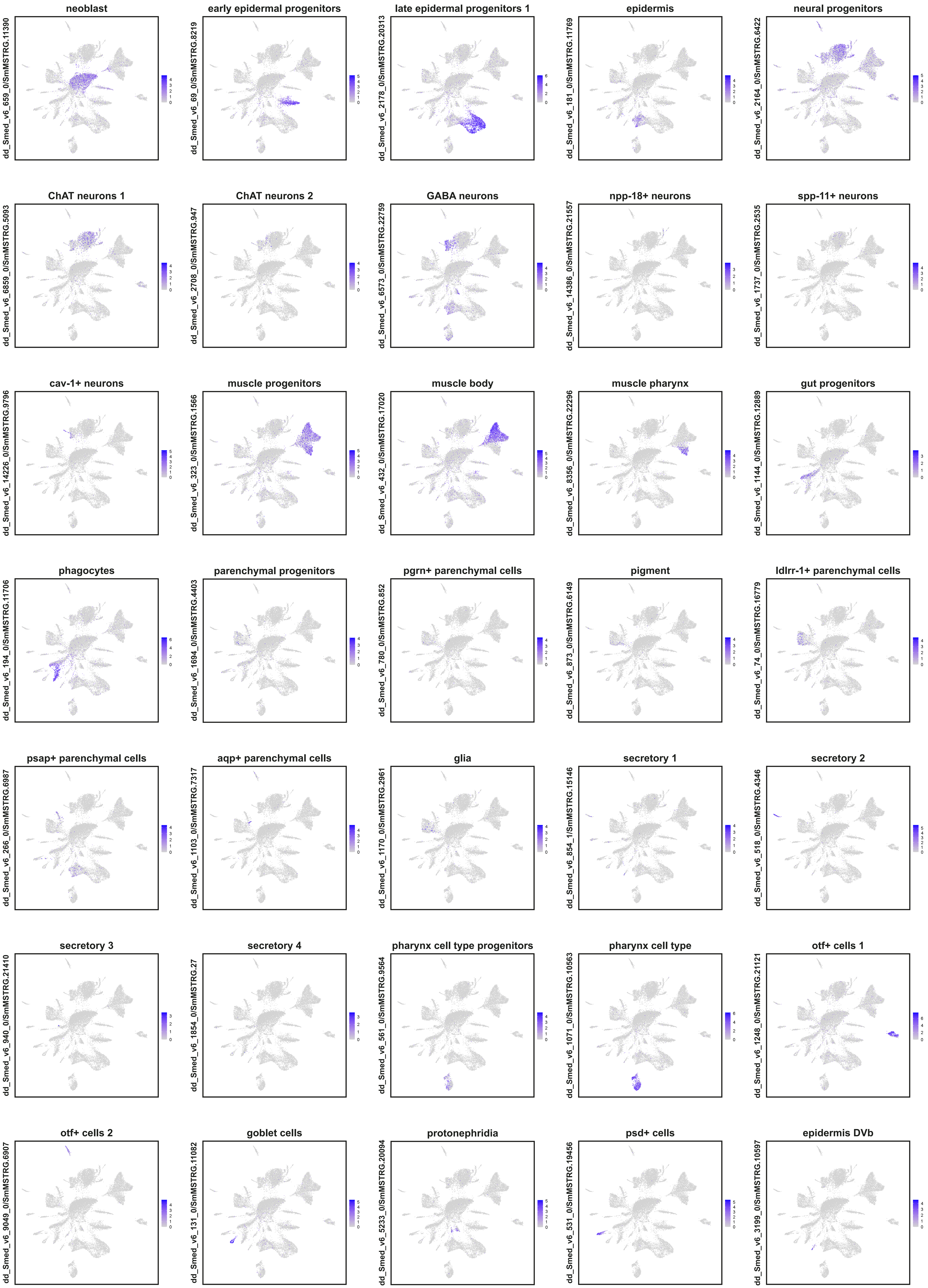

neoblast
dd_Smed_v6_659_0/SmMSTRG.11390
early epidermal progenitors
dd_Smed_v6_69_0/SmMSTRG.8219
late epidermal progenitors 1
dd_Smed_v6_2178_0/SmMSTRG.20313
epidermis
dd_Smed_v6_181_0/SmMSTRG.11769
neural progenitors
dd_Smed_v6_2164_0/SmMSTRG.6422
ChAT neurons 1
dd_Smed_v6_6859_0/SmMSTRG.5093
ChAT neurons 2
dd_Smed_v6_2708_0/SmMSTRG.947
GABA neurons
dd_Smed_v6_6573_0/SmMSTRG.22759
npp-18+ neurons
dd_Smed_v6_14386_0/SmMSTRG.21557
spp-11+ neurons
dd_Smed_v6_1737_0/SmMSTRG.2535
cav-1+ neurons
dd_Smed_v6_14226_0/SmMSTRG.9796
muscle progenitors
dd_Smed_v6_323_0/SmMSTRG.1566
muscle body
dd_Smed_v6_432_0/SmMSTRG.17020
muscle pharynx
dd_Smed_v6_8356_0/SmMSTRG.22296
gut progenitors
dd_Smed_v6_1144_0/SmMSTRG.12889
phagocytes
dd_Smed_v6_194_0/SmMSTRG.11706
parenchymal progenitors
dd_Smed_v6_1694_0/SmMSTRG.4403
pgrn+ parenchymal cells
dd_Smed_v6_780_0/SmMSTRG.852
pigment
dd_Smed_v6_873_0/SmMSTRG.6149
ldlrr-1+ parenchymal cells
dd_Smed_v6_74_0/SmMSTRG.16779
psap+ parenchymal cells
dd_Smed_v6_266_0/SmMSTRG.6987
aqp+ parenchymal cells
dd_Smed_v6_1103_0/SmMSTRG.7317
glia
dd_Smed_v6_1170_0/SmMSTRG.2961
secretory 1
dd_Smed_v6_854_1/SmMSTRG.15146
secretory 2
dd_Smed_v6_518_0/SmMSTRG.4346
secretory 3
dd_Smed_v6_940_0/SmMSTRG.21410
secretory 4
dd_Smed_v6_1854_0/SmMSTRG.27
pharynx cell type progenitors
dd_Smed_v6_561_0/SmMSTRG.9564
pharynx cell type
dd_Smed_v6_1071_0/SmMSTRG.10563
otf+ cells 1
dd_Smed_v6_1248_0/SmMSTRG.21121
otf+ cells 2
dd_Smed_v6_9049_0/SmMSTRG.6907
goblet cells
dd_Smed_v6_131_0/SmMSTRG.11082
protonephridia
dd_Smed_v6_5233_0/SmMSTRG.20094
psd+ cells
dd_Smed_v6_531_0/SmMSTRG.19456
epidermis DVb
dd_Smed_v6_3199_0/SmMSTRG.10597

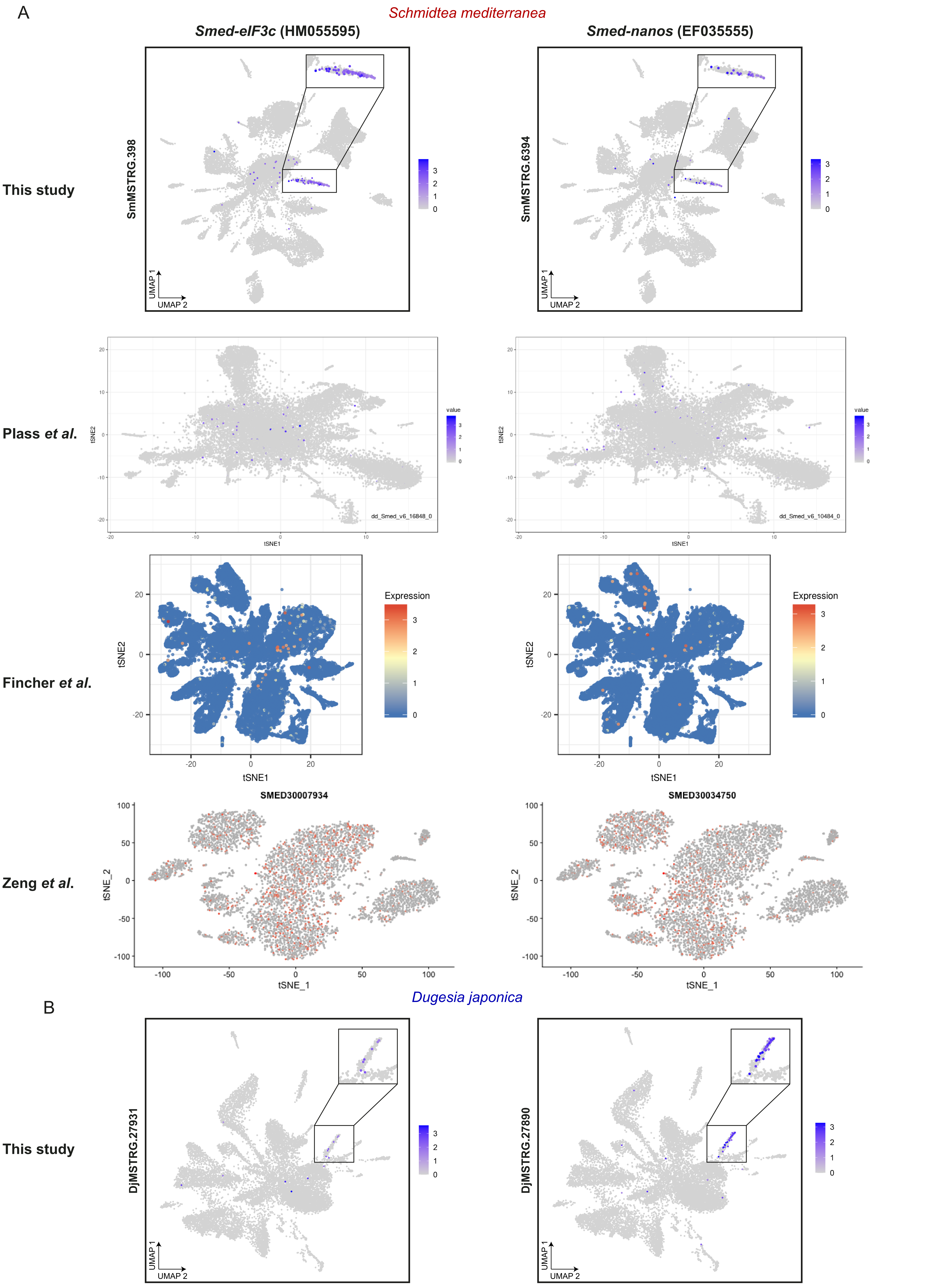
A
Schmidtea mediterranea
Smed-eIF3c (HM055595)
Smed-nanos (EF035555)
This study
SmMSTRG.398
SmMSTRG.6394
UMAP 1
UMAP 2
Plass et al.
value
tSNE2
tSNE1
dd_Smed_v6_16848_0
dd_Smed_v6_10484_0
Fincher et al.
Expression
Zeng et al.
SMED30007934
SMED30034750
tSNE_2
tSNE_1
B
Dugesia japonica
DjMSTRG.27931
DjMSTRG.27890

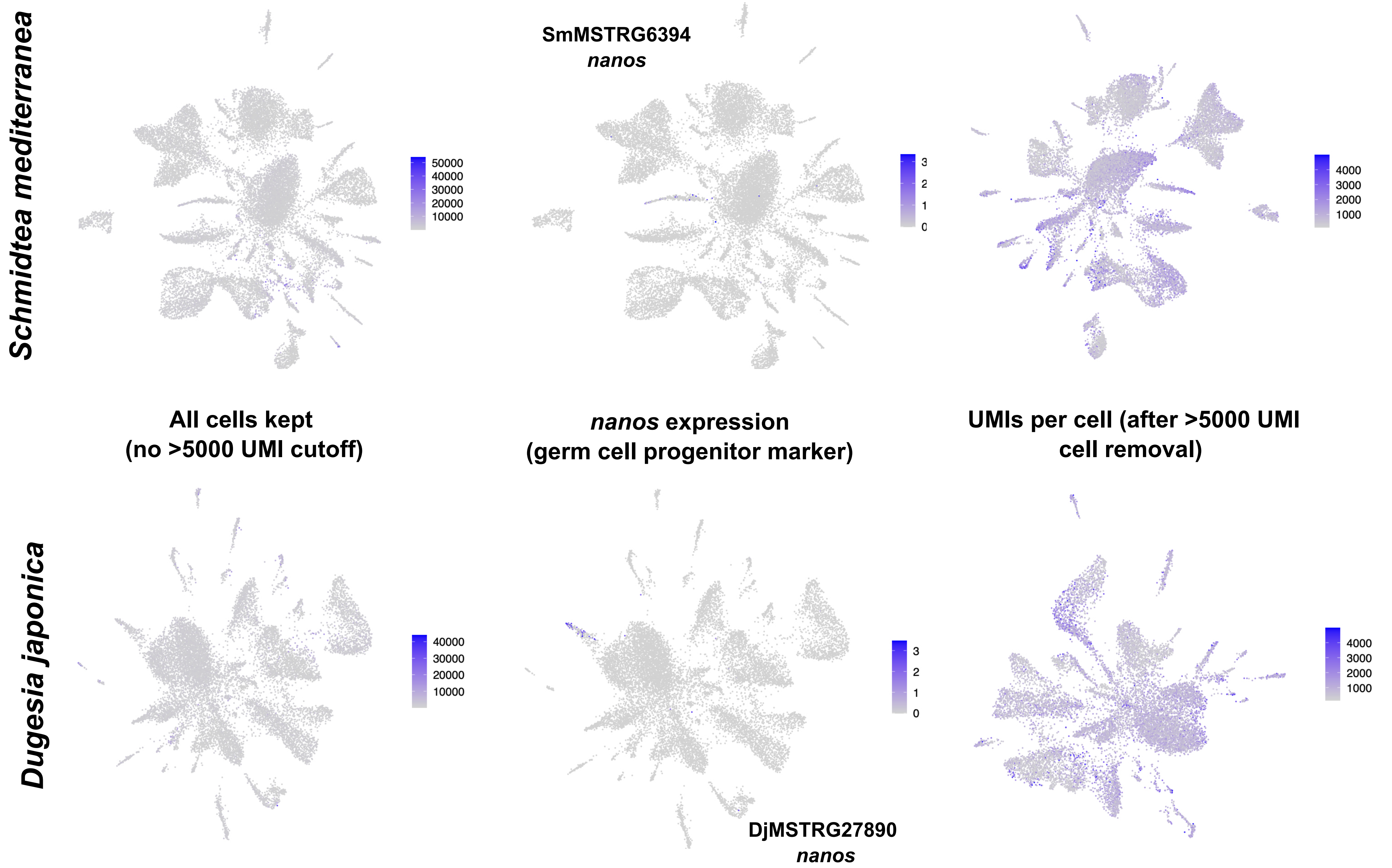

Schmidtea mediterranea
Dugesia japonica
SmMSTRG6394
nanos
DjMSTRG27890
nanos
50000
40000
30000
20000
10000
3
2
1
0
4000
3000
2000
1000
All cells kept
(no >5000 UMI cutoff)
nanos expression
(germ cell progenitor marker)
UMIs per cell (after >5000 UMI
cell removal)

Statistics on UMI and gene count:

	Plass et al (this reanalysis)	García-Castro et al (this dataset)
**All reads**	1,200,007,715	1,362,741,948
**Usable reads**	1,200,007,715	560,518,316
**Mapped reads (to genome)**	399,074,916	470,671,761
**Mapped reads (to genes)**	343,409,649	427,296,897
**Mapped reads (intronic content)**	81,299,126	161,656,944
**Total cells**	21,610	32,431
**Total UMIs**	21,288,321	29,557,182
**Total genes**	12,233,806	6,837,300
Statistics for each sample (nb in García-Castro, 2 species combined)		
**UMIs per cell (mean)**	985.1	911.4
**Genes per cell (mean)**	566.1	210.8
**UMIs per cell (median)**	696	741
**Genes per cell (median)**	452	179
**UMI per million raw reads**	17,740.2	21,689.5
**Genes per million raw reads**	10,194.8	5,017.3
**UMI per million usable reads**	17,740.2	52,731.9
**Genes per million usable reads**	10,194.8	12,198.2
Statistics after downsampling <u>all</u> García-Castro reads to 1,200,007,715 (Plass total)		
**UMIs**	21,288,321	27,939,680
**Genes**	12,233,806	6,581,986
**UMIs per cell (mean)**	939.8	861.5
**Genes per cell (mean)**	541.8	203.0
**UMIs per cell (median)**	696	700
**Genes per cell (median)**	452	172
**UMI per million reads**	17,740.2	23,282.9
**Genes per million reads**	10,194.8	5,485.0
Statistics after downsampling both to 10k <u>mapped</u> reads per cell		
**Mapped Reads**	216,100,000	324,310,000
**UMIs**	17,858,300	28,840,440
**Genes**	10,569,570	6,719,416
**UMIs per cell (mean)**	826.4	889.3
**Genes per cell (mean)**	489.1	207.2
**UMIs per cell (median)**	594	723
**Genes per cell (median)**	394	176
**UMI per million reads**	82,639.1	88,928.6
**Genes per million reads**	48,910.6	20,719.1

García-Castro downsampled to Plass Total

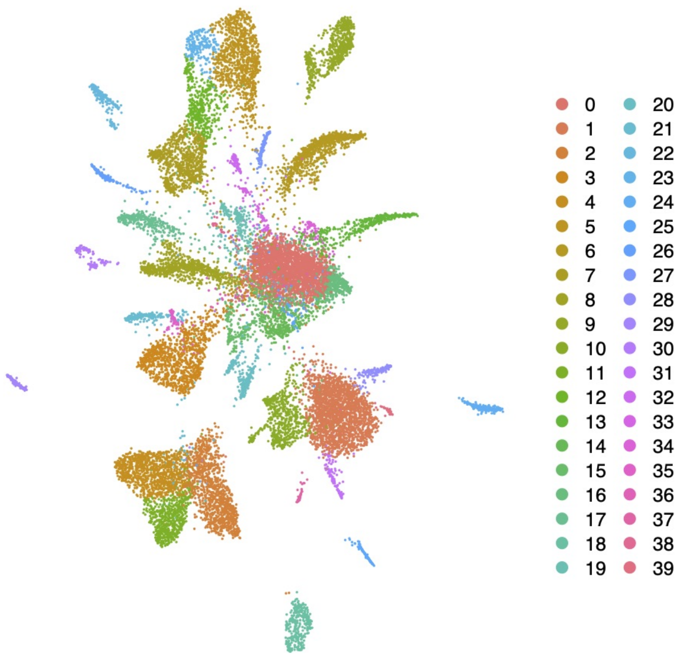

García-Castro Downsampeld to 10k Per Cell

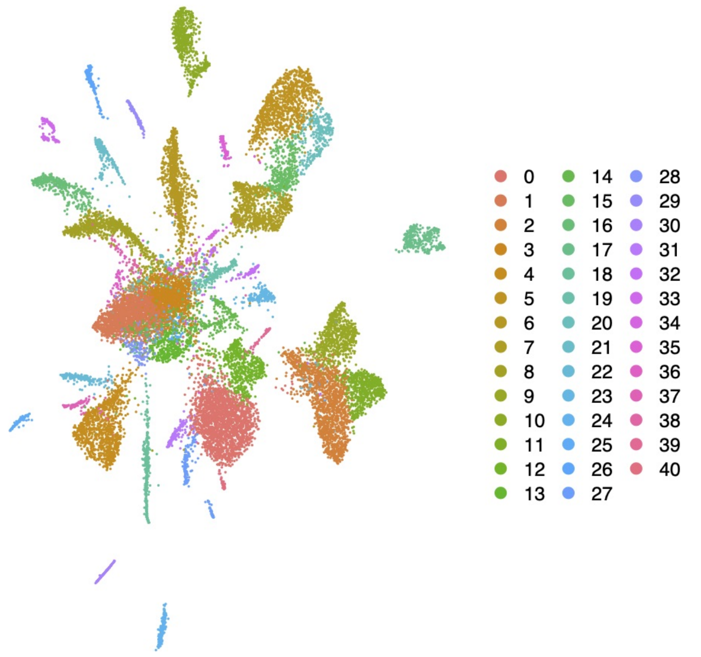

Plass downsampled to 10k Mapped Per Cell

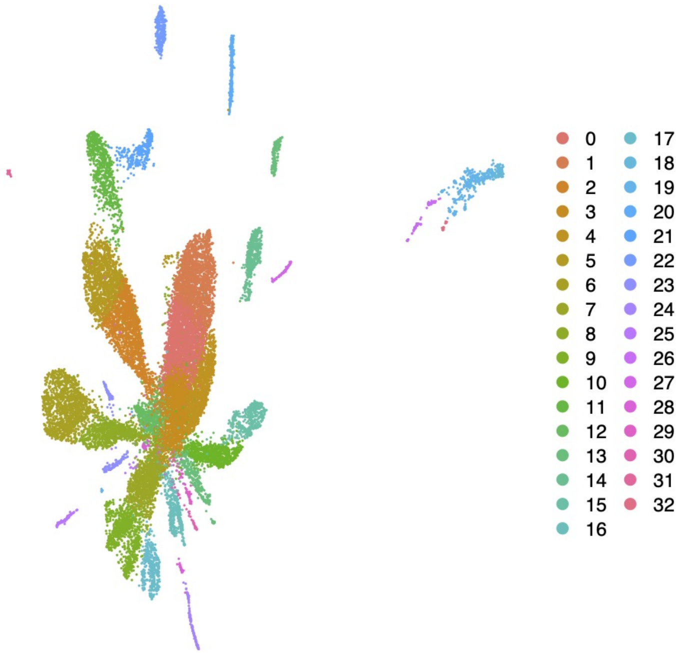

	Number of genes differentially expressed in ACME-dissociated dataset	Number of overlaps with Trypsin dissociated dataset (Plass et al)	Percentage overlap	Neoblast cluster numbers (ACME-dissociated dataset, Garcia-Castro)	Test clusters (ACME-dissociated dataset, Garcia-Castro et al)	Neoblast cluster numbers (Trypsin dissociated dataset, Plass et al)	Test clusters (Trypsin dissociated dataset, Plass et al)
Neoblast vs GABA neuron	333	281	84.4	1, 2, 15, 17, 40	10	0, 1, 4, 6, 12, 17, 25, 33	16
Neoblast vs differentiated Goblet	365	304	83.3	1, 2, 15, 17, 40	32	0, 1, 4, 6, 12, 17, 25, 33	37
Neoblast vs muscle	354	272	76.8	1, 2, 15, 17, 40	3, 6, 11	0, 1, 4, 6, 12, 17, 25, 33	7, 8, 19
Neoblast vs Otf	429	354	82.5	1, 2, 15, 17, 40	16, 21	0, 1, 4, 6, 12, 17, 25, 33	28, 29
Neoblast vs Pharynx	421	313	74.3	1, 2, 15, 17, 40	13, 23	0, 1, 4, 6, 12, 17, 25, 33	26

Differential expression analysis performed in Seurat with FindMarkers, with a Wilcoxon Rank Sum test, logfc.threshold of 0.25, and a min.pct value of 0.1

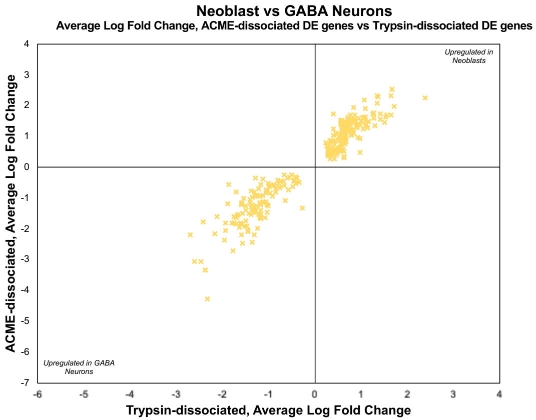

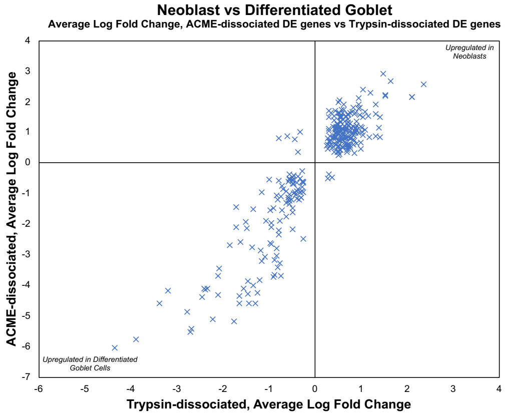

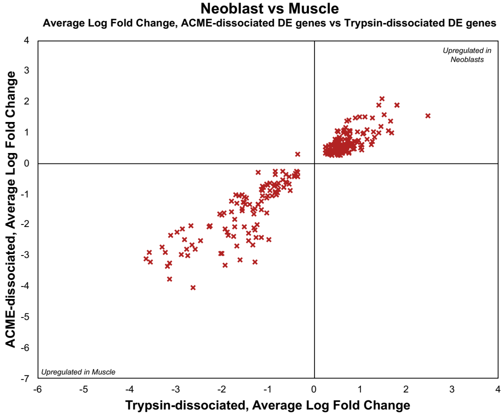

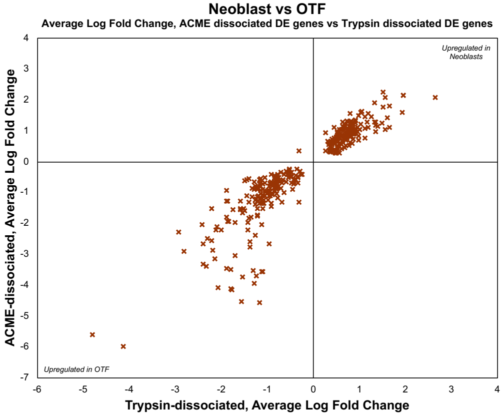

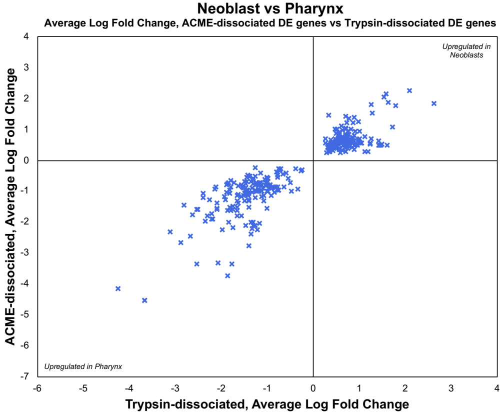

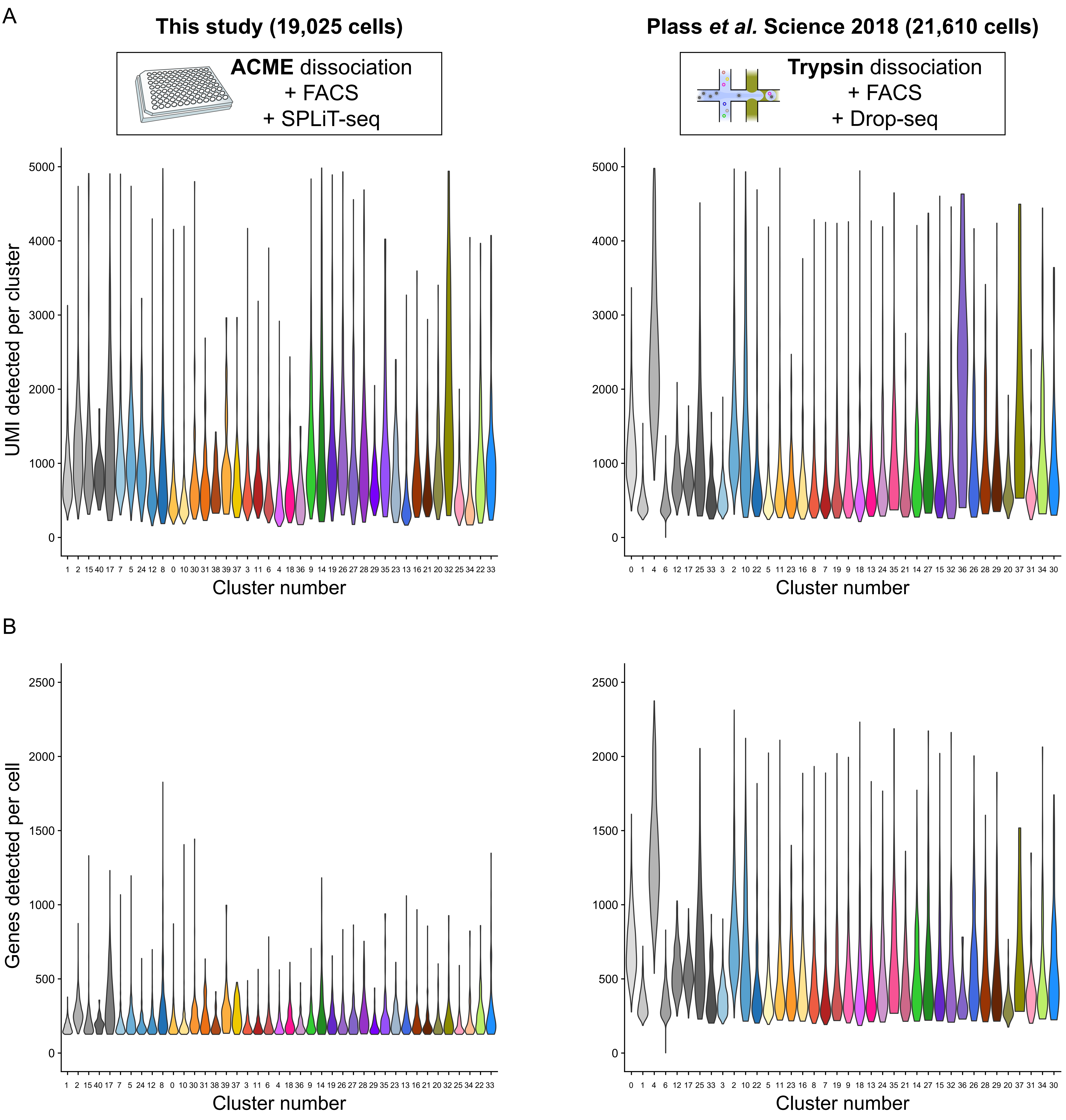
A
This study (19,025 cells)
ACME dissociation
+ FACS
+ SPLiT-seq
Plass et al. Science 2018 (21,610 cells)
Trypsin dissociation
+ FACS
+ Drop-seq
UMI detected per cluster
0
1000
2000
3000
4000
5000
1 2 15 40 17 7 5 24 12 8 0 10 30 31 38 39 37 3 11 6 4 18 36 9 14 19 26 27 28 29 35 23 13 16 21 20 32 25 34 22 33
Cluster number
0 1 4 6 12 17 25 33 3 2 10 22 5 11 23 16 8 7 19 9 18 13 24 35 21 14 27 15 32 36 26 28 29 20 37 31 34 30
Cluster number
B
Genes detected per cell
0
500
1000
1500
2000
2500
1 2 15 40 17 7 5 24 12 8 0 10 30 31 38 39 37 3 11 6 4 18 36 9 14 19 26 27 28 29 35 23 13 16 21 20 32 25 34 22 33
Cluster number
0 1 4 6 12 17 25 33 3 2 10 22 5 11 23 16 8 7 19 9 18 13 24 35 21 14 27 15 32 36 26 28 29 20 37 31 34 30
Cluster number

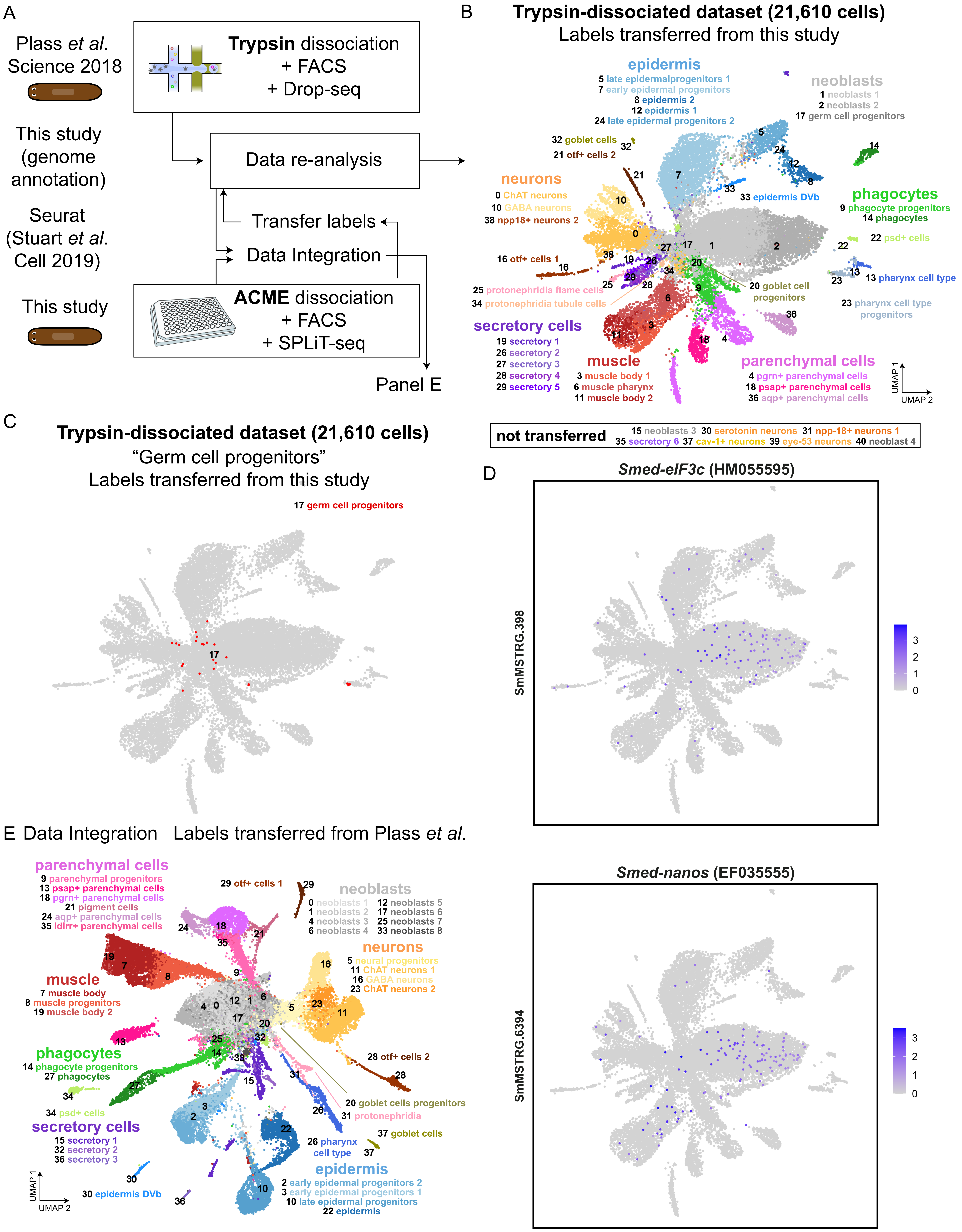
A
Plass et al. Science 2018
This study (genome annotation)
Seurat (Stuart et al. Cell 2019)
This study
Trypsin dissociation + FACS + Drop-seq
Data re-analysis
Transfer labels
Data Integration
ACME dissociation + FACS + SPLiT-seq
Panel E
B
Trypsin-dissociated dataset (21,610 cells)
Labels transferred from this study
epidermis
5 late epidermalprogenitors 1
7 early epidermal progenitors
8 epidermis 2
12 epidermis 1
24 late epidermal progenitors 2
neoblasts
1 neoblasts 1
2 neoblasts 2
17 germ cell progenitors
32 goblet cells
21 otf+ cells 2
neurons
0 ChAT neurons
10 GABA neurons
38 npp18+ neurons 2
33 epidermis DVb
phagocytes
9 phagocyte progenitors
14 phagocytes
22 psd+ cells
16 otf+ cells 1
25 protonephridia flame cells
34 protonephridia tubule cells
20 goblet cell progenitors
13 pharynx cell type
23 pharynx cell type progenitors
secretory cells
19 secretory 1
26 secretory 2
27 secretory 3
28 secretory 4
29 secretory 5
muscle
3 muscle body 1
6 muscle pharynx
11 muscle body 2
parenchymal cells
4 pgrn+ parenchymal cells
18 psap+ parenchymal cells
36 aqp+ parenchymal cells
UMAP 1
UMAP 2
not transferred
15 neoblasts 3 30 serotonin neurons 31 npp-18+ neurons 1
35 secretory 6 37 cav-1+ neurons 39 eye-53 neurons 40 neoblast 4
C
Trypsin-dissociated dataset (21,610 cells)
"Germ cell progenitors"
Labels transferred from this study
17 germ cell progenitors
D
Smed-eIF3c (HM055595)
SmMSTRG.398
Smed-nanos (EF035555)
SmMSTRG.6394
0
1
2
3
E Data Integration Labels transferred from Plass et al.
parenchymal cells
9 parenchymal progenitors
13 psap+ parenchymal cells
18 pgrn+ parenchymal cells
21 pigment cells
24 aqp+ parenchymal cells
35 ldlrr+ parenchymal cells
29 otf+ cells 1
neoblasts
0 neoblasts 1
1 neoblasts 2
4 neoblasts 3
6 neoblasts 4
12 neoblasts 5
17 neoblasts 6
25 neoblasts 7
33 neoblasts 8
muscle
7 muscle body
8 muscle progenitors
19 muscle body 2
neurons
5 neural progenitors
11 ChAT neurons 1
16 GABA neurons
23 ChAT neurons 2
phagocytes
14 phagocyte progenitors
27 phagocytes
34 psd+ cells
28 otf+ cells 2
20 goblet cells progenitors
31 protonephridia
secretory cells
15 secretory 1
32 secretory 2
36 secretory 3
37 goblet cells
26 pharynx cell type
epidermis
2 early epidermal progenitors 2
3 early epidermal progenitors 1
10 late epidermal progenitors
22 epidermis
30 epidermis DVb
UMAP 1
UMAP 2
