# Supplementary material for: ACME dissociation: a versatile cell fixation-dissociation method for single-cell transcriptomics
Source: Genome Biol. 2021 Apr 8;22:89. doi: 10.1186/s13059-021-02302-5 (PMC8028764; doi:10.1186/s13059-021-02302-5)
Supplement: Supplementary file 6 — Additional file 6:. Review history. [file 13059_2021_2302_MOESM6_ESM.docx]

Review History

**First round of review**

**Reviewer 1**

**Are you able to assess all statistics in the manuscript, including the appropriateness of statistical tests used?**

Yes

**Comments to author:**

This study by Garcia-Castro et al. introduces a novel dissociation and fixation method which is compatible with single-cell RNA-seq by a modified version of a published plate-based combinatorial barcoding protocol (SPLiT-Seq). The authors demonstrate that the ACME method permits the isolation and repeated cryopreservation of cells without substantial loss of RNA quality and preserved flow cytometry profiles. To demonstrate the power of ACME in combination with their adjusted SPLiT-seq protocol, they perform a proof-of-principle experiment, sequencing ~30,000 cells from two planarian species in a single experiment. By a comparative analysis with data from a previously published whole planarian Drop-seq experiment, they show that the ACME/SPLiT-seq approach permits sequencing of all major cell types.

ACME is a potentially very useful method for the community as it facilitates tissue dissociation and immediate fixation and therefore may avoid the requirement of minimizing the time between cell isolation and sequencing library preparation. However, I feel that the authors need to analyze more thoroughly whether or not their method introduces technical artefacts and whether it preserves the complexity of single-cell transcriptomes. Moreover, it would significantly increase the value of the study if the compatibility of the method with human or mouse primary tissue cells could be demonstrated, since isolation of such cells without immediate fixation frequently results in dissociation- and processing-related alterations of the transcriptome.

Specific points:

1. It is not entirely clear to me how the FACS gates were defined in Figure 1. Is it correct that cells are first gated based on FSC (Figure 1C), and then on DRAQ5 (Figure 1D) to obtain the populations show in Figure 1E. It is also unclear to me how the DRAQ5 gate is defined (Figure 1D). More detailed explanation should be included.

2. Please show a similar FACS analysis for ACME isolation of mammalian cells, i.e., mouse or human primary tissue. Since human and mouse tissue are major targets of single-cell RNA-seq analyses, it would be important to demonstrate the suitability of ACME for such cells. Please also include a FACS plot for the ungated populations shown in Figure 2 to demonstrate that the FACS strategy selects the desired populations.

3. The authors demonstrate that flow cytometry profiles and RNA integrity are stable during multiple rounds of cryopreservation. Although the presented analysis provides an indication that the transcriptome could also be stable during cryopreservation, the authors should directly test this by single-cell RNA-seq of cells that have undergone different number of freeze/thaw cycles and characterize the resulting transcriptome changes.

4. Please comment on the suitability of ACME for other commonly used single-cell RNA-seq protocols. Would it be compatible with widely used droplet microfluidics-based protocols such as 10x?

5. To identify and exclude multiplets, cells with >5000 UMI counts are discarded. The authors should report the fraction of events accounted for by these multiplets as this provides a direct test of their gating strategy (Figure 1) for excluding multiplets.

6. If downsampled to the same number of reads per cell, how does the number of quantified genes compare between the ACME/SPLiT-seq approach and the Drop-seq method of FACS sorted live cells applied in Plass et al., 2018, Science? Are these methods comparable in terms of complexity and quality of single-cell transcriptomes? Please also provide a direct comparison of single-cell transcriptome data generated by the two approaches for the same cell type. It would be important to characterize the differences between the methods, e.g., does ACME allow an improved detection of lowly expressed genes due to better preservation of RNA? Can gene expression differences be inferred which are linked to technical differences in terms of dissociation, processing and sequencing (compare Denisenko et al., 2020, Genome Biology)?

7. For some cell types the authors observe a lack of resolution with their method compared to their previous data in Plass et al., e.g., for parenchymal cells. To test if these cell types are indeed not discernable (or if this is just a question of cluster resolution), the authors should test a supervised classification of the cell types in their new dataset using their previous data as training set. A number of methods have been published for this task, e.g. Kiselev, 2018, Nature Methods or Pliner et al., 2019, Nature Methods. This should equally be done in the other direction as well, i.e., using the novel data as training set to annotate cell types in Plass et al. In particular, this could show whether the novel nanos-positive germ cell precursor state can be identified in the Drop-seq data or not. In case it is there, a comparative analysis should be performed in order to investigate the differences arising from the two protocols which might explain why they could not be resolved by clustering in Plass et al.

8. To identify corresponding cell types between the two planarian species the authors should also test available computational methods for cell type annotation, using one species as training set to classify cell types of the other (see point 7.).

9. The authors should use their proof-of-principle experiment to better demonstrate the benefit of their approach, i.e., the characterization of evolutionary changes of particular cell types between the two species, which should ideally not be convoluted by technical effects avoided by the early fixation and common processing in a single experiment. They should attempt this for an abundant cell types such as the neoblast, which is of core interest to the community as an adult pluripotent stem cell, and for a rare cell types with only a small number of sequenced cells.

**Reviewer 2**

**Are you able to assess all statistics in the manuscript, including the appropriateness of statistical tests used?**

No

**Comments to author:**

The author presented ACME, a fixation/dissociation method called maceration, that is compatible with single-cell sequencing. The advantage of this method, as the authors claim, is that it allows preservation of RNA and cell integrity such that high quality results can be obtained from fixed cells. Secondly, they also claim that this method mitigates artefacts of dissociation since it dissociates and fixes at the same time. While this all sounds promising, no comparative studies were performed and thus, this reviewer find that the claims are not justified by the evidence.

Major comments

1. While the authors show dissociation into single cells of multiple organisms, only Planarian single-cell RNA sequencing data were presented. These data do not justify the universality of ACME for scRNA-seq.

2. A large amount of aggregates is formed in ACME (>50%?). This is evidence that dissociation is not very good using this method, and may affect downstream cell representation (as claimed by the authors to be superior in Figure 5). No comparison of single cell dissociation ability with other methods such as Trypsin/collagenase, etc.

3. The amount of UMI detected per cell is < 1000. This is very low information-content data. Yes, this may depend on organisms and cell type, I understand that. However, this low information content data can also arise from bad dissociation/preservation leading to poor quality cells. Most studies today report ~8000 UMIs per cell on fresh cells. The authors should be able to be close to this benchmark either using a different organism such as mammalian cells or a different platform such as 10X.

4. With regards to data quality, the authors make the comparison that the UMI per cell detected with ACME dissociation/fixation followed by SPLiT-Seq is comparable to prior results found by sci-RNA-seq3. This comparison cannot be made unless samples are sequenced to a similar read depth. At the very least, data from the libraries sequenced to the higher read depth should be down-sampled to the read depth of the sample with lower read depth. In the case of the study the authors cite here that used sci-RNA-seq3 the raw read depth was 5000 raw reads per cell. However, in the ACME data the total raw reads were >1.3 billion with 33827 cells recovered (from both species) meaning the raw read depth was ~40,000 read pairs per cell. Despite being sequenced 8x more, the ACME data only recovered ~1.5x the number of UMI's per cell, which again is poor. The authors either need to down-sample before attempting to make this comparison or remove this comparison. Similarly, the authors also make the comparison that the number of UMI's per cell they detect with ACME is similar to the original SPLiT-Seq paper. After quickly glancing over it, the only numbers I can find reported in the SPLiT-Seq paper are for libraries at saturated read depth (with the exception of one instance in which the value for UMI per nucleus are similar to what is presented here when the nuclei are sequenced to ~1/3 the read depth). These are apples to oranges comparisons and either should not be included or should be redone so they are comparing with both equal read depths and with cells (not nuclei). Furthermore, the cells used for the comparison should be from the same organism.

5. It would be helpful for the authors to provide RIN values alongside the bioA results in figure 3G-K. It is claimed that the "Keeping cells on ice effectively prevents this effect", however it is evident from figure 3 that degradation (although slow) still occurs. Adding the RIN values would help to more definitively clarify this. Furthermore, from what this reviewer can tell it appears that no replicates were used for this part of the experiment. The number of replicates should be included. It also does not appear that methods for these tests were included in the methods section. They need to be included.

6. No comparative analysis was done for the above with other dissociation/fixation methods. To justify the authors' claim, they have to show that RNA quality is better maintained in comparison to other dissociation-fixation methods such as PFA in SPLIT-seq or fresh dissociation methods such as Trypsin.

7. Please demonstrate that ACME is compatible with other sequencing platforms as suggested in the discussion. It is a very narrow application to be focused on planarians and SPLIT-seq if this is a manuscript claiming adaptability of the method.

8. As a far as this reviewer can tell, no supp. data show that the scRNA-seq experiments have been repeated (n=1 experiment). It has not been shown that this method can give good results consistently.

9. What is the novel application of this method? SPLIT-seq already utilizes fixation of cells and it is apparent that PFA is a standard fixation method, so fixation cannot be the novel factor here. I suppose the novelty could be the handling artefacts introduced during dissociation. The authors spend an entire paragraph in the background section going over how other methods for dissociating and processing cells can result changes in cell's gene expression patterns and thus there is a need for simultaneous dissociation and fixation. This claim is then echoed in the results section where the authors state that the decoupled enzymatic dissociation followed by processing or cryopreservation will result in biological effects that can be observed due to cells being out of their cellular context for several hours. The authors then claim that ACME "solves this problem" without showing that cells are processed quickly enough that their transcriptomic profiles do not change. This is again restated in the discussion section: "ACME is a cell dissociation approach that fixes cells while they are being dissociated… disrupting the cellular environment has effects on cellular transcriptomic profiles." However, the authors do not provide any evidence that their method does not also result in changes (or even minimizes changes) in cells' transcriptomic profile. What the authors do show is that they detect the same cell types as in prior bodies of work, not changes in cell state. The authors also show bioA plots of RNA. This does not constitute validation that the transcriptomic profile is not changing as it is still unknown if the same transcripts are being expressed or present in similar quantities to pre-ACME treatment or if they are different transcripts that happen to be similar in size. Analysis needs to be conducted on potential changes in cell states (aka validate with non-dissociation-based methods).

10. The author demonstrate that the composition obtained via ACME is different (better) using scRNA-seq than standard dissociation approaches. This can arise from an aggregate problem as stated above. These data should be validated with non-dissociation-based methods such as IF microscopy.

11. It is this reviewer's opinion that if the authors are going to spend a paragraph in the results going over RNA integrity during ACME and then a full paragraph in the methods on how the RNase activity of the BSA used will affect the results, then they should include the vendor and catalog number of the BSA used. For any new methods paper including information on what reagents were sourced from where would be important to any lab looking to implement ACME in their own research settings. This information would also be good to include for all other reagents necessary to the ACME fixation/dissociation step.

12. This original SPLiT-Seq protocol had a mixing rate of 0.1% (https://science.sciencemag.org/content/360/6385/176.long) on unfixed cells. The ACME method appears to result in higher (2.8%) levels of mixing. If the cells are fixed, how has the sample mixing rate increased by over 20x?

Minor:

1. In providing background (paragraph 4) into how maceration with ACME has previously been used to produce fixed cells, it would be helpful for the authors to include a brief explanation on how the acetic acid, methanol, and glycerol works to dissociate soft tissues.

**Reviewer 3:**

**Are you able to assess all statistics in the manuscript, including the appropriateness of statistical tests used?**

No

**Comments to author:**

In the manuscript by Garcia-Castro, et al., the authors describe a new method for performing single cell sequencing on fixed cells, which they term ACME. ACME is a modification of an older dissociation/fixation method and then adapted to single-cell sequencing technology. The authors demonstrate that their technique holds up against previously published data in planarians, in addition to showing that it can be used for preserving samples in the field. It is likely that this technique will be of high interest to the single-cell genomics community. The data are of high quality and mostly well-controlled. I believe that the manuscript needs a bit more work before publication in Genome Biology.

Major points

1. In Figure 1B-F, it would be helpful to include ACME dissociated cells stained with Hoechst to demonstrate the spread of cells by Hoechst red vs Hoechst blue fluorescence, as is commonly done in the planarian field. In B, E and F, the G1 and G2 populations appear as clearly separated populations, but cells in S (which should be visible between the G1 and G2 populations) appear to be missing. Including a Hoechst plot would increase confidence that the ACME treatment doesn't substantially alter the expected spread of cells. Similarly, including a flow plot for ACME treated cells from lethally irradiated animals would be useful for confirming the cell cycle status of these populations (the G2 population should be gone by 1-2 days post-irradiation).

2. In Figure 3G-K, the effects of the different treatments on the 28S and 18S RNAs are clear; however, these RNAs are much more abundant than the mRNAs we want to isolate for sequencing. So while these highly abundant RNAs survive these challenges (with some degradation), this does not necessarily mean the mRNA profiles of the cells are acceptable for sequencing or reflective of the live state. Thus, a quantitative measure of RNA integrity via bioAnalyzer traces and RNA Integrity Numbers (RIN) should be provided.

3. A head to head comparison of ACME with other dissociation/fixation protocols would be useful to determine whether this method outperforms other methods (for example, by comparing flow plots, bioAnalyzer traces, RIN).

4. As split-seq has not yet been performed in planarians, the authors should strongly consider adding to figure 2 the ability to correctly resolve cell types from diverse animals - not just 2 species of planarians. Overall Fig 2 is quite thin and needs more data than just dissociation and flow plots.

Minor points

1. In Figure 3F, population percentages should be provided (as in B-E) to demonstrate quantitatively whether one population is more sensitive to freeze/thaw cycles than the other.

2. Is there an explanation for why sub-library 3 had so few cells pass the thresholds despite having similar read depth?

3. In Figure S2, showing the whole UMAP plot in each panel would make it easier to understand the relationships of the clusters.

4. In fig 3B - why is there considerably less debris after freeze-thawing? Are there additional washes that take away debris? One would predict that more freeze-thawing would yield more debris.

5. Page 9, lines 9-20. You state several times that the japonica genome assembly is worse, so that hinders your sequence alignments to this species. Did you try the transcriptomes of both species instead? I believe those are of quite high quality on the planmine database.

6. Consider a new results section for Fig 5 on its own to help readability.

7. Page 9, line 43 "Smedwi-1" should be all small letters.

8. The authors should consider a more in depth analysis of the germ cell precursors since this is the first study to detect them.

9. It is unclear in Fig 1B why presumably larger cells (higher Con-A) with the same DNA content are not included in the G2 and G1 gates. Those cells at the same DNA level shouldn't be aggregates, so what are the criteria for excluding them?

**Authors Response**

**Point-by-point responses to the reviewers’ comments:**

Reviewer #1: This study by Garcia-Castro et al. introduces a novel dissociation and fixation method which is compatible with single-cell RNA-seq by a modified version of a published plate-based combinatorial barcoding protocol (SPLiT-Seq). The authors demonstrate that the ACME method permits the isolation and repeated cryopreservation of cells without substantial loss of RNA quality and preserved flow cytometry profiles. To demonstrate the power of ACME in combination with their adjusted SPLiT-seq protocol, they perform a proof-of-principle experiment, sequencing ~30,000 cells from two planarian species in a single experiment. By a comparative analysis with data from a previously published whole planarian Drop-seq experiment, they show that the ACME/SPLiT-seq approach permits sequencing of all major cell types.

ACME is a potentially very useful method for the community as it facilitates tissue dissociation and immediate fixation and therefore may avoid the requirement of minimizing the time between cell isolation and sequencing library preparation. However, I feel that the authors need to analyze more thoroughly whether or not their method introduces technical artefacts and whether it preserves the complexity of single-cell transcriptomes. Moreover, it would significantly increase the value of the study if the compatibility of the method with human or mouse primary tissue cells could be demonstrated, since isolation of such cells without immediate fixation frequently results in dissociation- and processing-related alterations of the transcriptome.

*We thank the reviewer for these comments. We hope that the reviewer finds these sufficiently addressed in the revised version. In particular we have a) compared ACME to Trypsin dissociations at the cytometric level, b) performed careful comparisons between our ACME dissociated cell dataset with previous studies that used Trypsin dissociation and c) provide evidence that mouse embryos can be dissociated with ACME.*

Specific points:

1.	It is not entirely clear to me how the FACS gates were defined in Figure 1. Is it correct that cells are first gated based on FSC (Figure 1C), and then on DRAQ5 (Figure 1D) to obtain the populations show in Figure 1E. It is also unclear to me how the DRAQ5 gate is defined (Figure 1D). More detailed explanation should be included.

*We thank the reviewer for this comment. Since the way we gate singlets is relatively standard in the cell cytometry field we originally thought that further explanation was unnecessary; we apologize for this omission. We have added the following explanation: “To distinguish singlets from doublets and other aggregates we use a singlet filter: aggregates are gated out by their increased area signal compared to the height. This can be achieved using either the FSC (Figure 1C) or the DRAQ5 (Figure 1D) area vs height signal, or both, by selecting events with well-correlated signal area and height values. Then, gating DRAQ5 positive cells (DRAQ5 area vs FSC area) excludes cellular debris to obtain clear G1 and G2 populations (Figure 1E).” We also included the sentence “Singlets are selected based on their well correlated signal area vs height, while aggregates display high area vs height ratios” in the figure legend and added clarifications in the panels.*

2.	Please show a similar FACS analysis for ACME isolation of mammalian cells, i.e., mouse or human primary tissue. Since human and mouse tissue are major targets of single-cell RNA-seq analyses, it would be important to demonstrate the suitability of ACME for such cells.

*We have now added a cell cytometry analysis of mouse embryos (E11.5). This shows that mammalian cells can be dissociated using ACME.*

Please also include a FACS plot for the ungated populations shown in Figure 2 to demonstrate that the FACS strategy selects the desired populations.

*Following the reviewer’s suggestion, we have now added the ungated populations, with a subset logarithmic scale to aid visualisation.*

3.	The authors demonstrate that flow cytometry profiles and RNA integrity are stable during multiple rounds of cryopreservation. Although the presented analysis provides an indication that the transcriptome could also be stable during cryopreservation, the authors should directly test this by single-cell RNA-seq of cells that have undergone different number of freeze/thaw cycles and characterize the resulting transcriptome changes.

*We understand the concern raised by the referee, but considering the costs of iterative single-cell experiments, we consider this is out of the scope of the paper. In this sense, it is important to consider that: a) We perform up to five cycles of freezing for demonstration purposes, but as we mention in the text, two cycles or less suffice for most applications. Therefore, we performed our planarian SPLiT-seq experiment with that number of cycles. b) We demonstrate that the diminished RNA integrity is always far superior to that obtained with formaldehyde, so our method will in any case be a major improvement with respect to the current state of the art. Therefore, since the original SPLiT-seq paper used formaldehyde, a fixative that affects RNA integrity in a far more dramatic way, we believe that it is unlikely that the slight decreases in RNA integrity observed in our method induce large transcriptome changes.*

4.	Please comment on the suitability of ACME for other commonly used single-cell RNA-seq protocols. Would it be compatible with widely used droplet microfluidics-based protocols such as 10x?

*To directly address this point, we have generated a 10X Genomics dataset using ACME dissociated cells from the cnidarian Nematostella vectensis. We present these results in a new main figure (Figure 4). This dataset recapitulates the previous analysis done by Arnau Sebé-Pedrós and co¬workers (Cell 2018). This shows that ACME is compatible with microfluidics-based protocols, as well as providing an additional application in a completely different model species.*

5.	To identify and exclude multiplets, cells with >5000 UMI counts are discarded. The authors should report the fraction of events accounted for by these multiplets as this provides a direct test of their gating strategy (Figure 1) for excluding multiplets.

*Removing the highest UMI containing cell barcodes was done to prevent two types of “multiplets”. This could be aggregates resulting from the dissociation and not discarded by the FACS sorting, but also collisions (cells sharing the same cell barcode). Both can translate into high UMI cell barcodes. We estimate the collision rate with the mixed species experiment, but this only identifies Schmidtea-Dugesia collisions. Schmidtea-Schmidtea and Dugesia-Dugesia collisions must occur as well. We further refined our analysis using Doublet Decon to remove further doublets. Taking these considerations into account, it is not possible to relate the number of cells above 5000 UMIs with the aggregates after the gating strategy and FACS sorting, as these are mixed with the “collisions”. We have carefully phrased this section, added the Doublet Decon description. The section describing the >5000 UMI exclusion now reads “We also discarded very high UMI containing cell barcodes (>5000 counts) to prevent the inclusion of aggregates that have remained after FACS purification as well as collisions, cells sharing the same cell barcode (234 and 177 cell barcodes excluded in S. mediterranea and D. japonica respectively)” and the section that describes the Doublet Decon removal of within species collisions now reads “To establish cell types and abundances in both species we further analysed our sets of 19,741 and 14,086 cells. To minimise the inclusion of Schmidtea-Schmidtea and Dugesia-Dugesia doublets we used DoubletDecon (58), which resulted in further refined datasets of 19,025 and 13,406 cells”*

6.	If downsampled to the same number of reads per cell, how does the number of quantified genes compare between the ACME/SPLiT-seq approach and the Drop-seq method of FACS sorted live cells applied in Plass et al., 2018, Science? Are these methods comparable in terms of complexity and quality of single-cell transcriptomes? Please also provide a direct comparison of single-cell transcriptome data generated by the two approaches for the same cell type. It would be important to characterize the differences between the methods, e.g., does ACME allow an improved detection of lowly expressed genes due to better preservation of RNA? Can gene expression differences be inferred which are linked to technical differences in terms of dissociation, processing and sequencing (compare Denisenko et al., 2020, Genome Biology)?

*We have now thoroughly compared our dataset with Plass et al. Science 2018, a dataset obtained using trypsin dissociation and Drop-seq. This has a UMI per cell distribution similar to ours, and similar cell numbers, and therefore we did not attempt to downsample. This would have been troublesome since the datasets were generated with different sequencing technologies and read lengths. We reanalysed the Plass et al. dataset using our improved genome annotation. These comparisons are presented in a new main figure (Figure 8), and a supplementary figure (S7-8). These comparisons revealed that cell types can be robustly profiled by both methods. As a result of this, we managed to integrate both datasets using Seurat integration algorithms. Unfortunately, comparisons at the differential gene expression analysis level proved to be difficult, as these are confounded by the multiple differences of the 2 datasets. These differ not only in dissociation method, but, most importantly, in single cell transcriptomic platform (Drop-seq vs. SPLiT-seq) and others (NextSeq vs. NovaSeq sequencing technology, read length, etc). We present these results in the new Figure 8 and Supplementary Figure 7.*

*For the reviewer’s information we would like to present some of our results here:*

*-First of all, we recapitulated analyses already performed in Plass et al. to show that our reanalysis of that dataset still recovers those:*

*In this comparison (Figure R1, top panel), equivalent to the one shown in Supplementary Figure S15 of Plass et al. Science 2018, we analyse differences within two different libraries of the Plass et al. dataset, comparing days 0 vs. days 2 and 4 of regeneration, and recovers similar genes, including runt-1, a gene that had been shown to be activated in regeneration conditions previously.*

*-We then went on to compare samples between datasets (Figure R1, middle panel):*

*In the comparison of all genes of Plass et al. vs all genes of our dataset, the distribution of log fold changes is heavily affected by the dataset type. Log fold changes lean towards the upregulation in Plass et al., which could point to an artefact where more genes are detected in the Plass et al. dataset. This could be because of the differences in dissociation (trypsin vs. ACME) but could as well be because of the differences in the single-cell transcriptomic platform (Drop-seq vs SPLiT-seq). In this plot we have highlighted differentially expressed genes as detected using Seurat as well as heat shock proteins in planarians. Importantly, we have curated a list of genes known to be upregulated in stress (Known Stress Complement) by searching for bona fide homologues of the genes in the set of 17 stress-response-related genes (Fosb, Fos, Jun, Junb, Jund, Atf3, Egr1, Hspa1a, Hspa1b, Hsp90ab1, Hspa8, Hspb1, Ier3, Ier2, Btg1, Btg2, Dusp1) described in Denisenko et al. Genome Biology 2020 as suggested by the reviewer. In these conditions, though, it is impossible to assess the significance of these changes.*

*-To elucidate in cell type composition changes between datasets affect the comparison, we compared only neoblasts clusters between the Plass et al. dataset and our dataset (Figure R1 bottom panel). In this comparison, it became evident that a group of genes (upper left side of the plot) are detected in neoblasts only in the Plass et al. dataset and not in our dataset. This has two possible interpretations: on one hand, this could reveal that the trypsin/Drop-seq approach is more sensitive, and manages to measure the expression of these genes despite having similar UMI contents. On the other hand, these could also arise from an increased specificity of the ACME/SPLiT-seq approach. Again, this could be because of the dissociation method or the single cell transcriptomic platform. Altogether, we think that these comparisons are very difficult and confounded by many factors, but wanted to present them here for the sake of the discussion raised by the reviewer.*

7. For some cell types the authors observe a lack of resolution with their method compared to their previous data in Plass et al., e.g., for parenchymal cells. To test if these cell types are indeed not discernable (or if this is just a question of cluster resolution), the authors should test a supervised classification of the cell types in their new dataset using their previous data as training set. A number of methods have been published for this task, e.g. Kiselev, 2018, Nature Methods or Pliner et al., 2019, Nature Methods. This should equally be done in the other direction as well, i.e., using the novel data as training set to annotate cell types in Plass et al. In particular, this could show whether the novel nanos-positive germ cell precursor state can be identified in the Drop-seq data or not. In case it is there, a comparative analysis should be performed in order to investigate the differences arising from the two protocols which might explain why they could not be resolved by clustering in Plass et al.

*Following the reviewers suggestion we have now performed a series of new analyses, presented at full in a new figure (Figure 8) and its accompanying supplement (Supplementary Figure 7). We first reanalysed the dataset to rule out differences in resolution coming from our improved gene annotation, as Plass et al mapped their reads to the transcriptome. We then used the Seurat Transfer labels function to find equivalent cells across cell types. We find that cluster size and clustering resolution affects the clustering of individual cell types, but all major cell types are robustly recovered by both approaches. Only very minor clusters (0.2-1.8% of total cells) cannot be transferred from one dataset to another, which is likely caused by their low frequency. The trypsin dataset has increased resolution of parenchymal cells, but we do find equivalents, and conversely, we find more secretory types. Most likely, these differences are a question of cluster resolution, as the reviewer states. We have made this possibility explicit in the text: “Only 3 clusters from the Plass et al. dataset did not possess equivalents in our dataset: the two smallest neoblast clusters and the goblet cell progenitors. These clusters contain low cell numbers (0.4-1.6%). We therefore found equivalents for clusters representing ~97% of cells of the trypsin-generated dataset. When we performed the reverse comparison (Supplementary Figure 8A) a larger number of clusters from our dataset were not identified in the trypsin-generated dataset (Supplementary Figure 8B). These were also minor clusters (0.2-1.8%) and included novel neuron types such as the serotonin and eye-53+ neurons. This suggests that most differences in cell type clustering can be explained by cluster size and resolution parameters, and both datasets contain broadly comparable cell types”*

*Regarding the nanos-positive germ cell progenitors, we think that it is a different case. For instance, we do not cluster out pigment cells, but when we examine a marker of pigment cells from the trypsin-dissociated dataset (Supplementary figure S5) we see that this is distributed in a region of a parent cluster in the ACME dissociated dataset, and the labels are also transferred to that region. This indicates that this is a question of cluster resolution, and increasing the cell number would likely make the pigment cells cluster out using ACME. In the integration, there is a pigment cell cluster containing cells from both sources, which can be interpreted as the pigment cells in Plass et al. aiding the clustering of pigment cells in the ACME-generated dataset. However, the germ cell precursors are different. When we examine markers in Plass et al., Fincher et al. and Zeng et al. (Supplementary Figure S6) these cells are interspersed along the neoblast clusters in all three cases, not showing any clustering. This is paralleled in the transfer labels comparison (Supplementary Figure S5). Importantly, in the integration, the germ cell progenitor cluster is lost. This could be interpreted as the germ cell precursors in the ACME-generated dataset not being enough to aid the clustering in the other dataset, contrary to what happened with the pigment cells. This has to be taken together with the two other facts exposed in the original paper: 1) Our D. japonica ACME-generated dataset, with fewer cells and arguably worst genome annotation retrieves a germ cell progenitor cluster. 2) Neither Fincher et al nor Zeng et al retrieved a germ cell progenitor cluster, despite the fact that both studies have a higher number of neoblasts profiled and higher UMI contents. Our re-analysis of Plass et al. does not find this cluster either and, again, marker expressing cells are found interspersed along the neoblast clusters. This rules out that the clustering of the germ cell progenitors is a consequence of our novel improved gene annotation. Altogether, these facts strongly suggest that the case of the germ cell precursors cannot be explained as a question of cluster resolution. In summary, no analysis of trypsin-dissociated cells retrieves the germ cell progenitors, or even shows some partial clustering, while all of our analyses of ACME-dissociated cell do, in different conditions – except when ACME-dissociated cells are integrated with trypsin-dissociated cells. We believe that these facts are worth noting in the text, as they can foster further studies about the biology of these germ cell progenitors and their differential detection using ACME and trypsin dissociations. In that section we have noted: “However, the germ cell progenitor cluster of our ACME-generated dataset was transferred to the neoblast clusters of Plass et al (Supplementary Figure 8C). Similarly, markers of the germ cell progenitors were also found interspersed among the neoblast clusters in our analysis of the Plass et al. dataset (Supplementary Figure 8D). This supports the claim that the clustering of germ cell precursors in our dataset depends on ACME dissociation, as none of the trypsin-based analyses shown here or previous papers retrieves them”*

8.	To identify corresponding cell types between the two planarian species the authors should also test available computational methods for cell type annotation, using one species as training set to classify cell types of the other (see point 7.).

*While we think this is a very interesting topic and we thank the reviewer for the suggestion, we believe this comparison falls beyond the scope of this work, since our manuscript focuses on ACME, a cell dissociation-fixation approach for single-cell transcriptomics. As the reviewer mentions, several computational methods are available, but this is still an area of active development. These have been used before typically with tissues consisting of several cell types, but to our knowledge they have not been put to the test of whole organisms with the complexity of very different cell types. We have attempted several of these approaches. However, the time provided for this revision does not allow us to properly benchmark these methods and arrive to a solid answer. We are of the opinion that optimising these methods for such complex datasets could be subject of a monographic paper on its own, and we certainly will aim at this in the future.*

9.	The authors should use their proof-of-principle experiment to better demonstrate the benefit of their approach, i.e., the characterization of evolutionary changes of particular cell types between the two species, which should ideally not be convoluted by technical effects avoided by the early fixation and common processing in a single experiment. They should attempt this for an abundant cell types such as the neoblast, which is of core interest to the community as an adult pluripotent stem cell, and for a rare cell types with only a small number of sequenced cells.

*We share with the reviewer the excitement for such comparisons, and this will clearly be one of the major lines of our future research. However, given the time constraints for the revision of this paper and the technical nature of our method paper, we think this is beyond the scope of our paper. We look forward to performing these interesting analyses in the near future.*

Reviewer #2: The author presented ACME, a fixation/dissociation method called maceration, that is compatible with single-cell sequencing. The advantage of this method, as the authors claim, is that it allows preservation of RNA and cell integrity such that high quality results can be obtained from fixed cells. Secondly, they also claim that this method mitigates artefacts of dissociation since it dissociates and fixes at the same time. While this all sounds promising, no comparative studies were performed and thus, this reviewer find that the claims are not justified by the evidence.

*We thank the reviewer for these comments, and we would like to clarify some of our claims, to avoid potential misunderstandings. We hope that the revised version, as well as this response, convince the reviewer of the following points:*

*a)	the high level of preservation of RNA integrity of ACME compared to formaldehyde fixation, which is the current standard fixation method for SPLiT-seq.*

*b)	we originally did not directly claim that our method “mitigates” the artefacts of trypsin dissociation. Some authors have shown stress and other effects of enzymatic dissociation, and we suggested that our method would, in theory, not induce those (as there is no enzymatic dissociation). However, we agree with the reviewer that this is a key hypothesis to assess and a comparison is needed. Therefore, we have now compared our Schmidtea mediterranea dataset to previous trypsin-based single cell data. This is a difficult task – as the reviewer warns – since these are done not only with different dissociation methods but also different single cell transcriptomic platforms (and in different labs, in different batches, with different planarian populations, etc). However, our analysis shows that the data can be broadly compared and integrated. Unfortunately, differences at the gene expression level are difficult to pinpoint with such technical differences. However, we believe that our analyses show that ACME can be used to obtain full cell type atlas of organisms using different single cell transcriptomic approaches, and it does not require lengthy enzymatic incubations which have been shown by other authors to induce stress effects.*

Major comments

1.	While the authors show dissociation into single cells of multiple organisms, only Planarian single-cell RNA sequencing data were presented. These data do not justify the universality of ACME for scRNA-seq.

*In the revised version, we have added Nematostella vectensis, a third species, from a second phylum, and with a different single-cell sequencing approach. However, we would like to clarify that we do not believe (and claim) that ACME is “universal”, but that it is highly “versatile”. We thus apologize for this potential misunderstanding. We believe that the claim of versatility is fully justified, as we manage to dissociate 9 animal species from different branches of the tree of life, at different developmental stages, and from a broad range of habitats, and sequence three of these, with two single-cell transcriptomic approaches.*

2.	A large amount of aggregates is formed in ACME (>50%?). This is evidence that dissociation is not very good using this method, and may affect downstream cell representation (as claimed by the authors to be superior in Figure 5). No comparison of single cell dissociation ability with other methods such as Trypsin/collagenase, etc.

*In the revised version we provide cytometry plots using Trypsin vs ACME (Figure S1). ACME results in more aggregates than trypsin, but, conversely, trypsin seems to generate far larger amounts of cell debris. In fact, the reviewer is right about ACME but should think about enzymatic dissociations in a similar way: both trypsin and ACME might affect the representation of cell types, and this has been so far given very little attention. We did not claim that cell representation is superior using ACME in the original manuscript. Ultimately, no cell dissociation method will generate only single cells without aggregates and/or debris. The levels of aggregates/debris are likely to be affected by experimental conditions, and will likely vary in each different organism. Our comparison of ACME dissociated cells and Trypsin dissociated cells (Figure S1) will contribute to highlighting the differences between these two approaches and help other researchers chose their preferred method. Ultimately, our analysis will bring attention to this subject. We also discuss this in the main text: “Compared to Trypsin dissociations performed as previously described (Hayashi), ACME dissociations show more aggregates and less cellular debris (Figure S1A). Ultimately, all dissociation methods generate variable amounts of aggregates and debris, but these can be excluded due to their cytometric profiles”*

3.	The amount of UMI detected per cell is < 1000. This is very low information-content data. Yes, this may depend on organisms and cell type, I understand that. However, this low information content data can also arise from bad dissociation/preservation leading to poor quality cells. Most studies today report ~8000 UMIs per cell on fresh cells. The authors should be able to be close to this benchmark either using a different organism such as mammalian cells or a different platform such as 10X.

*We have now obtained single-cell transcriptomic data of the cnidarian Nematostella vectensis, using 10X Genomics and ACME dissociated cells. The UMI content of this dataset is, as in the case of planarians, very similar to that obtained by Sebé-Pedrós and co-workers (Cell 2018) in the same organism, using enzymatic dissociation and MARS-seq. This strongly indicates that the UMI content may significantly depend on the organism, as the reviewer acknowledges. It is highly possible that other factors affect this, and this is still very highly debated in the field. We also show that ACME provides a much higher RNA integrity than formaldehyde. Therefore, we believe that the low UMI content can arise partially from the SPLiT-seq technique that it is still at its infancy. Further improvements of the experimental single-cell transcriptomic technique can therefore raise this, but our manuscript focuses on ACME, a cell dissociation method, not on the optimisation of the SPLiT-seq single-cell transcriptomic technique.*

4.	With regards to data quality, the authors make the comparison that the UMI per cell detected with ACME dissociation/fixation followed by SPLiT-Seq is comparable to prior results found by sci-RNA-seq3. This comparison cannot be made unless samples are sequenced to a similar read depth. At the very least, data from the libraries sequenced to the higher read depth should be down-sampled to the read depth of the sample with lower read depth. In the case of the study the authors cite here that used sci-RNA-seq3 the raw read depth was 5000 raw reads per cell. However, in the ACME data the total raw reads were >1.3 billion with 33827 cells recovered (from both species) meaning the raw read depth was ~40,000 read pairs per cell. Despite being sequenced 8x more, the ACME data only recovered ~1.5x the number of UMI's per cell, which again is poor. The authors either need to down-sample before attempting to make this comparison or remove this comparison.

Similarly, the authors also make the comparison that the number of UMI's per cell they detect with ACME is similar to the original SPLiT-Seq paper. After quickly glancing over it, the only numbers I can find reported in the SPLiT-Seq paper are for libraries at saturated read depth (with the exception of one instance in which the value for UMI per nucleus are similar to what is presented here when the nuclei are sequenced to ~1/3 the read depth). These are apples to oranges comparisons and either should not be included or should be redone so they are comparing with both equal read depths and with cells (not nuclei). Furthermore, the cells used for the comparison should be from the same organism.

*We agree with the reviewer that these types of comparisons are very difficult to make, as multiple aspects of technical nature affect the result. Therefore, we have removed this comparison from the main text of the discussion.*

5.	It would be helpful for the authors to provide RIN values alongside the bioA results in figure 3G-K. It is claimed that the "Keeping cells on ice effectively prevents this effect", however it is evident from figure 3 that degradation (although slow) still occurs. Adding the RIN values would help to more definitively clarify this.

*We have now added the RINs, and a quantitative analysis of these with respect to the Bioanalyzer profiles. However, please keep in mind that RIN values have to be interpreted with caution. RIN is a proprietary algorithm from Agilent that was trained with vertebrate samples, but often fails in other organisms. Furthermore, a phenomenon known as the “hidden break” of the 28S RNA affects the RIN number in invertebrate species. This is triggered by denaturation, heating or chemicals such as DMSO (which we use). The hidden break decreases the RIN number, without an actual decrease in RNA integrity. As a result, ACME samples that have been cryopreserved never go above 8, but their RNA quality can nonetheless be very high. To substitute the RIN in the samples where Bioanalyzer failed to calculate it, we have taken a simple approach: measuring the signal area of the 2 ribosomal bands vs the total. This robustly correlates with the RIN (see Supplementary Figure 2), and allows us to infer those not calculated. This approach will be useful for many other organisms where RIN calculations give problems and/or inaccurately represents RNA integrity. We have made these considerations in the main text of the manuscript. Keeping the cells on ice up to 6h is done only for the sake of the demonstration of the method, and most applications can be done with a considerably lower time.*

*Furthermore, from what this reviewer can tell it appears that no replicates were used for this part of the experiment. The number of replicates should be included. It also does not appear that methods for these tests were included in the methods section. They need to be included.*

*In this experiment, we wanted to capture the dynamics in cold and RT conditions, instead of accurately measuring the individual values at each time point to obtain a precise degradation rate. To capture the dynamics, we obtained up to 7 samples of each condition. Our point here was that RNA degradation occurs at a slower rate in cold conditions, a point that we hope the reviewer agrees can be proven with these 14 samples.*

*We have now added all the information requested in a new section of the methods.*

6.	No comparative analysis was done for the above with other dissociation/fixation methods. To justify the authors' claim, they have to show that RNA quality is better maintained in comparison to other dissociation-fixation methods such as PFA in SPLIT-seq or fresh dissociation methods such as Trypsin.

*Thank you for the suggestion. We now show that ACME maintains RNA integrity, while using PFA as a fixative severely affects RNA integrity. This is true both when using ACME as a fixative of trypsin dissociated cells, and when using ACME as a cell dissociation-fixation approach. All of our RIN values obtained with ACME in different conditions are higher than any RIN when formaldehyde is used. Our best RNA integrities values (7.8-9, which is likely at the highest end considering that Platyhelminthes are affected by the “hidden break”, as explained in point 5) are achieved by ACME as a cell dissociation-fixation approach.*

7.	Please demonstrate that ACME is compatible with other sequencing platforms as suggested in the discussion. It is a very narrow application to be focused on planarians and SPLIT-seq if this is a manuscript claiming adaptability of the method.

*To address this important point, we now show a Nematostella vectensis cell type atlas (equivalent largely recapitulating to that published by Sebé-Pedrós et al. Cell 2018) generated with a droplet-based method (10X Genomics).*

8.	As a far as this reviewer can tell, no supp. data show that the scRNA-seq experiments have been repeated (n=1 experiment). It has not been shown that this method can give good results consistently.

*The reviewer is concerned that we did only one split-seq seq reaction. We hope the reviewer understands that, due to the cost, we did not deem it necessary. First, please keep it mind that we use our scRNA-seq data to compare with previous results, and this comparison (with Plass et al) show a very high similarity. Therefore, we do not see how doing an independent replicate will significantly improve this conclusion. Moreover, we have now added a similar comparison for Nematostella, again showing very comparable results with previous data. We hope the reviewer agrees that this addresses the concern: we consistently get good results (comparable to previous organism level cell atlases) using ACME-dissociated cells of two different organisms and two different single-cell transcriptomic platforms. Please keep in mind that our manuscript focuses on describing ACME, not the scRNA-seq techniques or their results themselves. To evaluate ACME, we present multiple independent experiments throughout the manuscript, showing very large consistency across replicates and conditions. Therefore, we strongly believe that we have shown that the method we describe can give good results consistently.*

9. What is the novel application of this method? SPLIT-seq already utilizes fixation of cells and it is apparent that PFA is a standard fixation method, so fixation cannot be the novel factor here. I suppose the novelty could be the handling artefacts introduced during dissociation. The authors spend an entire paragraph in the background section going over how other methods for dissociating and processing cells can result changes in cell's gene expression patterns and thus there is a need for simultaneous dissociation and fixation. This claim is then echoed in the results section where the authors state that the decoupled enzymatic dissociation followed by processing or cryopreservation will result in biological effects that can be observed due to cells being out of their cellular context for several hours. This is again restated in the discussion section: "ACME is a cell dissociation approach that fixes cells while they are being dissociated... disrupting the cellular environment has effects on cellular transcriptomic profiles." However, the authors do not provide any evidence that their method does not also result in changes (or even minimizes changes) in cells' transcriptomic profile. What the authors do show is that they detect the same cell types as in prior bodies of work, not changes in cell state.The authors also show bioA plots of RNA. This does not constitute validation that the transcriptomic profile is not changing as it is still unknown if the same transcripts are being expressed or present in similar quantities to pre-ACME treatment or if they are different transcripts that happen to be similar in size. Analysis needs to be conducted on potential changes in cell states (aka validate with non-dissociation-based methods).

*We present a method for dissociation that relies on a principle not previously applied to single-cell transcriptomics: acid/methanol dissociation. All previous dissociation methods for single-cell transcriptomics relied either on enzymatic/mechanical dissociation or nuclei isolation as principles. These have caveats that have been discussed in the manuscript and elsewhere in the literature: enzymatic/mechanical dissociations require lengthy incubations with the cells live, and nuclei isolation eliminates the cytoplasm, where the vast majority of cellular mRNAs reside. In this regard, ACME neither requires lengthy live incubation nor it eliminates the cytoplasm, and therefore is not affected by the caveats of previous methods. Apart from this advantage, we present many more advantages of using ACME throughout the paper: cells can be easily cryopreserved early during the process, and several times, they can also be stained and sorted, and they can be subjected to both droplet-based and combinatorial methods of single cell transcriptomics. We also show now that ACME preserves RNAs of higher integrity than those obtained by formaldehyde fixation. We expect that our manuscript will lead to novel applications and further developments of the technique. For instance, early preservation can be applicable to collecting animals in the field, where infrastructure for enzymatic cell dissociation and/or FACS is not present. It can also facilitate collaboration between multiple groups, with one performing the dissociation and another one the molecular barcoding. Since ACME relies on a new principle, it will open the door to developing further the technique. For instance, we could see labelling procedures other than the nuclear/cytoplasmic stain we used here (for instance using antibodies).*

*One major novelty is that the dissociation + fixation does not require lengthy live incubations. ACME consist of a fixative with 1 part in 10 of glacial acetic acid (normality ~1.7 N) and it is hard to conceive that cells will be able to transcribe stress genes in such conditions. Contrary to ACME, in trypsin dissociations or other enzymatic and mechanic dissociations cells are alive for hours and this has been shown by other authors to induce stress. However, it was not our intention to analyse trypsin dissociation in detail as this has been done by others.*

10.	The author demonstrate that the composition obtained via ACME is different (better) using scRNA-seq than standard dissociation approaches. This can arise from an aggregate problem as stated above. These data should be validated with non-dissociation-based methods such as IF microscopy.

*Sorry for the misunderstanding; we did not claim that the composition obtained via ACME is “better”, as this is very difficult to evaluate without a gold standard. While the referee is right in arguing that aggregates can affect this, it should be noted that trypsin might affect differentially other cell types, as it generates a lot of cell debris. In the end, both methods likely affect the cell type compositions in different ways and future research will show how to best accurately measure the cell type composition. A cell type atlas only gives an account of the set of cell types as well as their approximate relative abundances. The fact that these approximate abundances are comparable to those obtained by microscopy-based studies (as they were in Plass et al., with datasets obtained by trypsin) indicate that using this approach is a promising method for quantitatively measuring cell type composition. We only claim that “our S. mediterranea dataset contains ~22% neoblasts, in line with microscopy-based estimates (42), obtained by the classic maceration technique”, a claim that is numerically true (they reported 18-32% depending on the planarian size).*

11.	It is this reviewer's opinion that if the authors are going to spend a paragraph in the results going over RNA integrity during ACME and then a full paragraph in the methods on how the RNase activity of the BSA used will affect the results, then they should include the vendor and catalog number of the BSA used. For any new methods paper including information on what reagents were sourced from where would be important to any lab looking to implement ACME in their own research settings. This information would also be good to include for all other reagents necessary to the ACME fixation/dissociation step.

*We have now added the information requested*

0.	This original SPLiT-Seq protocol had a mixing rate of 0.1% (https://science.sciencemag.org/content/360/6385/176.long) on unfixed cells. The ACME method appears to result in higher (2.8%) levels of mixing. If the cells are fixed, how has the sample mixing rate increased by over 20x?

*This depends on the number of cells profiled, and the combinations of barcodes available. The more cells, the more probabilities that two cells share a barcode (collision). Conversely, the more barcodes the less the collision probability. Our combinations are similar to those of Rosenberg et al (48x96x96x3 in our experiment). However, in the experiment the reviewer mentions Rosenberg et al analysed 1,758 cells, and we analysed ~33 thousand cells, approximately 20 times more. This results in an increased mixing rate, as expected.*

Minor:

1. In providing background (paragraph 4) into how maceration with ACME has previously been used to produce fixed cells, it would be helpful for the authors to include a brief explanation on how the acetic acid, methanol, and glycerol works to dissociate soft tissues.

*The original papers on the maceration solution are extremely shallow in their explanation of how the method works. We think it is reasonable to hypothesize that the acetic acid denatures proteins generally and this causes decreased cell adhesion. However, we do not have solid evidence on this.*

Reviewer #3: In the manuscript by Garcia-Castro, et al., the authors describe a new method for performing single cell sequencing on fixed cells, which they term ACME. ACME is a modification of an older dissociation/fixation method and then adapted to single-cell sequencing technology. The authors demonstrate that their technique holds up against previously published data in planarians, in addition to showing that it can be used for preserving samples in the field. It is likely that this technique will be of high interest to the single-cell genomics community. The data are of high quality and mostly well-controlled. I believe that the manuscript needs a bit more work before publication in Genome Biology.

*We thank the reviewer for these comments and hope that the new pieces of work provided, comprising three new main figures and four new supplementary figures warrant now publication.*

Major points

1.	In Figure 1B-F, it would be helpful to include ACME dissociated cells stained with Hoechst to demonstrate the spread of cells by Hoechst red vs Hoechst blue fluorescence, as is commonly done in the planarian field. In B, E and F, the G1 and G2 populations appear as clearly separated populations, but cells in S (which should be visible between the G1 and G2 populations) appear to be missing. Including a Hoechst plot would increase confidence that the ACME treatment doesn't substantially alter the expected spread of cells. Similarly, including a flow plot for ACME treated cells from lethally irradiated animals would be useful for confirming the cell cycle status of these populations (the G2 population should be gone by 1-2 days post-irradiation).

*We have added a comparison of Trypsin and ACME dissociated cells that shows that ACME dissociated cells tend to have more aggregates. The reviewer raises a very interesting point: the use of ACME to quantify the cell cycle. We did not want to claim in the manuscript that ACME can be used to accurately measure the proportions of G1 and G2 cells to study the cell cycle, and we only mention that “the relative proportions of G1 vs G2 cells are similar to those described in planarians by enzymatic approaches”. Unfortunately, the high aggregate proportion of ACME cells makes quantitative analyses difficult. The singlet gates that we apply are standard in cell cytometry – but can substantially alter the proportions of G1 and G2 if applied in more or less stringent ways, since G1 doublets fall in the same DNA staining regime as G2 cells. Altering the gates can lead to slight changes in proportions, that nonetheless remain around ~10% and are therefore “similar to those by enzymatic approaches”. Worth noting, the proportions of X1 (~G1 cells) populations also vary considerably in different papers using trypsin dissociations (See Note below). These differences likely arise from small experimental differences in dissociation, staining and gating conditions. In the revised version of the manuscript we provide an irradiated sample where G2 cells decrease, while they do not completely disappear. This could arise from the fact that ACME cells have a higher aggregate proportion. Further standardisation is needed to measure with confidence the proportions of G1 and G2 and we believe that ACME could be used for this, but for the purpose of this manuscript, we simply wanted to use cytometry and FACS to purify single cells from debris and aggregates.*

*Note: Hayashi et al. described the technique, and specified that the X1 population was “about 20% of cells”, but other papers describe a value between 9% and 13%. For instance, Shiroor et al. Current Biology 2020 recently reported a 12.5% using Hoechst red vs blue, Peiris et al. Regeneration 2016 report from 9.3% to 13.1% using Draq5 and Solana et al. PLoS Genetics 2013 report values of 9¬10% in different control RNAi replicates.*

2.	In Figure 3G-K, the effects of the different treatments on the 28S and 18S RNAs are clear; however, these RNAs are much more abundant than the mRNAs we want to isolate for sequencing. So while these highly abundant RNAs survive these challenges (with some degradation), this does not necessarily mean the mRNA profiles of the cells are acceptable for sequencing or reflective of the live state. Thus, a quantitative measure of RNA integrity via bioAnalyzer traces and RNA Integrity Numbers (RIN) should be provided.

*We now include RIN values. As stated in our response to reviewer 2 above, these have to be interpreted with caution as in many invertebrates such as S. mediterranea a phenomenon called the “hidden break” decreases the RIN number without an actual decrease in RNA integrity. In the new version of the manuscript we also show that ACME consistently results in far greater RNA integrity when compared to formaldehyde. Of note, the original SPLiT-seq paper used formaldehyde as a fixative and obtained excellent results, as have been obtained by similar methods, providing the single-cell transcriptomes of tens of thousands of cells. This shows that RNA that has severely lost integrity still yields excellent single cell data.*

3.	A head to head comparison of ACME with other dissociation/fixation protocols would be useful to determine whether this method outperforms other methods (for example, by comparing flow plots, bioAnalyzer traces, RIN).

*We have now compared ACME as a dissociation method to trypsin (Supplemental Figure 1) and as a fixative to formaldehyde (Figure 4).*

4.	As split-seq has not yet been performed in planarians, the authors should strongly consider adding to figure 2 the ability to correctly resolve cell types from diverse animals - not just 2 species of planarians. Overall Fig 2 is quite thin and needs more data than just dissociation and flow plots.

*We now provide the single-cell data for yet another species, the cnidarian Nematostella vectensis, in this case produced using a droplet-based method. This data is presented in the new main Figure 4. We have added two more species to Figure 2 and have provided ungated profiles to show that the populations can be easily obtained with simple gating conditions. Furthermore, we have complemented Figure 2 with a visual representation of the animals selected. We believe that this supports the main point of the figure, which is not only that we manage to dissociate several animals, but that these are diverse, from diverse habitats and life stages. We believe that the new Figure 2 conveys in a more powerful way how versatile ACME dissociation is and we hope that the reviewer agrees with this.*

Minor points

1.	In Figure 3F, population percentages should be provided (as in B-E) to demonstrate quantitatively whether one population is more sensitive to freeze/thaw cycles than the other.

*As stated above, we do not want to use our cytometry plots to quantify differences between G1 and G2 populations, as we believe these are strongly influenced by gating conditions. We provided the population percentages in the previous figure (Figure 3B-E) exclusively to show that FACS allows us to go from ~30% to ~90% of whole cells.*

2.	Is there an explanation for why sub-library 3 had so few cells pass the thresholds despite having similar read depth?

*Library 3 had a higher number of PCR cycles to reach the plateau phase during the amplification (specifically, 4 more cycles than Library 1 and Library 2) and this caused a higher amount of PCR duplicates. Since duplicate UMIs provide no further useful information, with the same sequencing depth we find less cells at the same cutoff threshold.*

3.	In Figure S2, showing the whole UMAP plot in each panel would make it easier to understand the relationships of the clusters.

*We have changed this.*

4.	In fig 3B - why is there considerably less debris after freeze-thawing? Are there additional washes that take away debris? One would predict that more freeze-thawing would yield more debris.

*Figure 3B depicts a normal ACME reaction, prior to any freezing-thawing. In Figure 3C, the level of debris after freeze-thawing is similar (62% in A, 66% in B). We never get a significantly higher yield of debris after freezing-thawing. Some cells may break during this process, but after thawing we normally perform 2 washing centrifugations, so the total debris remain equivalent to that found in the pre-frozen sample. Then, Figure 3D has considerably less debris (14%) since it is FACS sorted, the purpose of the FACS enrichment process is to purify cells from the debris. Figure 3E has slightly less debris after freezing-thawing the FACS sorted populations (9%), but this is hardly significant. Our point in this set of cytometry-FACS analyses is that we can obtain highly pure (85-90%) single cells by FACS, even using a two freezing step approach.*

5.	Page 9, lines 9-20. You state several times that the japonica genome assembly is worse, so that hinders your sequence alignments to this species. Did you try the transcriptomes of both species instead? I believe those are of quite high quality on the planmine database.

*We have tried mapping our reads to transcriptomes. This approach works, but generally we map less reads, as a significant portion of the reads map to introns or exon-intron boundaries, and not all genes are necessarily present in a transcriptomic resource. Consequently, this also reduces the quality of the cell type atlas.*

6.	Consider a new results section for Fig 5 on its own to help readability.

*We have now changed this and added a number of other sections.*

7.	Page 9, line 43 "Smedwi-1" should be all small letters. *Changed*

8.	The authors should consider a more in depth analysis of the germ cell precursors since this is the first study to detect them.

*We provide more evidence in this manuscript on their detection and have provided a marker list in the supplemental data. We believe this will enable further research on the subject.*

9.	It is unclear in Fig 1B why presumably larger cells (higher Con-A) with the same DNA content are not included in the G2 and G1 gates. Those cells at the same DNA level shouldn't be aggregates, so what are the criteria for excluding them?

*This is the product of the singlet filter: a doublet consisting of G1 cell and piece of debris falls in G1 but has more cytoplasm. Similarly, a doublet of two G1 cells falls in G2 but has higher cytoplasm staining. The singlet filter removes these because of their high signal area vs height relationship.*

**Second round of review**

**Reviewer 1**

In the revised manuscript and their response letter, I found some of my concerns addressed well. However, for some of the points I’m not convinced by the additional analyses and data provided by the authors.

They still did not include the number of cell barcodes with >5,000 UMIs, which were discarded as doublets/aggregates in their planarian sequencing experiments. They should report this number, since this will give an estimate of how many aggerates remain after FACS. These are unlikely to correspond to collisions as the average UMI count per cell is <1,000 and, hence, these are probably aggregates containing multiple cells.

I appreciate the effort of the authors conducting an additional 10x single-cell RNA-seq experiment on another species. However, I am surprised by the low sensitivity which is even lower than for MARS-seq. Notably, a recent benchmarking study demonstrated the low sensitivity of MARS-seq compared to almost all other common single-cell RNA-seq protocols, in particular, in comparison to 10x Genomics (Mereu et al., 2020, Nature Biotechnology). This suggests that a substantial fraction of mRNAs might be lost during ACME dissociation. Alternatively, the lower sensitivity could be explained by the reduced sequencing depth of the 10x library compared to the MARS-seq library. But the authors don’t give any numbers here. To make a solid comparison of UMIs per cell and numbers of genes recovered by each method, they have to down-sample MARS-seq and 10x sequencing data to the same number of reads.

Along the same lines, the authors state in their response letter, that the Plass et al. data have the same UMI per cell distribution like their own dataset. If this is not controlled for sequencing depth, this information cannot be interpreted. Hence, they have to compare UMI counts and median number of genes recovered per cell after down-sampling both datasets to the same read depth.

In general there is no consistent comparison contained in the manuscript using ACME versus another established digestion method with the same sequencing technology. Hence, it is still impossible based on the data contained in the revised manuscript to assess if ACME leads to superior or at least comparable data quality. I expected more insights from the comparison to the Plass et al. data, although this would still be confounded by differences in sequencing technologies. I am aware that it is likely not possible to perform a direct differential gene expression analysis between cells from the two datasets, even after Seurat integration. However, the authors could at least perform this analysis, e.g., on mature cell types versus neoblasts for each dataset separately, and compare the number, expression levels, and functional categories of differentially expressed genes obtained by the two different pipelines. And this comparison should be controlled for sequencing depth as well.

**Reviewer 2**

Overall, the authors addressed most for my comments in this revision. We only have a few minor comments remaining. While it is hard to quantify whether “native cell state” is maintained during simultaneous dissociation and fixation, the authors at least convinced me that the method is an improvement in terms of RNA quality over PFA used in SPLIT-seq. The inclusion of figure 5 and the associated part in the results section (Single-cel transcriptomic analysis of cnidarian ACME-dissociated cells using a droplet-based method) is of import as it helps the reader to gain context for the versatility of ACME. Inclusion of this data will likely lead to a wider adoption of this fixation method, due to the prevalence of droplet-based methods. Furthermore, figure 5 would seem to indicate that the low library diversity compared to other reported values might be more due to the organism being studied than the dissociation method.

Major:

1. The point that ACME allowed the authors to identify a population of nanos-positive cells previously only described in non-single-cell studies is well taken. It is one of the biggest arguments in the paper for the potential of ACME, since other studies were unable to identify this population. The label transfer analysis helps to show that these cells are the same population that was present in prior datasets but mixed in with other cell populations. However, we would still like to see some validation that the FACS sorted cells that went into the analysis are devoid of clusters. i.e. the unique neoblastic cell population can really be identified from single cells and not as a unique population due to mixing of cells from sequencing a cluster.

Minor:

1. It is unclear if there is a discrepancy between the figure and the text of the results. Specifically given that figure 5C parts are violin plots, if figure 5C right is to be believed, then there seems to be no way that the median number of genes detected per cell in the ACME treated sample could be 106 genes per cell as mentioned in the text. Based on the figure, I would have expected the median to be above 250 genes per cell. I ask that the authors clarify this point.

2. The figure legend for supplementary figure 3 does not match up with which portions of the supplementary figure are described in the figure legend. There is also a link that is present in the middle of the figure that stays there regardless of which browser was used to download it or which program used to view the PDF. Recommend removing said link and fixing figure legend.

3. The use of the word “novel” in “Remarkable, we found a novel neoblast cluster…” implies that these cells are being described for the first time. If they have been previously been characterized in other literature then they are not necessarily novel even if it is the first time they are being seen in a single cell manner. Recommended rephrasing this or removing the word novel.

**Reviewer 3**

In the revised manuscript by Garcia-Castro, et al., the authors have improved the manuscript and addressed many issues from the previous version. My main concerns have been addressed, and the changes to incorporate all reviewer comments have made the manuscript much more widely appealing to the field. I have only a few textual comments.

My first comment of the missing X1 cells in S-phase, remains unaddressed (the textual answer was not in line with the comment). The plots in Fig. 3B-F look as though they may now contain all S-phase cell too. A true DNA stain of cells in an asynchronous population will give DNA values spread throughout the G1-S-G2 phases, so it is unclear why this is not so with DRAQ5 (and why a Hoechst red vs. blue plot was requested). Because the S-phase cells cannot be resolved, this is not a huge deal, but certainly calling the high-DNA populations of cells “G2” is not appropriate in this case as it contains S-G2-M phase cells based on irradiated plots. Perhaps something like “>2C DNA”.

Page 6, line 45 (and methods section too) – the reference for NAC is incorrect as this was first reported for planarians in Pearson, et al., 2009, Developmental Dynamics.

I do not particularly care for the new Figure 4 as a stand-alone conclusion of data.

**Authors Response**

**Point-by-point responses to the reviewers’ comments:**

Reviewer #1:

In the revised manuscript and their response letter, I found some of my concerns addressed well. However, for some of the points I'm not convinced by the additional analyses and data provided by the authors.

*Many thanks for your thoughts and time. We have provided additional data to further address the points raised below, which we feel will fully answer your remaining concerns.*

They still did not include the number of cell barcodes with >5,000 UMIs, which were discarded as doublets/aggregates in their planarian sequencing experiments. They should report this number, since this will give an estimate of how many aggerates remain after FACS. These are unlikely to correspond to collisions as the average UMI count per cell is <1,000 and, hence, these are probably aggregates containing multiple cells.

*We believe the reviewer missed these numbers, which were already noted on page 9 of our manuscript (234 and 177 cell barcodes excluded in S. mediterranea and D. japonica respectively). To make this more obvious we have rewritten this section to read:*

*“We also discarded very high UMI containing cells barcodes (>5000 counts) to prevent the inclusion of aggregates that may have remained after FACS purification, as well as cells sharing the same cell barcodes through “collisions” (234 and 177 cell barcodes excluded in S. mediterranea and D. japonica respectively).”*

I appreciate the effort of the authors conducting an additional 10x single-cell RNA-seq experiment on another species. However, I am surprised by the low sensitivity which is even lower than for MARS-seq. Notably, a recent benchmarking study demonstrated the low sensitivity of MARS-seq compared to almost all other common single-cell RNA-seq protocols, in particular, in comparison to 10x Genomics (Mereu et al., 2020, Nature Biotechnology). This suggests that a substantial fraction of mRNAs might be lost during ACME dissociation. Alternatively, the lower sensitivity could be explained by the reduced sequencing depth of the 10x library compared to the MARS-seq library. But the authors don't give any numbers here. To make a solid comparison of UMIs per cell and numbers of genes recovered by each method, they have to down-sample MARS-seq and 10x sequencing data to the same number of reads.

*Our thanks to the reviewer for this suggestion. To comprehensively answer these concerns, we have downsampled our MARSseq reads to the same read count per cell (15k mapped reads) as seen in the 10x derived data. This results in very similar metrics between previous MARS-seq experiments and our data. In particular, our ACME-derived 10x data shows a similar UMI count (671 vs 696 UMI) and similar, but slightly improved gene per cell count (418 vs 381 genes) compared to MARS-seq data. We have also updated the violin plot in Fig 5 to show this. We would like to point out that a study of Nematostella cells dissociated enzymatically (TrypLE, a recombinant enzyme) and sequenced with 10X technology had very comparable UMI per cell and gene per cell statistics. We have cited this study. We can therefore conclusively state that there is no evidence of lower sensitivity, and do not believe that a substantial fraction of mRNAs might be lost during ACME dissociation. We have now added this additional information to the text:*

*“Our ACME-dissociated dataset recovered a median of 671 UMI per cell and 418 genes per cell (Figure 5C, Supplementary Figure 3C), resembling the statistics obtained in the previous study (833 UMI per cell and 464 genes per cell, and 696 UMI per cell and 381 genes per cell when downsampled to comparable number of reads) generated using enzymatic (Liberase) dissociations (10) and MARS-seq (57). A recent single cell transcriptomic study of N. vectensis cells dissociated enzymatically and barcoded using 10X Genomics technology had comparable statistics (58). "*

Along the same lines, the authors state in their response letter, that the Plass et al. data have the same UMI per cell distribution like their own dataset. If this is not controlled for sequencing depth, this information cannot be interpreted. Hence, they have to compare UMI counts and median number of genes recovered per cell after down-sampling both datasets to the same read depth.

*We thank the reviewer again for this recommendation. To provide a robust comparison of UMI counts and median number of genes we have downsampled our data in a number of ways. In particular, we needed to account for the presence of a large number of erroneous, adapter containing, short and N rich reads in our raw data. These sequencing errors are independent of ACME, and are often removed by the sequencing provider and would have been reduced by adjusting SPRI selection conditions of the library construction. After removing these reads, mapping statistics were very good (Supplementary Figure 8). The Drop-seq experiment suffers from lower mapping statistics, with only a third of the reads mapping to our new improved annotation of the genome, also likely a product of such complex library preparation.*

*We then performed two downsampling analyses, each of which provides information pertinent to the reviewer’s request:*

*-	Statistics after downsampling García-Castro raw reads to 1,200,007,715 (Plass total). This comparison is a direct comparison of raw read numbers.*

*-	Statistics after downsampling both to 10k mapped reads per cell. This comparison uses only reads after mapping and takes into account the cell numbers.*

*Full statistics of these analyses are provided in Supplementary Figure 8. From both of these comparisons, it is evident that our ACME-derived data has comparable or better UMI per cell recovery rate, but a poorer gene per cell recovery rate. For instance, in the downsampling to 10k mapped reads per cell, our ACME-dissociated dataset recovers a median of 723 UMI per cell (compared to 594 in the Plass et al. dataset) and 176 genes per cell (compared to 394).*

*To explain these differences, there are four important considerations to take into account, all related to the scRNA-seq method (Drop-seq vs SPLIT-seq) and experimental design, and thus independent of ACME:*

*-	Our experiment was a mixture of 2 species, Schmidtea mediterranea and Dugesia japonica. The genome and gene models of the latter are poorer, and this results in unmapped reads and reads mapped to unannotated regions, reducing the number of UMIs and genes detected per cell. This does not happen in the Plass et al. results, which contains only Schmidtea mediterranea.*

*-	Drop-seq uses an oligo-dT(30), while the SPLiT-seq has an oligo-dT(15). Planarians are A/T rich, and these different configurations can cause differences in internal priming, which would more severely affect A/T richer introns.*

*-	Drop-seq has a 64bp read length (which may lead to poorer mapping results), and SPLiT-seq 150bp. Planarian introns are relatively small (in a sharp bimodal distribution, please see Solana, Irimia et al. eLife 2016). SPLiT-seq reads are much more likely to hit splice sites, since they are longer.*

*-	Likely as a result of the previous two points, our ACME-SPLiT-seq experiment has considerably more intron content: 23.67% (81,299,126 of 343,409,649) of the total reads mapping to genes from Plass et al. mapped to intronic locations (including splice sites), compared to 37.83% (161,656,944 of 427,296,897) of the total ACME reads mapped to intronic locations. This number goes up to 55.66% (132,181,178 of 237,451,599) of the total reads that mapped to the Schmidtea mediterranea genome.*

*To explain the relatively lower gene per cell recovery with similar UMI per cell we could advance two lines of argumentation:*

*1) our ACME-SPLiT-seq has more nuclear pre-mRNA compared to cytoplasmic mRNA. This could be a product of the technical conditions above (oligo-dT, read length, rapid fixation) or be alternatively caused by ACME enriching for nuclear RNA relative to the particular trypsin treatment done in Plass et al. Importantly, this is not a concern for ACME as a dissociation method; in fact, many researchers opt for nuclei isolation (which eliminates all cytoplasm, and is enriched in pre-mRNA). Alternatively, it could be that SPLiT-seq recovers more introns in general (as has been shown in a recent study: https://doi.org/10.1101/632216 see Supplementary Table 3). Both of these scenarios would equally result in more UMIs and less genes: pre-mRNAs are longer than mRNAs, and the same gene can generate more UMIs. Fewer genes will therefore be observed for a given number of UMIs.*

*2) an alternative explanation is that the Drop-seq experiment could have suffered from more ambient RNA. Broken cells release RNAs that are loaded into the droplets, together with the whole cells. This is in fact harder to conceptualise in the SPLiT-seq approach, where any free RNA would be eliminated with the supernatant in each of the cell pelleting steps after the barcoding rounds. Then, in the Drop-seq experiment, each cell-containing droplet has a cell, with cell specific RNAs, and a portion of ambient RNA, with RNAs from all cells mixed. With the same UMIs, each cell barcode appears to have more genes. In this scenario, if ambient RNA is maximised, all droplets would ultimately contain all genes.*

*We believe it is impossible to decide between these two. The fact that with a lot less genes quantified but similar UMIs, our ACME dataset seems to retrieve the same or more information content (cell clusters) make us favour the second explanation. Be it as it may, after downsampling and despite recovering less genes, our ACME experiment stands very well against the comparison to Plass et al. and robustly recovers the information in that original paper. We offer this longer explanation here, but summarise the established facts in the main manuscript:*

*“To compare UMI and gene per cell statistics in the two datasets we performed downsampling experiments (Supplementary Figure 8). When both datasets are downsampled to 10K reads per cell our ACME-dissociated dataset still recovers a median of 723 UMI per cell (compared to 594 in the Plass et al. dataset) and 176 genes per cell (compared to 394). This relatively lower gene recovery can be attributed to several causes, including a comparatively higher recovery of intronic regions in the SPLiT-seq dataset (37.83% compared to 23.7% in the Plass et al. dataset, 55.7% if only S. mediterranea reads are considered).”*

In general there is no consistent comparison contained in the manuscript using ACME versus another established digestion method with the same sequencing technology. Hence, it is still impossible based on the data contained in the revised manuscript to assess if ACME leads to superior or at least comparable data quality. I expected more insights from the comparison to the Plass et al. data, although this would still be confounded by differences in sequencing technologies. I am aware that it is likely not possible to perform a direct differential gene expression analysis between cells from the two datasets, even after Seurat integration. However, the authors could at least perform this analysis, e.g., on mature cell types versus neoblasts for each dataset separately, and compare the number, expression levels, and functional categories of differentially expressed genes obtained by the two different pipelines. And this comparison should be controlled for sequencing depth as well.

*We have added an additional supplementary figure (Supplementary Figure 9), showing the results of differential gene expression analyses as requested. Please note that sequencing depth normalisation was performed as a part of this procedure, as is standard. We have performed 5 comparisons of neoblasts vs mature cell types in both the Plass et al. analysis and in our own dataset. These showed an exceptional level of overlap. Around 80% of differentially expressed genes are common between our and the Plass et al. sets at a common cutoff for significance. These exhibit very similar average log fold change values. This strongly supports the reproducibility of these analyses.*

Reviewer #2:

Overall, the authors addressed most for my comments in this revision. We only have a few minor comments remaining. While it is hard to quantify whether “native cell state” is maintained during simultaneous dissociation and fixation, the authors at least convinced me that the method is an improvement in terms of RNA quality over PFA used in SPLIT-seq. The inclusion of figure 5 and the associated part in the results section (Single-cel transcriptomic analysis of cnidarian ACME-dissociated cells using a droplet-based method) is of import as it helps the reader to gain context for the versatility of ACME. Inclusion of this data will likely lead to a wider adoption of this fixation method, due to the prevalence of droplet-based methods. Furthermore, figure 5 would seem to indicate that the low library diversity compared to other reported values might be more due to the organism being studied than the dissociation method.

*We thank the reviewer for the positive comments. We agree that the inclusion of Figure 5 will aid in proving the utility of this work to a broader audience.*

Major:

1. The point that ACME allowed the authors to identify a population of nanos-positive cells previously only described in non-single-cell studies is well taken. It is one of the biggest arguments in the paper for the potential of ACME, since other studies were unable to identify this population. The label transfer analysis helps to show that these cells are the same population that was present in prior datasets but mixed in with other cell populations. However, we would still like to see some validation that the FACS sorted cells that went into the analysis are devoid of clusters. i.e. the unique neoblastic cell population can really be identified from single cells and not as a unique population due to mixing of cells from sequencing a cluster.

*To assure the reviewer that these cells do not derive from a cluster made up of high UMI cells (caused by aggregates or barcode collisions) we have added Supplementary Figure 7 to this work. This shows:*

*-	UMAP plots of the data without removal of high UMI cells. These show that the highest UMI cells are found away from the nanos-positive germ cell precursor cluster (17)*

*-	UMAP plots of the data after removal of high UMI cells, showing the number of UMIs per cell. Note that the germ cell precursor cluster does not have a high UMI content relative to other cells.*

*We trust that this fully addresses this concern.*

Minor:

1. It is unclear if there is a discrepancy between the figure and the text of the results. Specifically given that figure 5C parts are violin plots, if figure 5C right is to be believed, then there seems to be no way that the median number of genes detected per cell in the ACME treated sample could be 106 genes per cell as mentioned in the text. Based on the figure, I would have expected the median to be above 250 genes per cell. I ask that the authors clarify this point.

*The reviewer is entirely correct, and this number, and the gene number, is erroneous due to a mathematical error. Apologies for the oversight – the correct values have been added and this section now reads:*

*“"Our ACME-dissociated dataset recovered a median of 671 UMI per cell and 418 genes per cell (Figure 5C, Supplementary Figure 3C), resembling the statistics obtained in the previous study (833 UMI per cell and 464 genes per cell, and 696 UMI per cell and 381 genes per cell when downsampled to comparable number of reads) generated using enzymatic (Liberase) dissociations (10) and MARS-seq (57)."*

2. The figure legend for supplementary figure 3 does not match up with which portions of the supplementary figure are described in the figure legend. There is also a link that is present in the middle of the figure that stays there regardless of which browser was used to download it or which program used to view the PDF. Recommend removing said link and fixing figure legend.

*The reviewer is correct – this has been shuffled a little in the submitted version. We have corrected the text to read:*

*“A: Flow cytometry profiles of ACME-dissociated cells from Nematostella vectensis, showing all events (left) and G1/G0 singlets (right). B: Expression of 514 variable genes (rows) across 3,899 juvenile N. vectensis single cells sorted by metacell and cell type association. Top 30 genes (FC>2) per metacell are included, showing gene functional annotation when available. Transcription factors are highlighted in red. C: Total number of RNA molecules (left) and cells (right) per metacell.”*

*We apologise for the appearance of the link. We have deleted this from the re-uploaded version of this file.*

3. The use of the word “novel” in “Remarkable, we found a novel neoblast cluster…” implies that these cells are being described for the first time. If they have been previously been characterized in other literature then they are not necessarily novel even if it is the first time they are being seen in a single cell manner. Recommended rephrasing this or removing the word novel.

*We agree with the reviewer’s point, and have corrected this to read:*

*“Remarkably, we found a neoblast cluster containing nanos-positive germ cell precursor cells that has not previously been observed in single cell atlases (Supplementary Figure 6A)."*

Reviewer #3:

In the revised manuscript by Garcia-Castro, et al., the authors have improved the manuscript and addressed many issues from the previous version. My main concerns have been addressed, and the changes to incorporate all reviewer comments have made the manuscript much more widely appealing to the field. I have only a few textual comments.

*Many thanks for your constructive comments. We hope that the changes below address the remaining concerns in full.*

My first comment of the missing X1 cells in S-phase, remains unaddressed (the textual answer was not in line with the comment). The plots in Fig. 3B-F look as though they may now contain all S-phase cell too. A true DNA stain of cells in an asynchronous population will give DNA values spread throughout the G1-S-G2 phases, so it is unclear why this is not so with DRAQ5 (and why a Hoechst red vs. blue plot was requested). Because the S-phase cells cannot be resolved, this is not a huge deal, but certainly calling the high-DNA populations of cells “G2” is not appropriate in this case as it contains S-G2-M phase cells based on irradiated plots. Perhaps something like “>2C DNA”.

*We agree with the reviewers concern, and concur that this is a minor nomenclature problem. As stated in our previous response, we did not want to claim that ACME can be used to study cell cycle. “2C DNA” or “4C DNA” suffers from a similar caveat, since S-phase cells have a DNA content in between 2C and 4C (in Schmidtea mediterranea, a diploid). Furthermore, because S-phase cells cannot be resolved it is difficult to say if they are in the G2 or in the G1 gate. Thus, we have opted for further clarifying this aspect in the text to clearly state that G2 cells might contain other cell cycle phases. Unfortunately, the FACS facility we are using is not equipped with a UV laser to use Hoechst in a Hoechst red vs. blue plot as requested. Indeed, we originally opted for DRAQ5 as an alternative that can be visualised and sorted more generally in common FACS and cytometry laser configurations. We are sure that further optimisation of the staining conditions will lead to cell cycle studies.*

*The section where we first explain these populations now reads:*

*“These staining conditions reveal several cell populations (Figure 1B). The lowest DNA containing population corresponds to cells with 2C DNA content, and includes G1 and G0 cells (hereafter “G1”). The cell population above contains G2/M cells with 4C DNA content (hereafter “G2”). S-phase cells are difficult to resolve with these staining conditions. G2 corresponds to what planarian FACS protocols typically refer to as the ‘X1’ population (50), and is sensitive to irradiation (Supplementary Figure 1A-B)”*

Page 6, line 45 (and methods section too) – the reference for NAC is incorrect as this was first reported for planarians in Pearson, et al., 2009, Developmental Dynamics.

*The reviewer is correct. We have updated the reference to include the reference, Pearson, B.J., Eisenhoffer, G.T., Gurley, K.A., Rink, J.C., Miller, D.E. and Sánchez Alvarado, A., 2009. Formaldehyde‐based whole‐mount in situ hybridization method for planarians. Developmental dynamics, 238(2), pp.443-450.*

I do not particularly care for the new Figure 4 as a stand-alone conclusion of data.

*We feel that this figure is best placed in the main text, both as a robust proof of ACME’s utility relative to formaldehyde (which will be useful to a range of researchers) and as a clear answer to queries raised in the previous round of review. We considered the reviewer’s position, but prefer to keep it in text for these reasons.*

**Third round of review**

**Reviewer 1**

The authors have sufficiently addressed my concerns with the additional analysis provided in the revised manuscript. However, I'm not convinced by their claim that the difference in the number of recovered genes per cell between Plass et al. and their dataset is attributable to the presence of more nuclear pre-mRNA in their data or to ambient RNA. The confounding factor of ambient RNA could actually be removed computationally, e.g., by running DecontX (Yang et al., 2020, Genome Biology). To quantify a possible effect of more pre-mRNAs on UMIs, the authors should count internal priming sites per gene and compare the distribution between the two studies. In my opinion, this is not strictly required for publication of this manuscript as the UMI/gene count comparison between the datasets is now sufficiently transparent, but the authors need to adjust their statement

"This relatively lower gene recovery can be attributed to several causes, including a comparatively higher recovery of intronic regions in the SPLiT-seq dataset..."

to

"This relatively lower gene recovery can be attributed to several causes, potentially including a comparatively higher recovery of intronic regions in the SPLiT-seq dataset... or differences in the presence of ambient RNA."
